# Sensitivity of Quantitative Susceptibility Mapping for Clinical Research in Deep Gray Matter

**DOI:** 10.1002/hbm.70187

**Published:** 2025-04-22

**Authors:** Fahad Salman, Abhisri Ramesh, Thomas Jochmann, Mirjam Prayer, Ademola Adegbemigun, Jack A. Reeves, Gregory E. Wilding, Junghun Cho, Dejan Jakimovski, Niels Bergsland, Michael G. Dwyer, Robert Zivadinov, Ferdinand Schweser

**Affiliations:** ^1^ Buffalo Neuroimaging Analysis Center, Department of Neurology at the Jacobs School of Medicine and Biomedical Sciences University at Buffalo, The State University of New York Buffalo New York USA; ^2^ Department of Biomedical Engineering University at Buffalo, The State University of New York Buffalo New York USA; ^3^ Department of Computer Science and Automation Technische Universität Ilmenau Ilmenau Germany; ^4^ Department of Biostatistics School of Public Health and Health Professions, State University of New York at Buffalo Buffalo New York USA; ^5^ Center for Biomedical Imaging Clinical and Translational Science Institute, University at Buffalo, The State University of New York Buffalo New York USA

**Keywords:** algorithmic reproducibility, algorithmic sensitivity, brain iron, clinical research, deep gray matter, MRI, quantitative susceptibility mapping

## Abstract

Quantitative susceptibility mapping (QSM) is an advanced MRI technique for assessing iron, calcium, and myelin tissue levels based on magnetic susceptibility. QSM consists of multiple processing steps, with various choices for each step. While QSM is increasingly applied in neurodegenerative disease research, its reproducibility and sensitivity in detecting susceptibility changes across groups or over time, which underpin the interpretation of clinical outcomes, have not been thoroughly quantified. This study aimed to evaluate how choices in background field removal (BFR), dipole inversion algorithms, and anatomical referencing impact the detection of changes in deep gray matter susceptibility. We used aging‐related changes in brain iron, established in earlier foundational studies, as a surrogate model to test the sensitivity and reproducibility of 378 different QSM pipelines toward the detection of longitudinal susceptibility changes in a clinical setting. We used 10‐year follow‐up data and scan‐rescan data of healthy adults scanned at 3T. Our results demonstrated high variability in the sensitivity of QSM pipelines toward detecting susceptibility changes. While most pipelines detected the same over‐time changes, the choice of the BFR algorithm and the referencing strategy influenced reproducibility error and sensitivity substantially. Notably, pipelines using RESHARP with AMP‐PE, HEIDI, or LSQR inversion showed the highest overall sensitivity. The findings suggest a strong impact of algorithmic choices in QSM processing on the ability to detect physiological changes in the brain. Careful consideration should be given to the pipeline configuration for reliable clinical outcomes.

## Graphical abstract

This study aimed to evaluate how choices in background field removal, dipole inversion algorithms, and anatomical referencing impact the sensitivity and reproducibility error of QSM in detecting group‐level and longitudinal changes in deep gray matter susceptibility. Our results demonstrated variable sensitivity of QSM pipelines (*N* = 378) to detect susceptibility changes.

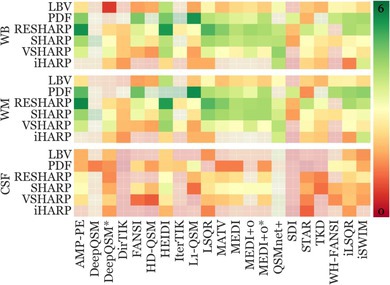

**Summary**

- We analyzed the sensitivity of different quantitative susceptibility mapping (QSM) pipelines (*N* = 378; background field removal [BFR] + dipole inversion + reference region) toward the detection of aging‐related susceptibility changes in the deep gray matter over a period of 10 years. Our results demonstrated high variability in the sensitivity of QSM pipelines to detect susceptibility changes.
- The study highlighted that while most pipelines could detect changes reliably, the choice of the BFR algorithm and the referencing strategy substantially influenced the reproducibility error and sensitivity.
- It is important to consider the performance of the entire QSM pipeline rather than its individual components in isolation.

## 1 Introduction

Quantitative susceptibility mapping (QSM) is a magnetic resonance imaging (MRI) technique that calculates tissue magnetic susceptibility from gradient‐echo phase images (Schweser et al. 2016; Wang and Liu 2015). The technique can be used to assess tissue iron (paramagnetic) (Langkammer et al. 2012), myelin (Deh et al. 2018), and calcium (both diamagnetic) (Schweser et al. 2010). The use of QSM in clinical research is growing rapidly (Madden and Merenstein 2023; Sun et al. 2017), not only in the brain but also in other parts of the body (Aimo et al. 2022; Dimov et al. 2023; J. Li et al. 2018; Schumacher et al. 2024).

In the brain's deep gray matter (DGM), magnetic susceptibility correlates strongly with tissue iron concentrations (Langkammer et al. 2012; Otsuka et al. 2024). Several neurodegenerative diseases with brain iron involvement, including multiple sclerosis (Elkady et al. 2019; Hagemeier et al. 2018; Zivadinov et al. 2018), Parkinson's disease (He et al. 2015; Ide et al. 2015; Langkammer et al. 2016), and Alzheimer's disease (Acosta‐Cabronero et al. 2013; Bulk et al. 2020), show altered magnetic susceptibility in the DGM. QSM's ability to monitor these and other conditions may be highly valuable in clinical trials or in the clinical setting. However, the factors that impact the reproducibility of QSM and its sensitivity toward susceptibility changes remain poorly understood.

QSM involves several processing steps (Schweser et al. 2016), for which numerous algorithms have been proposed over the past decade. The final three steps applied after a field map has been reconstructed from the MRI phase images (Robinson et al. 2017) are: (i) background field removal (BFR), (ii) solution of the dipole inversion problem, and (iii) referencing of susceptibility values to an internal reference point. We will refer to a specific combination of BFR algorithm, dipole inversion algorithm, and referencing region as a *pipeline* throughout this work. BFR algorithms remove field perturbations from sources located outside the region of interest (e.g., the brain in a brain examination). Following the BFR, dipole inversion is performed to derive a tissue magnetic susceptibility map (*χ*) from the background field (BF)‐corrected field map. Subsequently, susceptibility values must be referenced to an internal reference region (Straub et al. 2017) before reporting susceptibility findings (QSM Consensus Organization Committee et al. 2024). While there is consensus that the reference region choice can impact statistical power and introduce bias (QSM Consensus Organization Committee et al. 2024), the optimal anatomical region for referencing remains a matter of ongoing debate (QSM Consensus Organization Committee et al. 2024) and the effect of referencing on group‐level or longitudinal study outcomes is poorly understood.

The QSM community has benchmarked inversion algorithms in two challenges since 2016 (Langkammer et al. 2018; QSM Challenge 2.0 Organization Committee et al. 2021). The first challenge used elements of the susceptibility tensor from a single subject, while the second relied on a single simulated brain scan as a gold standard (Marques et al. 2021). Both challenges provided valuable insights into the state of the field and highlighted methodological challenges, such as substantial differences in susceptibility map appearance between algorithms. However, both approaches were also subject to methodological concerns; for instance, using a single element from the diagonal of the susceptibility tensor neglected field perturbations caused by off‐diagonal elements (Milovic et al. 2020). Furthermore, since the investigations used only a single reference susceptibility map, the *practical* (or clinical) significance of these benchmarking efforts remained unclear. Specifically, it remained unknown if the inversion algorithms with the highest performance in the QSM challenges also offer the best sensitivity toward detecting cross‐sectional group differences or group‐level changes in susceptibility over time (longitudinally), or how the BFR algorithm choice affects the sensitivity.

The overarching goal of this study was to understand how clinical study outcomes are influenced by the specific choices made in the final three steps of the QSM pipeline. Additionally, we aimed to identify the pipelines that detect group‐level susceptibility differences with the highest sensitivity in a typical clinical research setting—a pursuit largely unexplored until now, with an emphasis of previous benchmarking efforts primarily placed on the reproducibility and the accuracy of the dipole inversion step (Deh et al. 2015; Feng et al. 2018; Hinoda et al. 2015; Lancione et al. 2022; Langkammer et al. 2018; Lin et al. 2015; Liu et al. 2024; Naji et al. 2022; QSM Challenge 2.0 Organization Committee et al. 2021; Rua et al. 2020; Santin et al. 2017; Spincemaille et al. 2019; Spincemaille et al. 2020; Yao et al. 2023).

## 2 Theory

The effect size is the relevant statistical quantity that describes the strengths of an observation in a cohort‐based study. The observed effect size $d_{r}$ of a true susceptibility difference Δ*χ*_*r*_ = *χ*_2,*r*_ − *χ*_1,*r*_ between two sample distributions in a region of interest (ROI), *r*, can be defined as

(1) $$d_{r}=\frac{\Delta \chi_{r}}{0.5∙\left(\sigma_{\text{overall},1,r}+\sigma_{\text{overall},2,r}\right)},$$

where *σ*_overall,*j*,*r*_ is the intra‐group standard deviation of the *j* ^th^ sample distribution. Assuming the absence of covariance between the susceptibility values in *r* and the reference region (e.g., the regions are disjoint), propagation of uncertainty allows decomposing *σ*_overall,*j*,*r*_ as

(2) $$\begin{array}{c} \sigma_{\text{overall},j,r}^{2}=\sigma_{\text{true},j,r}^{2}+\sigma_{\text{reference},j}^{2}+\sigma_{\text{measure},j,r}^{2}, \end{array}$$

where *σ*_true,*j*,*r*_ is the standard deviation of the true tissue susceptibility in the ROI, which is given by the biological differences between subjects, *σ*_reference,*j*_ is the standard deviation of the true tissue susceptibility in the reference region, and *σ*_measure,*j*,*r*_ is the standard deviation due to the measurement process. The quantity *σ*_measure,*j*,*r*_ can further be decomposed into contributions due to reconstruction artifacts within the ROI, *σ*_artifacts,*j*,*r*_, and within the reference region, *σ*_artifacts,reference,*j*_:

(3) $$\begin{array}{c} \sigma_{\text{measure},j,r}^{2}=\sigma_{\text{artifacts},j,r}^{2}+\sigma_{\text{artifacts},\text{reference},j}^{2}. \end{array}$$

When the region *r* and the reference region are not independent, for example, when whole‐brain referencing is used or if large‐scale artifacts introduce covariance between disjoint regions, the covariance between the two regions must be small for the decomposition to remain valid.

In clinical studies, it is often desirable to use algorithms that detect susceptibility differences between groups or over time with a high absolute effect size, $\left|d_{r}\right|$. To achieve a high absolute effect size, the algorithms need to accurately calculate the absolute susceptibility difference in Equation (1) (determined by the biology), $|\Delta \chi_{r}$|, and both the (biological) variation in the reference region, $\sigma_{\text{reference}}^{2}$, and the variation caused by the measurement process, $\sigma_{\text{measure},j,r}^{2}$, must be small.

In an ideal world, the variation due to the measurement process would be $\sigma_{\text{measure},j,r}^{2}=0$, and the effect size would be determined entirely by biological variability, $\sigma_{\text{overall},j,r}^{2}\approx \sigma_{\text{true},j,r}^{2}+\sigma_{\text{reference},j}^{2}$. However, in the real world, artifacts and noise are unavoidable, for example, due to motion during the data acquisition (Schweser et al. 2016), increasing $\sigma_{\text{overall},j,r}^{2}$ and, consequently, decreasing $\left|d_{r}\right|$. In addition to nonsystematic effects, systematic errors in the QSM pipeline may reduce $\left|d_{r}\right|$, for example, through an inability to accurately capture susceptibility differences, $\Delta \chi_{r}$, between the sample distributions.

## 3 Materials and Methods

### 3.1 Study Design

Real‐world evaluations of QSM have been limited by the lack of a ground truth for in vivo susceptibility quantification (Langkammer et al. 2018). In the present study, we relied on the premise that aging‐related changes in DGM susceptibility are driven by well‐established aging‐related changes in iron concentration (W. Li, Wu et al. 2014). In their 1958 landmark paper, Hallgren and Sourander (1958) (H&S) showed that histochemically determined iron concentrations followed an exponential saturation trajectory with age in most brain regions. The rate of aging‐related iron concentration changes differed between regions, with the putamen and caudate increasing throughout adulthood (see p. 48 therein). In the thalamus, iron concentrations decreased from the fourth decade of life onwards (figure 8 therein) (Hallgren and Sourander 1958). While the reason for these aging‐related changes in brain iron remains poorly understood, H&S's characteristic iron trajectories have been replicated successfully using QSM (W. Li, Wu et al. 2014).

Myelin, which is the major contributor to susceptibility contrast in the brain besides iron (Baxan et al. 2010), appears to undergo its most rapid increase in the first three decades, followed by relative stability and a slow decline thereafter, with myelination in the gray matter (GM) remaining relatively stable (from third to sixth decade; refer to figures 5 and 6 in Dvorak et al. (2021), top left panel). Myelination also remains stable in the thalamus (age range: 18.1–69.0 years) (Cagol et al. 2024)—a region with overall lower iron concentration compared to other DGM regions (Hallgren and Sourander 1958; Langkammer et al. 2012).

Based on this evidence, we presumed that properly functioning QSM pipelines should detect increasing susceptibility *over time* in the GP, putamen, and caudate of healthy adults (*d* > 0) and declining susceptibility in the thalamus (*d* < 0 for age > 35 years) if all other systematically contributing biological factors to tissue susceptibility may be neglected.

We used the effect size of aging‐related changes in susceptibility as a key performance metric to compare QSM pipelines. In this context, the effect size represented the sensitivity toward the detection of aging‐related effects and may be considered as a surrogate metric of general sensitivity of QSM toward over‐time changes or, more generally, any kind of group differences (including cross‐sectional) in the clinical research setting. Based on our considerations in Section 2, we assumed that reconstruction artifacts would always decrease (but never increase) $\left|d_{r}\right|$ compared to the ideal setting and, hence, we considered the pipelines with the highest effect sizes as optimal.

### 3.2 Study Participants

This prospective study enrolled *N* = 25 subjects without neurological disease. Eligible study participants were selected from an institutional database of healthy controls that had participated in previous IRB‐approved studies at our institution and provided their informed consent. The database was filtered concerning the availability of raw k‐space data for a specific gradient‐echo imaging sequence at a specific 3 T MRI instrument and sorted for the first exam date on which the sequence had been applied. To minimize confounding effects from myelin, we restricted enrollment to subjects older than 35 years. Identified subjects were called in for a follow‐up exam in the order of the date of their first exam available in the database, starting with the subjects that had the earliest exam dates to maximize follow‐up time in the resulting cohort.

We recruited another *N* = 5 subjects without neurological disease for a scan‐rescan experiment.

All study procedures were approved by the Institutional Review Board of the University at Buffalo, and all participants (*N* = 30) provided written informed consent in accordance with the Declaration of Helsinki.

### 3.3 Imaging

We used the same MRI scanner (3 T GE Signa Excite HDx 23.0 scanner; General Electric, Milwaukee, WI, USA) for the follow‐up experiment that was also used for the baseline experiment. The scanner used an eight‐channel head‐and‐neck coil. There were no major hardware or software upgrades to the MRI system between the baseline and follow‐up exams. The data for QSM was collected at baseline using a 3D single‐echo spoiled gradient recalled echo (GRE) sequence with first‐order flow compensation in the read and slice directions, a matrix of 512 × 192 × 68 and a nominal resolution of 0.5 × 0.5 × 2 mm^3^ (FOV = 256 × 192 × 128 mm^3^), flip angle = 12°, TE/TR = 22 ms/40 ms, and bandwidth = 13.89 kHz. The use of a single‐echo GRE sequence in this study reflects historical considerations. This sequence was originally implemented to adhere to the recommendations for vein delineation with susceptibility weighted imaging (SWI) (Haacke et al. 2004; Haacke et al. 2015; Liu et al. 2015) including low readout bandwidth and anisotropic voxels. To ensure consistency across longitudinal measurements, we used identical pulse sequence parameters for the prospectively acquired data. Each channel's raw k‐space data was retained for offline image reconstruction. Additionally, the following sequence was acquired for brain segmentation (see Section 3.5; also identical between baseline and follow‐up): fast spoiled gradient‐echo pulse sequence with inversion recovery (IR‐FSPGR) for T_1_‐weighted (T1w) imaging using the following parameters: TE/TI/TR = 2.8 ms/900 ms/5.9 ms, matrix = 256 × 192 × 128, nominal resolution of 1 × 1 × 1 mm^3^ (FOV = 256 × 192 × 192 mm^3^), and flip angle = 10°.

Scan‐rescan subjects received the same GRE sequence four times, interleaved with removal of the subject from the magnet, full repositioning, and recalibration of the MRI system. To capture potential effects due to variability in imaging slab prescription (often present in clinical studies), we ensured that the imaging slabs were not prescribed identically across scans by intentionally introducing small, random oblique angles to the prescription between repetitions. This approach allowed us to evaluate the reproducibility of the measurements under realistic variations in slab orientation.

### 3.4 Processing

#### 3.4.1 Image Reconstruction

The GRE images for QSM were reconstructed offline from the raw k‐space data on a 512 × 512 × 64 spatial matrix using sum‐of‐squares for magnitude images and scalar phase matching for phase images (Hammond et al. 2008). The k‐space data were zero‐padded in the phase‐encoding direction before the processing to achieve an isotropic in‐plane resolution, in line with the manufacturer‐based image reconstruction. Distortions due to imaging gradient nonlinearity were compensated for by unwarping the complex‐valued GRE images using spherical harmonics, as previously described (Polak et al. 2015), following which phase images were unwrapped utilizing a best‐path algorithm (Abdul‐Rahman et al. 2007).

#### 3.4.2 Included Algorithms

##### 3.4.2.1 BFR Algorithms

We included the following widely used and publicly available BFR algorithms: Improved HARmonic (background) PhasE REmovaL using the LAplacian (iHARPERELLA) (W. Li, Avram et al. 2014), Laplacian Boundary Value (LBV) (Zhou et al. 2014), Projection onto Dipole Fields (PDF) (Liu, Khalidov et al. 2011), Regularization Enabled SHARP (RESHARP) (Sun and Wilman 2014), Sophisticated Harmonic Artifact Reduction for Phase (SHARP) (Schweser et al. 2011), and variable‐radius SHARP (V‐SHARP) (Schweser et al. 2011; Li et al. 2011).

##### 3.4.2.2 Inversion Algorithms

The selection of inversion algorithms was primarily guided by the results of the QSM Reconstruction Challenge 2.0 (QSM Challenge 2.0 Organization Committee et al. 2021). We included the top‐ranking inversion algorithms (*N* = 5) from the normalized root mean square error (NRMSE) category, with one algorithm (MEDI) (Liu et al. 2012) from stage 1 and four (FANSI [Milovic et al. 2018], HD‐QSM [Lambert et al. 2022], L1‐QSM [Milovic et al. 2022], Weak Harmonic QSM [WH‐FANSI] [Milovic et al. 2019]) from stage 2 of the challenge (QSM Challenge 2.0 Organization Committee et al. 2021). Additionally, we considered algorithms (*N* = 14) that were publicly available, developed in our lab, or shared with us directly for inclusion following the presentation of preliminary results of this study at the 2023 Annual Meeting of the ISMRM (Salman et al. 2023) (AMP‐PE [S. Huang et al. 2023], DeepQSM [Bollmann et al. 2019], DeepQSM with in‐house generated training data [Jochmann et al. 2020] [DeepQSM*], DirTIK [Karsa et al. 2020; Schweser, Deistung et al. 2013], HEIDI [Schweser et al. 2012], iLSQR [Li et al. 2015], LSQR [Schweser et al. 2012], MEDI+0 [Liu et al. 2018], MEDI+0 without CSF‐regularization [Liu et al. 2018] [MEDI+0*], MATV [Guo et al. 2018], QSMnet+ [Jung et al. 2020], SDI [Schweser, Deistung et al. 2013], STAR [Wei et al. 2015], TKD [Schweser, Deistung et al. 2013; Shmueli et al. 2009], IterTIK [Karsa et al. 2020; Schweser, Deistung et al. 2013], iSWIM [Tang and Liu 2013]). MEDI‐based algorithms (MEDI, MEDI+0, and MEDI+0*) were operated without the spherical mean value (SMV) option.

For details regarding the versions used for each algorithm, please refer to Table S1.

#### 3.4.3 Included Reference Regions

Reference regions were chosen based on frequent use in the literature, past evaluations (Rua et al. 2020; Straub et al. 2017), as well as inclusion in the recent QSM consensus statements (QSM Consensus Organization Committee et al. 2024): whole brain (WB) (X. Li et al. 2019; Schweser et al. 2018; Zivadinov et al. 2018), white matter (WM) (Deistung et al. 2013; Langkammer et al. 2012; Lotfipour et al. 2012; Reichenbach et al. 2015), and cerebrospinal fluid (CSF) in the bilateral ventricles (Deh et al. 2018; Langkammer et al. 2016; W. Li et al. 2011; Lim et al. 2013).

In total, we compared *N* = 378 (6 BFRs × 21 inversions × 3 reference regions) pipelines. We used default parameters for all algorithms or parameters suggested by the original authors in their original publications. If default or publication‐guided parameters resulted in apparent artifacts or results that differed qualitatively from those presented in the original publications, we contacted the corresponding author of the original publication and asked for assistance with the implementation of the algorithm.

#### 3.4.4 Background Field Removal

The initial brain mask (mask 1) was generated by applying a whole‐brain segmentation tool—FSL‐brain extraction tool (BET) (Smith 2002) to the magnitude images. This mask removed air, skull, and other tissues while preserving cortical areas. Following this, a mask of reliable phase values (mask 2) was generated by thresholding the two‐pixel finite difference of the unwrapped phase at an empirically determined value of 2.6 rad. Subsequently, masks 1 and 2 were logically combined, and holes were filled by first dilating the mask and then performing erosion from the outer boundary to obtain mask 3. This resultant mask was used for the BFR step (Figure S1).

We applied each of the above‐mentioned BFR algorithms to the unwrapped phase.

##### 3.4.4.1 SHARP and V‐SHARP

We chose resolution‐ independent high‐pass regularization for the SHARP (Schweser et al. 2011) and V‐SHARP (Schweser et al. 2011; Li et al. 2011) techniques as recommended by Özbay et al. (2017), along with the optimal radius (18 voxels maximum radius) and regularization parameter (=0.0074 mm^−1^) determined in that work.

##### 3.4.4.2 PDF and LBV

PDF (Liu, Khalidov et al. 2011) and LBV (Zhou et al. 2014) were originally developed using transceiver‐phase free field maps from multi‐echo data (personal communication). However, our phase data inherently contained transceiver‐phase contributions. We found that the transceiver phase interfered with the convergence of the PDF method when the default algorithmic parameters were used (30 maximum iterations; 0.1 tolerance). In consultation with one of the authors of the PDF algorithm (Pascal Spincemaille, Weill Cornell Medicine), we optimized the parameters (1500 maximum iterations; 0.1 tolerance) for our dataset and added fourth‐order 3D polynomial fitting after PDF to suppress nonharmonic transceiver‐phase contributions. The polynomial fitting was also applied after LBV with the same parameters as applied after PDF.

##### 3.4.4.3 RESHARP

Since the regularization parameter specified in the original publication (*λ* = 5·10^−3^) resulted in obvious over‐regularization, we consulted with two of the original authors (Alan Wilman, University of Alberta; Hongfu Sun, University of Newcastle), who advised using *λ* = 1·10^−4^. Additionally, as per the authors' request, we applied polynomial fitting with the same parameters as used for LBV and PDF to the RESHARP output.

#### 3.4.5 Dipole Inversion

**FIGURE 1:**
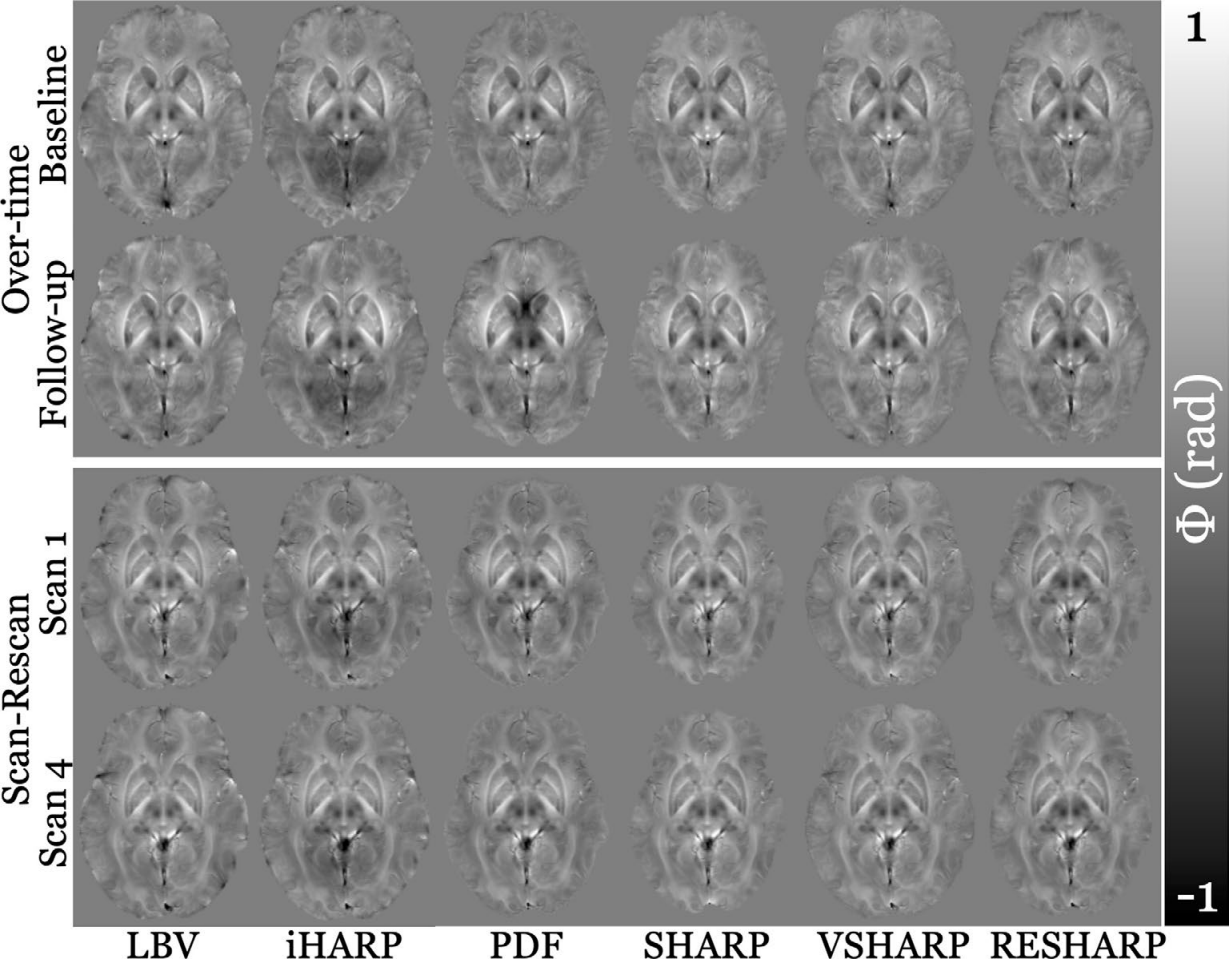
Background field‐corrected field maps in the same slice of the template as shown in Figure S3. The top panel displays maps from a representative subject in the longitudinal cohort (female, age at baseline = 55 years, follow‐up = 65 years), while the bottom panel shows maps from the first and last scans of a subject in the reproducibility cohort (male, age = 26 years). Each column corresponds to a different BFR algorithm.

Each BFR algorithm created an eroded mask specific to the algorithm (see BFR‐specific erosions in Figure 1) and subject scan (mask 4). We logically combined masks 2 and 4 to create a mask for the dipole inversion step, mask 5 (Figure S1).

We applied each of the above‐mentioned inversion algorithms to each of the BF‐corrected field maps. Their parameters can be found in the provided code repository (see Section 3.4.8). For all algorithms, BFR output maps were rescaled to the physical units expected by each inversion algorithm before the algorithm was applied.

We requested assistance from the authors of WH‐FANSI, L1‐QSM, HD‐QSM (Carlos Milovic, Pontificia Universidad Católica de Valparaíso), and AMP‐PE (Shuai Huang, Emory University) due to unsatisfactory results with their algorithms' default parameters. For AMP‐PE, the author recommended using high‐order (“db1”) instead of the default low‐order (“db2”) wavelet bases. For WH‐FANSI, we performed L‐curve optimization under the guidance of one of the authors. For L1‐QSM and HD‐QSM, although no exact parameters were suggested, the information provided by the authors allowed us to compute satisfactory susceptibility maps from these algorithms.

All computed susceptibility maps were multiplied with mask 6 (Figure S1), obtained by filling holes (as above) in mask 5 before reporting the findings.

##### 3.4.5.1 Deep Learning Algorithms

The included deep learning algorithms had specific requirements for the imaging slab orientation (all strictly axial), voxel aspect ratio (all isotropic), and voxel edge length (DeepQSM and QSMnet+: 1 mm; DeepQSM*: 0.5 mm). We applied the following pre‐ and post‐processing steps to the BF‐corrected field maps for these algorithms:

First, the BF‐corrected field maps were spatially resampled using FreeSurfer's mri_convert tool with cubic interpolation to the expected isotropic resolutions. Subsequently, the isotropic maps were rotated to a strictly axial orientation using an in‐house developed and thoroughly validated rotation tool based on FSL‐FMRIB's Linear Image Registration Tool (FLIRT) (Jenkinson et al. 2002) with trilinear interpolation using header information about the orientation of the imaging slab. We then applied the deep learning models and rotated the resulting susceptibility maps to their original orientation (native subject space). This processing was followed by resampling back to the original image resolution.

#### 3.4.6 Total Number of Reconstructions

Considering all BFR and inversion algorithms mentioned above, the total number of computations required to reconstruct the full dataset (longitudinal [*N* = 50] and reproducibility [*N* = 20]) was estimated to be 8820 (70 exams × 6 BFR algorithms × 21 inversion algorithms).

#### 3.4.7 Dipole Inversion Computation Time

We measured the average computation time of the inversion algorithms by applying each algorithm successively to all 420 BF‐corrected field maps (70 exams × 6 BFR algorithms × 1 inversion). No graphics processing units were allocated to ensure comparability across algorithms.

#### 3.4.8 Scientific Rigor

To ensure optimal method implementation, all processing for QSM relied on code provided by the original algorithm developers, unless explicitly stated. To ensure high scientific rigor and avoid unintentional bias toward our in‐house developed algorithms, we performed all processing fully automated and reproducible using containerized computing on a high‐performance cluster with four CPUs (Intel Xeon Gold 6330) and 20 GB of memory allocated to each job. We used an in‐house developed job scheduler for multistep pipelines (pi4s; https://gitlab.com/R01NS114227/pi4s). All algorithms were executed in a custom Singularity (Kurtzer et al. 2017) (Apptainer) container generated with Neurodocker (https://www.repronim.org/neurodocker/), ensuring an identical computational environment for all algorithms. The container included Matlab (version 2018b; The MathWorks, Natick, MA, USA), FreeSurfer (v6.0.0), FSL (v5.0.8), and Advanced Normalization Tools (ANTs; v2.0.0). For the deep learning methods, we used a containerized version of Python (v3.6.9, with Tensorflow v2.1.0). All computer code has been made available at https://doi.org/10.5281/zenodo.11077423 (Salman 2024).

### 3.5 Susceptibility Analysis

We calculated mean susceptibility values in four DGM regions (GP, caudate, putamen, and thalamus). The subject‐specific regional segmentations were computed fully automatically using an in‐house developed bi‐parametric atlas‐based segmentation technique (Hanspach et al. 2017), which relied on both susceptibility and T1w imaging contrasts.

We preprocessed the susceptibility maps and T1w images for multi‐contrast template reconstruction as described earlier (Hanspach et al. 2017). For this, we used susceptibility maps calculated with SDI (Schweser, Deistung et al. 2013) because it is one of the simplest QSM algorithms yielding susceptibility maps with relatively high visual quality, and it does not involve spatial regularization that could bias anatomical contrast in the final template. Third, a trained image analyst (A.A.) created atlas labels by manually outlining the four bilateral subcortical regions as well as the CSF (bilateral ventricles; for referencing) in the template space using MRIcron software (v1.0.20190902) using both QSM and T1w contrast. We used SynthSeg (Billot et al. 2023) on the T1w template to obtain WM labels for referencing. Fourth, we propagated all atlas labels to the native subject spaces utilizing bi‐modal warp field computations from ANTs (Avants et al. 2009). Template‐level and all propagated DGM labels were carefully inspected by trained analysts (F.Sa., 5 years of neuroimaging experience; F.Sc., 15 years; N.B., 20 years) and corrected where needed. WM labels were adjusted to accommodate the maximum erosion performed across all BFR algorithms (always the SHARP mask), which ensured consistency in voxel coverage of WM average values across all pipelines. The atlas‐based CSF labels were manually corrected (by F.Sa.) in each subject's native space using susceptibility contrast reconstructed with SDI to exclude that tissues within or at the boundaries of the ventricles were included in the label segment (e.g., veins, calcification, or choroid plexus). Finally, we applied all subject‐space labels to all reconstructed susceptibility maps and calculated regional mean susceptibility values in each hemisphere using FSLstats (Jenkinson et al. 2012) (fully scripted). Subsequently, we averaged the regional mean values for both hemispheres to obtain an average regional mean susceptibility value and referenced the values to each of the three reference regions (Figure S2).

### 3.6 Statistical Analysis

All statistical analyses were conducted using Statistical Product and Service Solutions (SPSS; version 28; IBM, Armonk, NY, USA) and Microsoft Excel (v16.8, Microsoft Corporation, Redmond, WA, USA). Bilateral ROI measures were averaged between both hemispheres and tested for normality using Q‐Q plots and the Shapiro–Wilk test.

#### 3.6.1 Reproducibility Error

We used the scan‐rescan data to quantify each pipeline's reproducibility error. Due to the short interval between the reproducibility scans, it was assumed that $\sigma_{\text{true},r}^{2}{+\sigma }_{\text{reference}}^{2}=0$ in Equation (2) and, hence, $\sigma_{\text{overall},r}=\sigma_{\text{measure},r}$, that is, the *observed* scan‐rescan variation is an estimate of the measurement‐related variation.

**FIGURE 2:**
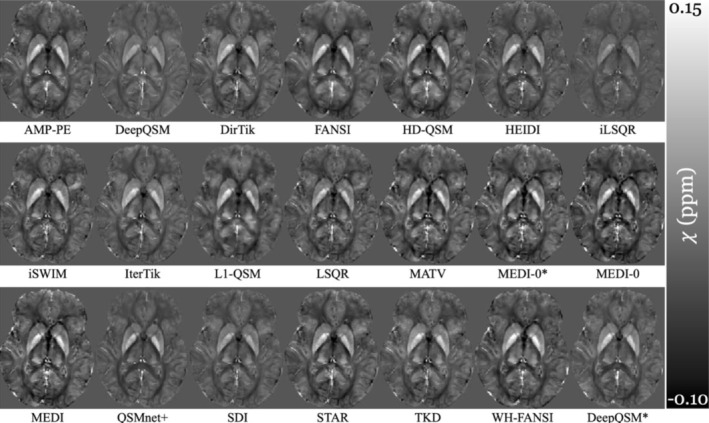
Susceptibility maps in native space of a representative healthy subject (26 years old male) from different inversion algorithms using LBV as the BFR.

We assessed a pipeline p's reproducibility in ROI r through a reproducibility error metric, calculated from the subject‐average scan‐rescan standard deviation, ${\hat{\sigma }}_{\text{overall},\mathrm{rp}}$. Since dipole inversion algorithms have a propensity to underestimate magnetic susceptibility (de Rochefort et al. 2010; Kressler et al. 2010; Liu, Surapaneni et al. 2011; Schweser et al. 2011; Zhu et al. 2022) (cf. Figure 2), which would affect ${\hat{\sigma }}_{\text{overall},\mathrm{rp}}$ differently for each algorithm and, hence, would hamper the comparison of the metric across pipelines, we normalized each pipeline's ${\hat{\sigma }}_{\text{overall},\mathrm{rp}}$ using the average of the susceptibility across all four investigated DGM regions and all five subjects:

(4) $$\begin{array}{c} \rho_{\mathrm{rp}}=\frac{{\hat{\sigma }}_{\text{overall},\mathrm{rp}}}{\frac{1}{5}\cdot \sum_{n=1}^{5}\left(\frac{1}{4}∙\sum_{s=1}^{4}\chi_{\mathrm{spn}}\right)}\cdot 100, \end{array}$$

where $\chi_{\mathrm{spn}}$ is the average susceptibility in region s calculated with pipeline p in subject n. The resulting reproducibility error metric, *ρ*_*rp*_, denotes the amount of scan‐rescan variation as a fraction of the average DGM susceptibility (in percent).

For visualization purposes, we also calculated the voxel‐wise reproducibility error using susceptibility maps co‐registered to the QSM‐T1w template as:

(5) $$\begin{array}{c} \rho \prime_{\mathrm{pi}}=\frac{{\hat{\sigma }}_{\text{overall},\mathrm{ri}}}{\frac{1}{5}\cdot \sum_{n=1}^{5}\left(\frac{1}{4}∙\sum_{s=1}^{4}\chi_{\mathrm{spn}}\right)}\cdot 100, \end{array}$$

where *i* denotes the *i* ^th^ voxel.

#### 3.6.2 Correlation of Putative Over‐Time Iron Changes With Susceptibility Changes

For each subject, we calculated putative regional iron concentrations from the regional age‐dependency equations provided by H&S (Hallgren and Sourander 1958) using age at baseline and follow‐up time points. Following this, we calculated the putative baseline‐to‐follow‐up change in group‐average iron concentrations for the enrolled cohort. Since the work by H&S did not provide an analytical trajectory for iron in the thalamus, we extracted the data from figure 8 in their publication (Hallgren and Sourander 1958) using WebPlotDigitizer (v3.9; Ankit Rohatgi, Austin, TX, USA; http://arohatgi.info/WebPlotDigitizer/) and fitted a linear function to the data for ages 35 years and older.

We determined how well over‐time susceptibility changes correlated with putative H&S iron changes by converting each subject's regional susceptibility values at follow‐up and baseline to iron concentrations using an experimentally determined conversion factor specific to the GM (13.2 ppb/mg iron/100 g‐wet‐weight at 36.5°C) (Langkammer et al. 2012). We calculated the Pearson correlation between putative H&S iron changes and the susceptibility‐derived iron concentrations. If Pearson correlation reached significance (*p* < 0.05), we performed a linear least squares regression and recorded the slope value, which was expected to equal 1 if the iron concentrations derived from observed susceptibility changes quantitatively matched H&S putative iron changes. Finally, *R* ^2^ values were classified using Cohen's 1992 guidelines: ≤ 0.12 indicates low effect size, 0.13–0.25 indicates medium, and ≥ 0.26 indicates high effect size (Cohen 1992). Additionally, we repeated the analysis for iron values individually at baseline and follow‐up time points.

#### 3.6.3 Sensitivity

The practical relevance of scan‐rescan reproducibility is limited without also considering the ability to detect change. For example, a pipeline that would output the exact same susceptibility map independent of the input phase images would have perfect reproducibility (zero error or variation) but would not detect any changes in susceptibility over time or between individuals or groups (zero sensitivity). We used the absolute effect size, $\left|d_{r}\right|$, according to Equation (1), to assess the ability of a pipeline to detect regional susceptibility differences, referred to as the pipeline's “sensitivity” in the following. Pipelines that detected over‐time changes inconsistent with H&S were masked out in visualizations of the metric (see Section 3.6.8). These exclusions aimed to differentiate between pipelines detecting over‐time susceptibility changes in line with H&S and not in line with H&S.

For each pipeline p, we used the group‐average over‐time change in susceptibility between baseline and follow‐up acquisitions (*N* = 25) in region r as the nominator, $\Delta \chi_{\mathrm{rp}}$, in Equation (1), and the subject‐average standard deviation of the scan‐rescan acquisitions (*N* = 5), $\sigma_{\text{measure},\mathrm{rp}}={\hat{\sigma }}_{\text{overall},\mathrm{rp}}$, as the denominator:

(6) $$\begin{array}{c} {\hat{d}}_{\mathrm{rp}}=\left|\frac{\Delta \chi_{\mathrm{rp}}}{{\hat{\sigma }}_{\text{measure},\mathrm{rp}}}\right|. \end{array}$$

#### 3.6.4 Global Performance Metrics

To capture and compare the average performance of pipelines across all DGM regions, we combined each pipeline's regional performance metrics, $\rho_{\mathrm{rp}}$ and ${\hat{d}}_{\mathrm{rp}}$, into a composite, global performance metric, *P*. We computed *P* by averaging the regional findings, as

(7) $$\begin{array}{c} P_{\rho }=\frac{\sum_{r}^{4}\rho_{\mathrm{rp}}}{4}\;\text{and}\;P_{d}=\frac{\sum_{r}^{4}d_{\mathrm{rp}}}{4}, \end{array}$$

respectively.

#### 3.6.5 Scientific Rigor

We conducted all analyses in a blinded manner, with unblinded metrics only being inspected collectively once each metric (reproducibility error, over‐time change, and sensitivity) was available for all algorithms.

Extensive quality control measures were implemented, involving the visualization of randomly selected resultant subject maps from various BFR and inversion algorithms. The quality control process extended beyond the assessment of susceptibility maps alone; meticulous checks were performed on regional susceptibility values to ensure accuracy.

#### 3.6.6 Comparative Analysis

For the BFR and inversion steps, we assessed the algorithmic performance using three metrics: normalized reproducibility error, normalized over‐time susceptibility change, and sensitivity. For each of the six BFR algorithms, we calculated the median of each metric across all 21 inversion algorithms. For each of the 21 inversion algorithms, we calculated the median of each metric across all six BFR algorithms. The algorithm with the best median value for each metric (lowest for reproducibility error; highest for over‐time change and sensitivity) was considered the best‐performing algorithm for BFR and dipole inversion, respectively.

To test the statistical significance of the performance differences, we performed paired *t*‐tests. For example, to compare the performance between two BFR algorithms, we performed paired *t*‐tests across the metric values from all 21 inversion algorithms. Conversely, to compare two inversion algorithms, we performed paired *t*‐tests across the metric values from all BFR algorithms. The complete analysis was conducted separately for each reference region (e.g., WB, WM, and CSF).

All *p*‐values were corrected for multiple comparisons using the false discovery rate method (denoted as *q*‐values; Benjamini and Hochberg 1995) and results were considered statistically significant when *q* < 0.05; uncorrected *p* < 0.05 was considered a trend.

#### 3.6.7 Percentile‐Based Classification

To evaluate the distribution across pipelines, we computed the 33rd and 66th percentiles for the reproducibility error and sensitivity metrics using the *prctile* function in MATLAB. The respective metric value of pipelines in the 33rd percentile was considered *low*, in the 66th percentile as *medium*, and above the 66th percentile as *high*.

#### 3.6.8 Visualization

We visualized all pipeline and region‐specific metrics as heat maps using the seaborn library (v0.12.2; Waskom 2021). To facilitate visual inspection, we decided to mask (gray boxes in regional over‐time and sensitivity; translucent in global sensitivity) any observations that deviated from the aging‐related H&S‐based over‐time changes.

#### 3.6.9 Visual Evaluation of Top‐Performing Pipelines

We conducted a qualitative rater‐based comparison of the susceptibility maps in the 95th percentile of the global sensitivity metric using 10 subjects randomly selected from both cohorts (longitudinal and reproducibility) and three independent raters with 3–9 years of experience in brain QSM research (J.A.R., T.J., and J.C.). For increased scientific rigor, we included one rater (J.C.) who had previously worked at a different institution (Weill Cornell Medicine) and had limited prior exposure to our in‐house developed algorithms and the appearance of the resulting susceptibility maps. The other two raters obtained the majority of their experience with QSM in our lab (J.A.R. and T.J.). Raters were blinded to the pipelines used. The evaluation was conducted using ITK‐SNAP (Yushkevich et al. 2006), with all 95th percentile maps presented side by side with the same preset contrast setting. For each subject, raters were instructed to rank the susceptibility maps using the following criteria: presence of artifacts (e.g., streaking); image homogeneity; visibility and differentiation of fine anatomical structures; natural appearance of the image. Regional intensity differences were not considered for ranking. One of the raters (J.A.R.) was selected for an intra‐rater evaluation after 2 weeks of the initial ranking. The susceptibility maps were shuffled, and the same rating procedure was repeated.

Fleiss' kappa (Fleiss 1971) (*κ*) was calculated to assess the inter‐rater agreement and assessed using the classification scale guidelines from Altman (1991). Additionally, the intra‐class correlation coefficient was assessed in accordance with established guidelines from the literature (Koo and Li 2016).

## 4 Results

### 4.1 Participants

Two subjects had to be excluded from the study due to data integrity issues, resulting in *N* = 23 subjects for the longitudinal analysis. The average age of included subjects was 57 ± 9 (39–73) years at the time of the baseline scan with a female:male ratio of 19:4. The median time between baseline and follow‐up scans was 10.0 years [11.5–13 interquartile range (IQR)].

### 4.2 Computation and Quality Control

Considering the above‐mentioned exclusions, a total of 8316 susceptibility maps were reconstructed.

The dipole inversion computation times were derived using 396, instead of 420, BF‐corrected field maps (2 subjects [4 scans] excluded; see the formula in Section 3.4.7). Computing times ranged from 22 s (TKD) to 46 h (WH‐FANSI) per scan (Figure S4).

Reconstruction failed with DeepQSM for one follow‐up scan (all BFR algorithms; no output produced). Additionally, one scan at baseline, computed using LBV + FANSI, was excluded due to failed reconstruction (monochrome output; no brain structures visible). These baseline and follow‐up scans were excluded from the subject‐wise H&S‐based correlations performed to assess over‐time changes. Exclusion did not alter the median or IQR of the time between the baseline and follow‐up scans used to investigate the longitudinal susceptibility changes in this study.

Figure 1 shows the BF‐corrected field maps generated by all BFR algorithms, while Figure 2 presents the susceptibility maps reconstructed using LBV as the BFR with all inversion algorithms. No major artifacts were observed in either the BF‐corrected field maps (Figure 1) or the susceptibility maps (Figure 2). As expected, varying BFR‐specific erosion was evident at the brain's surface in Figure 1, while the susceptibility maps in Figure 2 differed primarily in overall intensity (related to systematic underestimation) and edge definition (smoothing), two well‐characterized limitations of many dipole inversion algorithms (Langkammer et al. 2018; QSM Challenge 2.0 Organization Committee et al. 2021).

### 4.3 Reproducibility

#### 4.3.1 SHARP‐Based BFR Algorithms Demonstrated the Best Reproducibility

**FIGURE 3:**
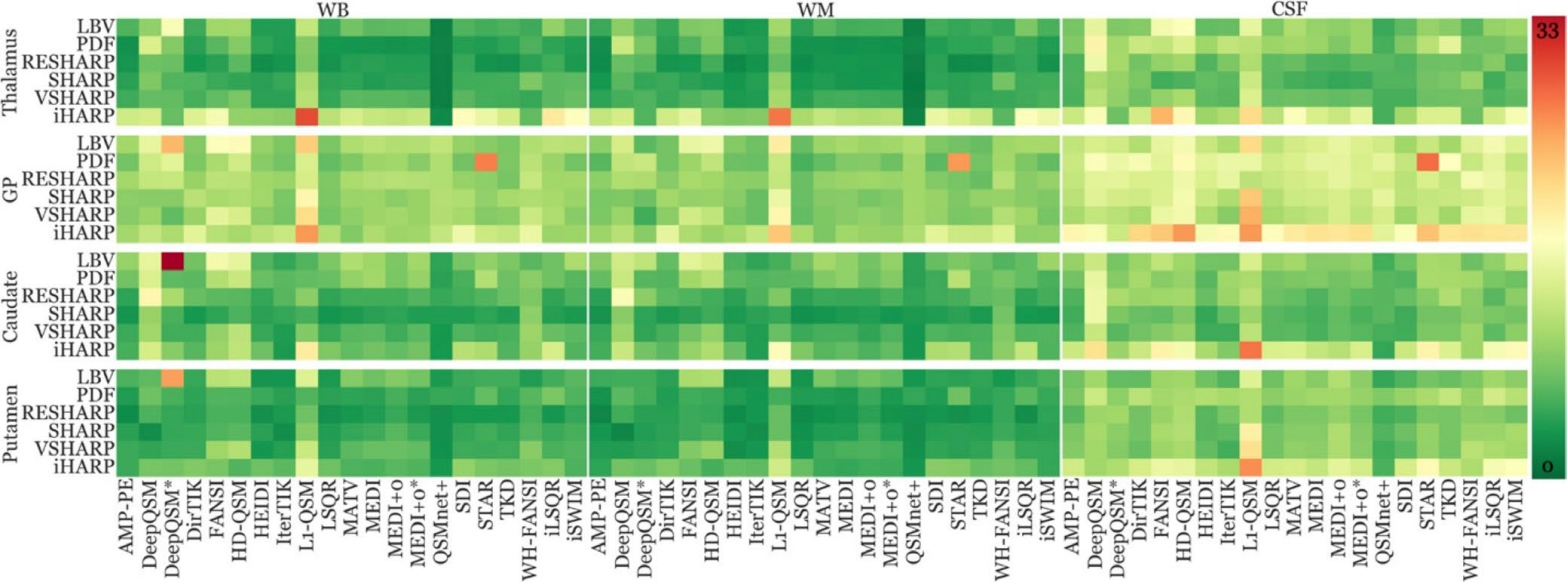
Normalized reproducibility (scan‐rescan variation) findings of each pipeline and region. Lower values (green) represent higher reproducibility (lower variation), and vice versa for red. Each group of three horizontal panels combines the results of one specific DGM region that is denoted at the left‐hand side of the panel. The three horizontal panels of each region summarize results using one specific reference region, which is listed at the top. Within each panel, each row corresponds to a specific BFR algorithm (listed on the left‐hand side) while each column represents an inversion algorithm (listed at the bottom). See Figures S5–S7 for an annotated version of the figure with individual pipeline metrics.

Figure 3 summarizes reproducibility error metrics for each pipeline and region. The choice of the BFR algorithm had a systematic effect on the reproducibility, with some BFR algorithms performing systematically better than others, independent of the dipole inversion algorithm.

Irrespective of the inversion algorithm and reference region, SHARP‐based BFR algorithms stood out for their high reproducibility (low reproducibility error) in all anatomical regions except for the GP.

Using either WB or WM referencing, SHARP portrayed the lowest reproducibility error across all BFR algorithms, independent of the inversion algorithm (Table S2; SHARP's median reproducibility error with WB referencing = 5.85 [0.78 IQR], WM = 5.08 [0.91]); hence, it was statistically compared to all other BFR algorithms using the same referencing method. All BFR algorithms, except RESHARP (*q* = 0.50), displayed significantly higher reproducibility errors than SHARP (*q* ≤ 0.03). Moreover, 18 out of 21 (86%) inversion algorithms achieved their lowest reproducibility error with either SHARP or RESHARP (WB = 5.90 [1.58 IQR], WM = 5.15 [1.29]), with exceptions being WH‐FANSI, DeepQSM, and L1‐QSM, with both BFR algorithms.

For comparison of CSF‐referenced pipelines, we used V‐SHARP as the reference algorithm because it exhibited the lowest reproducibility error independent of the inversion algorithm (median = 8.29 [1.66]). Except for SHARP and RESHARP (*q* ≥ 0.60), all other BFR algorithms exhibited significantly higher reproducibility errors compared to V‐SHARP (*q* ≤ 0.002).

#### 4.3.2 Inversion With QSMnet+ Demonstrated the Best Reproducibility

In all regions except the GP, QSMnet+ consistently demonstrated the lowest reproducibility error among all inversion algorithms, regardless of the selected BFR algorithm or reference region.

#### 4.3.3 WM Referencing Resulted in the Highest and CSF Referencing in the Lowest Reproducibility

The median normalized reproducibility error across all pipelines and regions was 5.90 [4.25 IQR] for WM referencing, 6.65 [4.82] for WB referencing, and 9.73 [5.47] for CSF‐referencing. Reproducibility was significantly better when using WB (32% lower reproducibility error, *q* = 0.03; paired *t*‐test) or WM referencing (39% lower reproducibility error, *q* = 0.01), compared to CSF‐referencing. No significant difference was observed between WB and WM reproducibility (*q* = 0.07; paired *t*‐test).

Changing the reference region from WB to WM reduced the reproducibility error between 8% (caudate) and 15% (thalamus). Changing from WB to CSF increased the reproducibility error between 29% (GP) and 102% (putamen). Figure S8 provides a quantitative overview of the impact of the choice of the reference region on reproducibility.

#### 4.3.4 GP Presented With the Worst Reproducibility Across all Regions

**FIGURE 4:**
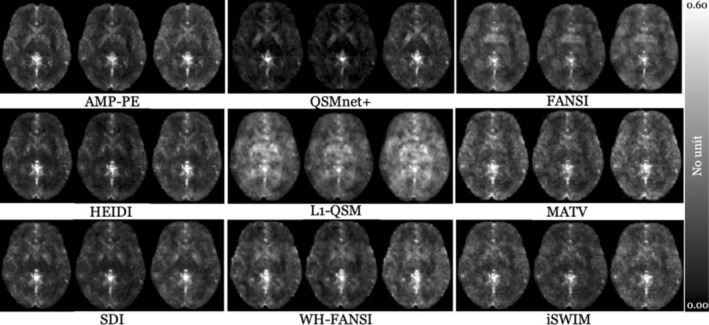
Voxel‐wise normalized reproducibility (*ρ*_*rj*_) for V‐SHARP with selected inversion algorithms covering a wide range of reproducibility values in the same slice of the template as shown in Figure S3. From left to right, susceptibility maps were referenced to WB, WM, and the CSF, respectively.

**FIGURE 5:**
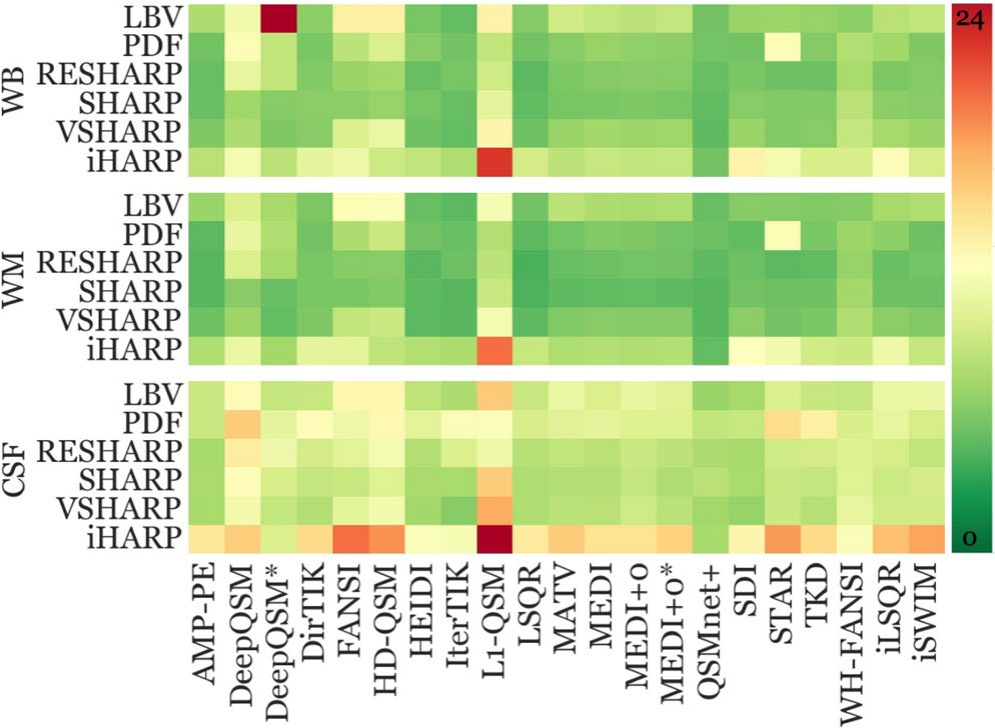
Global performance reproducibility (scan‐rescan variation) metric according to Equation (7) (left). The color‐coding and the arrangement of BFR and inversion algorithms mirrors that of Figure 3. In this figure, each panel represents a specific reference region (listed on the left‐hand side). See Figure S10 for an annotated version of this figure.

Figure 4 illustrates the effect of the reference region choice on voxel‐wise reproducibility errors for selected inversion algorithms. We chose V‐SHARP as a representative BFR algorithm due to the wide range of reproducibility error values observed with pipelines using this algorithm (see Figure 5). Voxels in the GP region consistently demonstrated the highest reproducibility error compared to other regions (brighter voxels in Figure 4; *q* < 0.001), in line with the regional analysis (Figure 3).

#### 4.3.5 Reproducibility Was Similar or Slightly Lower Compared to Previous Investigations

Scan‐rescan variability was comparable to or slightly lower (Figure S9) compared to previous investigations (Feng et al. 2018; Rua et al. 2020; Santin et al. 2017) that employed similar pipelines. As anticipated, reproducibility was generally lower compared to studies using 7 T MRI (Rua et al. 2020).

#### 4.3.6 Global Reproducibility

Figure 5 summarizes the data in Figure 3 using the global region‐averaged reproducibility error metric (Equation 7). The global metrics largely mirrored the regional analyses (Figure 3). Global reproducibility error varied from 4.0 (SHARP or RESHARP; LSQR; WM) to 23.8 (LBV; DeepQSM*; WB).

#### 4.3.7 Highest Reproducibility With QSMnet+ (WB‐Referenced), LSQR (WM), and SDI (CSF) Inversion

With WB referencing, QSMnet+ was used as a benchmark for comparison with other inversion algorithms because this algorithm demonstrated the lowest median reproducibility error across all BFR algorithms (5.10 [0.49 IQR]). Independent of the BFR, 50% of the inversion algorithms demonstrated significantly higher reproducibility errors than QSMnet+ (*q* ≤ 0.042; L1‐QSM: median = 11.61 [3.53], DeepQSM: 10.80 [2.93], HD‐QSM: 9.40 [2.82], FANSI: 9.05 [3.50], WH‐FANSI: 8.04 [1.13], MEDI: 6.93 [2.05], MEDI+0: 6.70 [1.71], MEDI+0*: 6.58 [2.14], iSWIM: 6.48 [2.10], MATV: 6.46 [2.13]). Reproducibility error differences of the remaining inversion algorithms relative to QSMnet+ with WB referencing did not reach significance (*q* ≥ 0.06).

With WM referencing, LSQR's reproducibility was observed to be the best, independent of the BFR (4.65 [0.97 IQR]). Significantly higher reproducibility error compared to LSQR (*q* ≤ 0.04) was observed with L1‐QSM (10.08 [2.91]), DeepQSM (9.75 [2.82]), FANSI (7.99 [3.36]), WH‐FANSI (7.02 [0.81]), iLSQR (6.29 [1.57]), DirTIK (5.64 [0.24]), and TKD (5.61 [0.48]). The remaining inversion algorithms had comparable reproducibility errors to LSQR (*q* ≥ 0.06).

For CSF‐referencing, SDI exhibited the lowest median reproducibility error (7.32 [1.14 IQR]), independent of the BFR. Compared to SDI, all other algorithms showed significantly higher reproducibility errors (*q* ≤ 0.05), except for IterTIK (8.71 [IQR: 3.56]) and QSMnet+ (7.47 [IQR: 0.73]; *q* ≥ 0.27).

### 4.4 Over‐Time Changes

For the sake of conciseness, we focused the presentation of results on WB‐referenced findings (Figures S11 and S12) because of the superior performance of this reference region over CSF in the previous analyses and similar performance compared to WM referencing (Figures S13 and S14 for WM; Figures S15 and S16 for CSF‐referenced findings).

#### 4.4.1 Differences Between Pipelines in Detected Over‐Time Changes Are Not Related to Systematic Underestimation

Detected over‐time changes varied by up to two orders of magnitude between pipelines, with changes ranging from −0.05 to −19 ppb in the thalamus, +3.1 to +34 ppb in putamen, +1.2 to +40 ppb in caudate, and +0.18 to +42 ppb in GP. To compensate for systematic underestimation, we normalized the over‐time change values by using the average across all DGM regions under investigation. This reduced some outliers (such as for DeepQSM) but did not reduce the overall variation in detected changes: −1 to −304 in thalamus, +22 to +459 in caudate, +35 to +459 in putamen, and +2 to +481 in GP.

#### 4.4.2 High Correlation With Putative Over‐Time Iron Changes, but Overestimation

The group‐average over‐time changes in iron concentrations predicted from the H&S data were (in increasing order): −0.347 of fresh tissue weight (mg/100 g) in thalamus (Figure S17; *c*_Fe_(age [y]) = −0.0358 · age [y] + 6.9102), +0.101 mg/100 g in GP, +0.237 mg/100 g in caudate, and + 0.512 mg/100 g in putamen.

**FIGURE 6:**
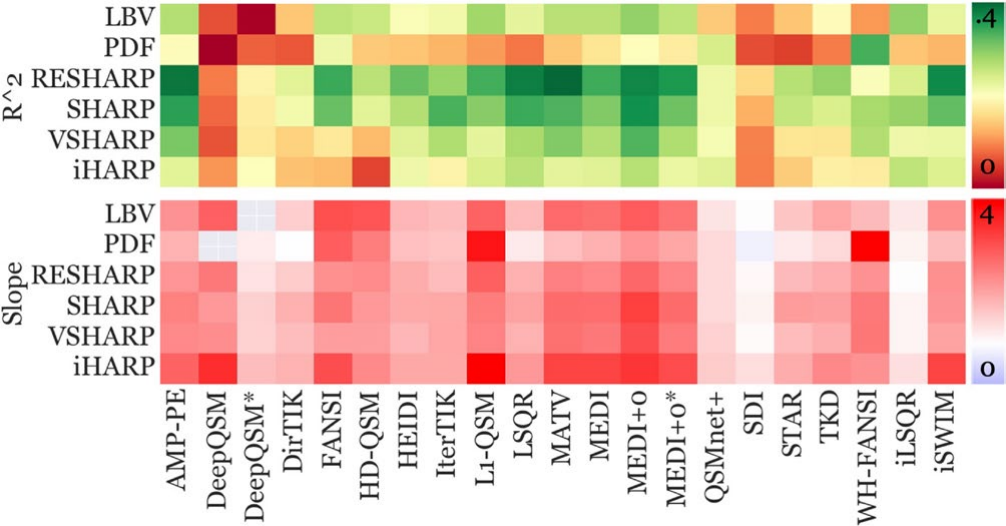
Correlation between observed DGM over‐time susceptibility changes and putative iron changes. The coefficients of determination, *R* ^2^, are displayed at the top, while the slope (white box = 1) is depicted at the bottom. Pipelines within the slope plot that exhibited nonsignificant Pearson correlation (*p* > 0.05) were excluded (gray boxes). See Figure S19 for an annotated version of this figure.

Figure 6 depicts the coefficients of determination, *R* ^2^ (top block), for detected over‐time susceptibility changes in the DGM (WB‐referenced) and the predicted iron changes according to H&S's region‐dependent equations. Additionally, the plot shows the associated fitting slopes (bottom block) for each pipeline, indicating whether the pipeline overestimated (> 1) or underestimated (< 1) the susceptibility changes compared to the predicted over‐time changes. All but two pipelines demonstrated a significant linear correlation (*p* < 0.05, *R* ^2^ threshold = 0.02). Out of the remaining 124 pipelines, 71 pipelines (57%) overestimated putative iron concentrations by at least a factor of two. Only DirTIK with PDF (slope = 1.04), SDI with V‐SHARP (1.12), RESHARP (1.13), LBV (1.07) and PDF (0.87), and iLSQR with RESHARP (1.07) estimated over‐time changes close to the putative iron changes (slope between 0.9 and 1.1).

Predicted iron changes explained between *R* ^2^ = 1% (PDF + DeepQSM and LBV + DeepQSM*) and 39% (RESHARP + MATV) of the variation in susceptibility values. Nineteen pipelines exhibited a low coefficient of variation (*R* ^2^ ≤ 0.12), 63 exhibited medium correlations (≥ 0.13 and ≤ 0.25), while the rest (44) showed high correlations (≥ 0.26).

Figure S18 visualizes the cross‐sectional correlations between observed QSM‐based and predicted iron at baseline and follow‐up timepoints, respectively. Iron values at baseline consistently showed a high correlation (*R* ^2^ ≥ 0.76) for all but one pipeline (*N* = 125/126; 99.2%). Sixty‐two of 126 pipelines (49%) estimated iron values close to the predicted ones (slope between 0.9 and 1.1), while the remaining pipelines either overestimated (maximum of +34%: DeepQSM with LBV) or underestimated (−68%: DeepQSM* with LBV) iron values. Comparable results were observed for the follow‐up timepoint as well.

#### 4.4.3 Artifacts Are Responsible for Over‐Time Changes Inconsistent With H&S Predictions

**FIGURE 7:**
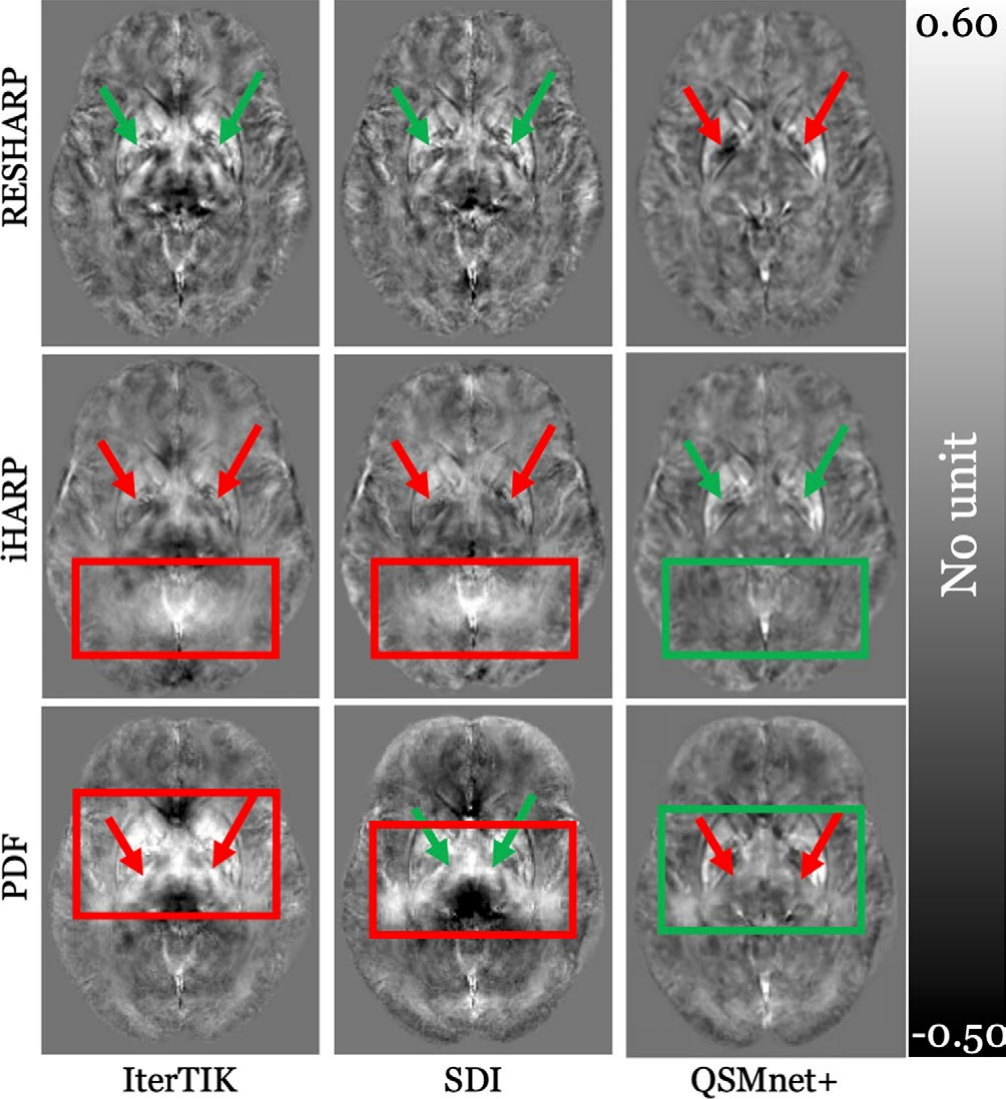
Shown above are the WB‐referenced group‐average susceptibility difference (follow‐up—baseline) maps. *Y*‐axis shows the BFR, while *x*‐axis displays the inversion algorithm used. Difference maps are portrayed in the same slice of the template as shown in Figure S3, and were DGM‐normalized. Color bar was set accordingly (−0.5 to 0.6) to ensure clear visualization of regions and artifacts. Arrows point at the bilateral GP region within RESHARP and iHARP maps, while, thalamic region within the PDF maps, indicating the over‐time susceptibility change detected, with green being consistent with increasing iron concentration, and red being inconsistent. Red boxes highlight a region affected by artifacts in iterTik and SDI. Artifacts were absent (green box) in QSMnet+.

To examine inconsistencies in voxel‐wise over‐time changes, particularly those deviating from H&S‐based predictions (most of PDF‐ and iHARP‐based pipelines), we conducted a post hoc analysis across all pipelines, depicted in Figure 7. Upon scrutinizing these maps in axial, sagittal, and coronal views, we did not find any specific reason for the observed decline in GP susceptibility with iHARP. Conversely, increasing thalamic susceptibility with PDF may possibly have been caused by long‐ranging hyper‐intense artifacts colocating with the DGM. Notably, these artifacts were effectively, though not entirely, suppressed only by QSMnet+ (Figure 7; the red box in the left [IterTIK] and middle [SDI] columns turns green in the rightmost [QSMnet+] column for the middle [iHARP] and last [PDF] rows).

The regional over‐time changes detected with PDF‐based pipelines in the absence of artifacts over the DGM regions (Figure 7—last row, middle column; red box, smaller and lower positioned compared to the left column) were comparable to findings from other BFR algorithms. For example, changes in the putamen were 153 ppb with PDF + SDI compared to 101 ppb with RESHARP + SDI, and 250 ppb with PDF + QSMnet+ compared to 246 ppb with RESHARP + QSMnet+.

In contrast to the above‐discussed PDF‐based susceptibility maps without artifacts colocating with the DGM, PDF‐based pipelines with evident artifacts in the DGM (Figure 7, last row, left column; larger red box compared to the middle column in last row) resulted in amplified regional over‐time changes. For instance, the putamen exhibited a 332 ppb change with PDF + IterTik, which was substantially higher compared to the next largest change observed with SHARP + IterTik (138 ppb).

No prominent artifacts were observed in maps from LBV and SHARP‐based BFR algorithms (select RESHARP‐based inversion maps displayed in first row in Figure 7). However, regardless of the choice of BFR algorithm, the DeepQSM inversion algorithm consistently detected H&S‐inconsistent changes in the thalamus over time.

#### 4.4.4 Differences in Detected Over‐Time Changes Between BFR and Inversion Algorithms

Within the putamen region, a region with the highest change detected with most pipelines, all pipelines detected over‐time susceptibility changes signed consistently with H&S. Focusing specifically on the putamen region, we replicated the inter‐algorithmic paired *t*‐test analysis previously conducted within the reproducibility error global metric for the normalized over‐time change findings.

Within the putamen region, PDF detected the highest median over‐time change (272.22 [86.16 IQR] ppb) across all BFR algorithms. However, our voxel‐wise over‐time change analysis revealed substantial over‐DGM artifacts associated with PDF (Figure 7—last row). To avoid a biased comparison, we chose RESHARP, which had the second‐highest median over‐time change (204.24 [62.17] ppb), for statistical comparison (*t*‐test) with other BFR algorithms (not compared to PDF). LBV (156.28 [48.09] ppb) and V‐SHARP (152.20 [45.70] ppb) exhibited significantly lower over‐time changes (*q* ≤ 0.01) compared to RESHARP. Differences in over‐time changes between SHARP (186.26 [36.18] ppb) and iHARP (175.70 [58.04] ppb) did not reach significance in comparison to RESHARP (*q* ≥ 0.30).

Regarding inversion algorithms, DeepQSM (median over‐time change across BFR algorithms = 268.25 [38.97 IQR] ppb—highest in putamen) was compared to all other inversion algorithms across all BFR algorithms. No significant differences were observed (*q* ≥ 0.10) for FANSI (170.40 [50.16] ppb), WH‐FANSI (169.41 [103.75] ppb), DeepQSM* (174.86 [39.60] ppb), L1‐QSM (197.11 [119.64] ppb), and HD‐QSM (167.93 [36.28] ppb), while all others detected significantly lower susceptibility changes over time (*q* ≤ 0.05) compared to DeepQSM.

### 4.5 Sensitivity

**FIGURE 8:**
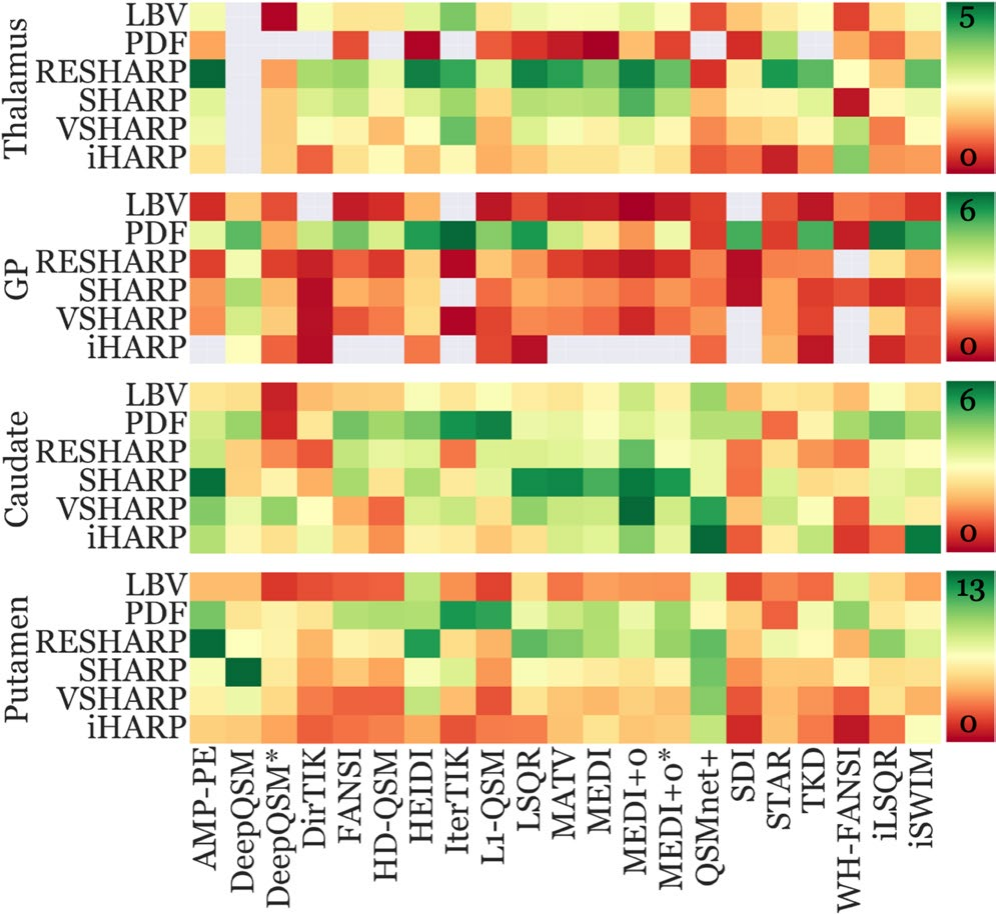
Pipeline sensitivity toward aging‐related susceptibility changes using WB reference. Each row corresponds to a combination of a BFR algorithm and DGM ROI. Each column represents an inversion algorithm. Susceptibility changes incompatible with H&S were excluded (gray box) to facilitate visualization. Each of the four regions (blocks of rows) has its own color bar on the right, with green indicating high sensitivity and red low. See Figure S22 for an annotated version of this figure.

Figure 8 represents sensitivity findings using WB referencing. WM and CSF‐referenced findings can be found in Figure S23 and Figure S24, respectively.

#### 4.5.1 Sensitivity Varied Between Anatomical Regions

WB‐referenced sensitivity significantly varied between DGM regions (*q* ≤ 0.02) with the highest median sensitivity within the putamen region (5.04 [3.49 IQR]), as expected based on the high putative over‐time iron changes (see above). The putamen was followed by the caudate (3.13 [1.38]), thalamus (2.25 [1.25]), and GP (1.20 [1.36]).

#### 4.5.2 Pipelines With RESHARP and PDF Demonstrated the Highest Sensitivity

Most pipelines yielded medium to high sensitivity values in all regions, but sensitivities varied substantially between pipelines within each region. None of the pipelines were consistently better than other pipelines across all regions. RESHARP demonstrated high sensitivities with 62% of the inversion algorithms (13 of 21) in the thalamus. In the remaining regions, PDF yielded higher sensitivities with most inversion algorithms (10, 11, and 9 out of 21 inversions in GP, caudate, and putamen, respectively) compared to RESHARP.

**FIGURE 9:**
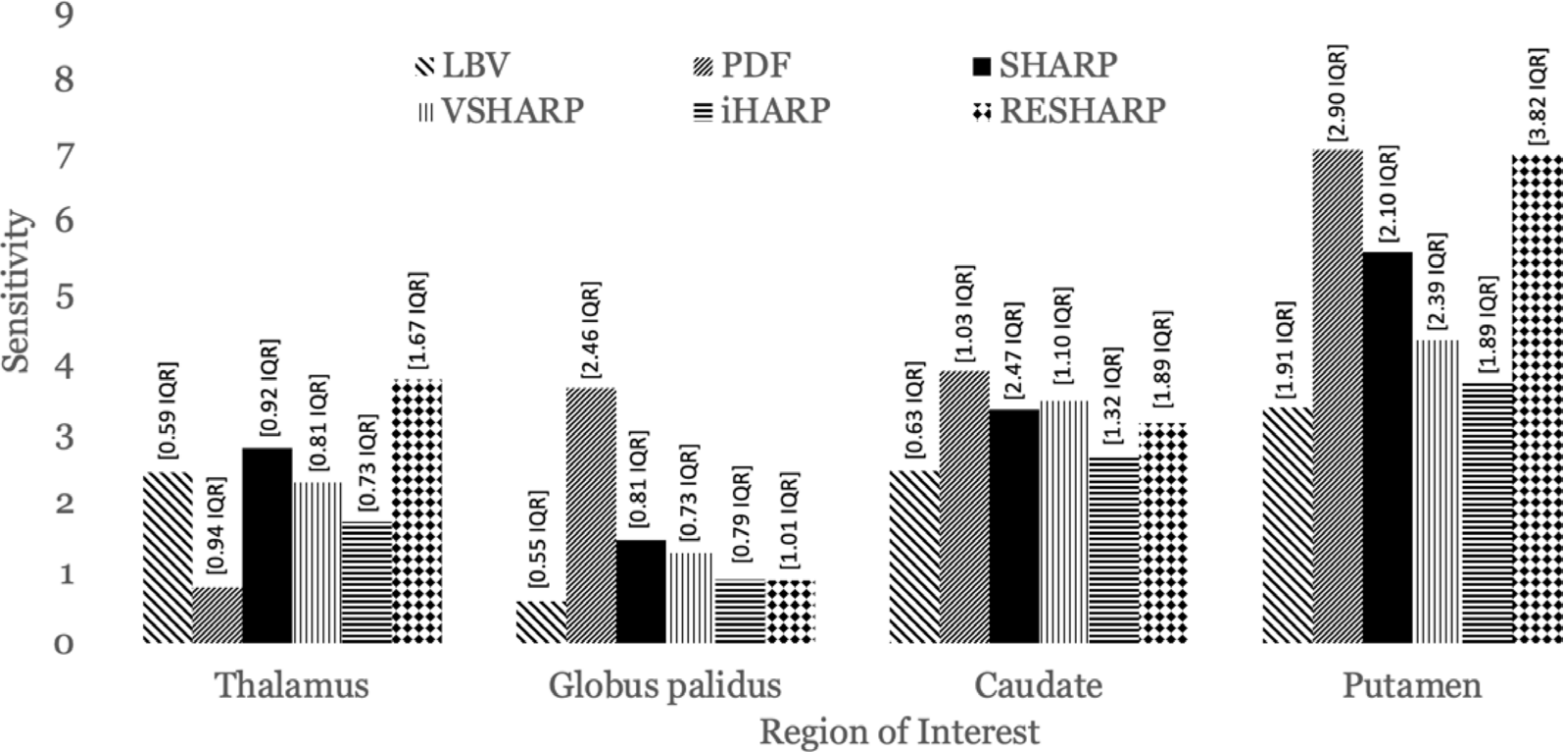
WB‐referenced median sensitivity of each BFR algorithm across all inversion algorithms. *Y*‐axis portrays the median sensitivity. Inversion algorithms detecting temporal susceptibility changes inconsistent with H&S were excluded from the median.

Within the putamen, pipelines with RESHARP and PDF yielded similar median sensitivities (7), which were on average 36% higher than pipelines using other BFR algorithms. Within the GP region, PDF yielded the highest median sensitivity of 3.7 (on average 70% higher), while within the caudate, all BFR algorithms were similar, with PDF ranking the highest (3.9). Within the thalamus, RESHARP ranked highest with a median sensitivity of 3.85. Figure 9 summarizes these findings.

#### 4.5.3 High Sensitivity Across All Regions With RESHARP

**FIGURE 10:**
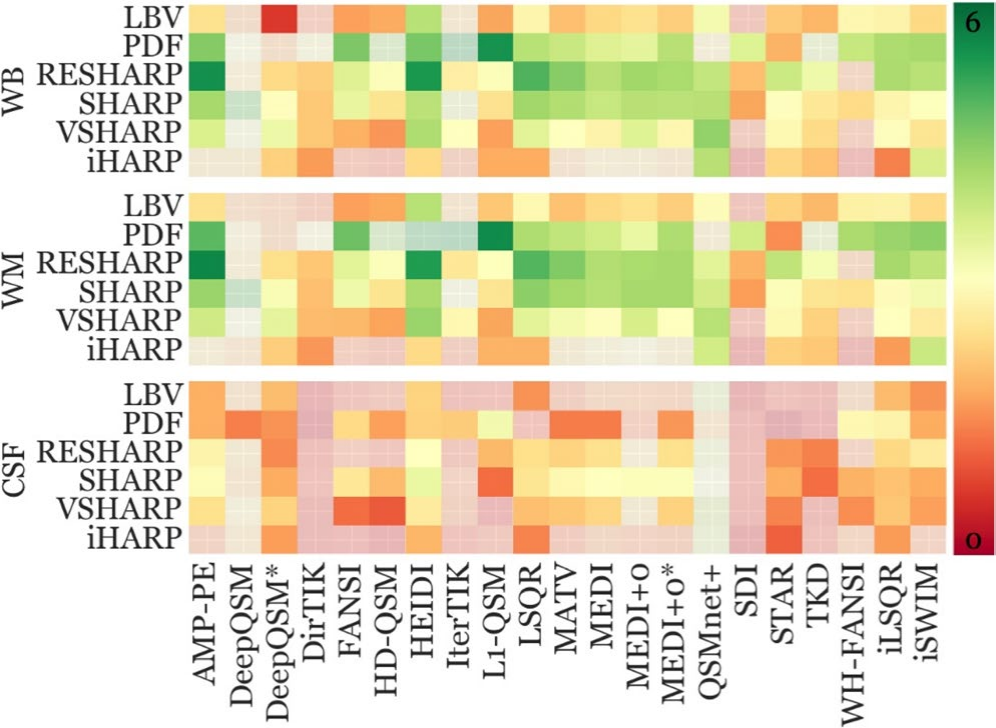
Global performance sensitivity metric as defined in Equation (7) (right). Each row corresponds to a combination of a BFR algorithm and reference region, and each column represents an inversion algorithm. Pipelines that yielded regional changes incompatible with H&S in any of the regions are translucent instead of gray boxes (distinguishable by crisscross within the boxes) in the other figures (due to numerous exclusions) to facilitate visualization. Green indicates high sensitivity, and vice versa for red. See Figure S25 for an annotated version of this figure.

Figure 10 illustrates the global pipeline sensitivity metrics. Results largely mirrored the reproducibility error findings (Figure 5), particularly regarding the impact of the reference regions. WB and WM referencing exhibited similar effects on the overall DGM sensitivity (*p* = 0.50), whereas CSF‐referencing resulted in markedly reduced sensitivities (*p* < 0.001).

The combination of RESHARP with AMP‐PE yielded the overall highest global sensitivities with either WB ($P_{d}=5.49$) or WM referencing (5.62), followed by RESHARP with HEIDI, and RESHARP with LSQR (all $P_{d}\geq 5.00$). The global sensitivity of the PDF + L1‐QSM pipeline ($P_{d}=5.45$) was deemed unreliable due to pronounced regional over‐time changes (Figure S12) that were attributed to artifacts, as illustrated with other PDF‐based pipelines in Figure 7; therefore, its sensitivity was not further considered for benchmarking.

Using CSF‐referencing, the highest sensitivities were observed for RESHARP with HEIDI, SHARP with HEIDI, and SHARP with all variants of MEDI (MEDI, MEDI+0, and MEDI+0*—all $P_{d}\geq 3.00$). Once again, the sensitivity of the PDF + L1‐QSM pipeline was not further considered due to the aforementioned reason.

Overall, SHARP‐based BFR algorithms (SHARP, V‐SHARP, and RESHARP) outperformed other BFR algorithms with respect to sensitivity and the number of pipelines that detected over‐time changes consistent with H&S (19 each for SHARP and RESHARP, 18 for V‐SHARP). RESHARP‐based pipelines exhibited the highest sensitivities across all BFR algorithms (median across inversion algorithms: 3.85 [1.14 IQR] for WB, 3.90 [1.13] for WM, 2.36 [0.63] for CSF), except for pipelines with deep learning‐based inversion algorithms, which either yielded H&S‐inconsistent results (DeepQSM and QSMnet+) or marginally better results with SHARP or V‐SHARP (DeepQSM*).

iLSQR, across all regions, was the only inversion algorithm to detect longitudinal changes consistent with H&S, regardless of BFR and reference region. Additionally, iSWIM, STAR, LSQR, and L1‐QSM with WB and WM referencing, HEIDI with WB and CSF‐referencing, and DeepQSM* with CSF‐referencing also demonstrated similar consistency in terms of H&S‐consistent temporal changes across all regions (see non‐translucent boxes in Figure 10).

### 4.6 Rater Evaluation of the 95th Percentile Pipelines

After evaluating pipelines based on the WB‐referenced global sensitivity metric, which detected over‐time changes consistent with H&S predictions (*N* = 97; top panel—Figure 10), the following pipelines met or exceeded the 95th percentile threshold ($P_{d}\geq 4.59$): RESHARP with AMP‐PE (5.49), PDF with L1‐QSM (5.45), RESHARP with HEIDI (5.38), RESHARP with LSQR (4.97), and PDF with HEIDI (4.60). PDF + L1‐QSM was excluded from subsequent evaluations because of the aforementioned reasons (within‐DGM artifacts).

All raters ranked the combination of RESHARP and HEIDI the highest (ranked first in 29 of 30; 97%, see Figure S26). Intra‐rater agreement was 0.90 (excellent). Inter‐rater agreement was 0.28 (*p* < 0.005; fair agreement; primarily driven by mixed rankings for LSQR and AMP‐PE).

We conducted a post hoc interview with all raters 2 weeks after the second evaluation to understand why raters preferred susceptibility maps calculated with HEIDI over those from AMP‐PE and LSQR. All raters responded that HEIDI's susceptibility maps were the sharpest and most homogeneous, while LSQR exhibited streaking artifacts, inhomogeneity, and a higher noise level. The AMP‐PE maps were criticized for being blocky (pixelated when zoomed in), having lower boundary definition, and suffering from blurry streaking artifacts. Raters' individual comments can be viewed in Table S3.

## 5 Discussion

This study systematically investigated QSM pipelines concerning scan‐rescan reproducibility error and sensitivity toward over‐time changes in brain susceptibility.

### 5.1 Study Design

The fundamental premise of this study was that susceptibility changes in the DGM due to aging are dominated by alterations in tissue iron, as originally characterized half a century ago by H&S (Hallgren and Sourander 1958). However, while susceptibility measurements provide valuable insights into tissue properties, they differ fundamentally from heme iron concentrations measured by H&S. Magnetic susceptibility reflects the magnetic properties of the tissue rather than directly quantifying iron. As such, exact convergence of QSM and histochemical iron assessment cannot be expected. We suggest contextualizing the over‐time changes in the present study as a surrogate for an algorithm's general ability to detect changes in susceptibility between two sample distributions, such as between a patient and a control group in a clinical study setting.

Over‐time susceptibility changes obtained with most pipelines showed medium to high quantitative correlations with putative iron changes (Figure 6—top panel). However, the considerable variability in observed over‐time susceptibility changes across pipelines suggests that it may be challenging to quantitatively compare findings of longitudinal studies that employed different pipelines. Hence, while interpretive study outcomes, such as the statistical significance of group differences, are likely reproducible across pipelines, caution is warranted when comparing quantitative results, such as regional average susceptibility values, across studies using different pipelines. Future research should examine how pipeline choice impacts both the quantitative measurements and qualitative interpretations of cross‐sectional and longitudinal studies, as well as the reproducibility of previously reported findings in the literature. The computational framework developed in the present study could be applied directly to other imaging datasets and may be useful to shed further insight on the pipeline dependence of QSM.

While correlations were high, the estimated QSM‐based changes were at least twice as large as the predicted changes (Figure 6—bottom panel). The reasons for this substantial overestimation of aging‐related iron changes relative to H&S (Figure 6) remain unclear. Plausible explanations are: (1) differences in iron metabolism between the cohorts due to environmental, cultural, and lifestyle factors (assessed over half a century apart in different regions; ours = North America vs. H&S = Scandinavia) (Ahern et al. 2024; Gustavsson et al. 2024; Zachariou et al. 2025); (2) differences in sex representation (19:4 F:M, ratio unknown for H&S), as females exhibit slower age‐related iron increases, likely due to hormonal differences associated with menopause (Persson et al. 2015); (3) nonlinear age‐related iron changes, such as thalamic plateauing or decline in advanced age (Burgetova et al. 2021; Treit et al. 2021), could further amplify these discrepancies; (4) H&S's population‐average fitting functions may not be adequate descriptions of the *intra‐subject* over‐time dynamics (see Section 5.7.1); (5) lastly, while our inclusion criteria targeted an age range with stable myelin concentration (Cagol et al. 2024; Dvorak et al. 2021), aging‐related demyelination (Biel et al. 2021; Khodanovich et al. 2023) may still have affected our over‐time changes, although this is relatively unlikely as it would have also confounded the thalamic over‐time decline, which was largely consistent with H&S.

The high consistency of predicted *cross‐sectional* iron values with QSM‐based iron concentrations from most pipelines (slopes between 0.9 and 1.1 in Figure S18, respectively—bottom) must be interpreted with caution because Langkammer et al. (2012) determined the susceptibility‐to‐iron conversion factor itself using QSM (SHARP + HEIDI). However, a slope close to one and very high correlation suggest that iron values reported by H&S are still representative for those found in today's population and, second, provides further support for the study's premise that DGM susceptibility is dominated by susceptibility effects from tissue iron.

### 5.2 Reproducibility Error

While identifying the methodological causes of scan‐rescan variability was beyond the scope of our study, the absence of distinct spatial or anatomical patterns in our voxel‐wise analysis (Figure 4) suggests that random effects, such as noise amplification and motion artifacts, are the primary contributors to this variability.

Furthermore, consistent with a previous study (Rua et al. 2020), we contend that opting for a single‐echo sequence may provide lower reproducibility error of QSM compared to multi‐echo sequence(s) used in previous studies (Deh et al. 2015; Feng et al. 2018; Lin et al. 2015; Liu et al. 2024; Naji et al. 2022; Rua et al. 2020; Santin et al. 2017; Spincemaille et al. 2019) and as recommended in the recent QSM consensus (QSM Consensus Organization Committee et al. 2024). This apparent advantage may stem from lower readout bandwidth and the specific echo time typically chosen in single‐echo acquisitions, which is usually shorter than the later echoes in multi‐echo sequences, thus reducing the propagation of excessive phase noise when echo weighting is imperfect. In general, the quality of multi‐echo data depends on several factors, including voxel‐wise noise weighting in the field map calculation (QSM Consensus Organization Committee et al. 2024). Although we do not anticipate substantial changes in the overall performance of the tested pipelines based on sequence configuration, echo weighting could influence conclusions about *regional* differences. Future research is needed to better understand the impact of pulse sequence parameters on the pipeline's sensitivity.

### 5.3 High Performance of SHARP‐Based BFR Algorithms

BFR algorithms have not been evaluated with the same scrutiny as dipole inversion algorithms in the past (Fortier and Levesque 2018; Langkammer et al. 2018; Milovic et al. 2023; QSM Challenge 2.0 Organization Committee et al. 2021; Schweser et al. 2017) likely because it was assumed that their effect on susceptibility map reconstruction quality is small compared to that of the dipole inversion. Our observations confirmed this notion. While we identified spatially slowly varying remnant fields as a confounder of scan‐rescan reproducibility error, these remnant fields, induced or left uncorrected by the BFR algorithms, could only be visualized through difference images (e.g., in Figure 7) and were indiscernible on both the BF‐corrected field maps and the final susceptibility maps (Figures 1 and 2, respectively) (Özbay et al. 2017).

After correcting for systematic underestimation, the regional reproducibility error was overall relatively similar across pipelines (Figure 3). The slightly increased robustness of SHARP‐based BFR algorithms, also mentioned in the QSM consensus (QSM Consensus Organization Committee et al. 2024), may be related to the intrinsic spatial averaging of the SMV computation in these methods, as well as their mild low‐pass filtering capabilities. The SMV computation may be less sensitive to field errors and noise close to the brain's surface compared to other methods (Schweser et al. 2017). The low‐pass filtering may effectively suppress residual BFs or transceiver‐phase contributions that can interfere with the dipole inversion. However, residual transceiver‐phase contributions in our data may have amplified the importance of low‐pass filtering in these algorithms, and the difference between BFR algorithms may be less pronounced when the transceiver phase is removed analytically, for example, using multi‐echo data (QSM Consensus Organization Committee et al. 2024; Sun and Wilman 2014).

### 5.4 Remarkable Reproducibility of QSMnet+

QSMnet+ consistently achieved the highest reproducibility independent of the BFR (Figure 5). This finding was in line with the original publication's assertion of high reproducibility (Yoon et al. 2018), as they anticipated their network to deliver such performance based on consistent WM contrast observed in multiple head orientation QSM outputs from QSMnet. This analogy suggests that the high reliability observed in the WM extends to the DGM. Using V‐SHARP and WB referencing, as in the original publication, the reproducibility error found in the present work (in caudate, putamen, and GP = 0.040, 0.036, and 0.124 ppm, respectively) fell within a close range to that reported in the original publication of the precursor method, QSMNet (Yoon et al. 2018) (0.034, 0.054, and 0.130 ppm, respectively; QSMnet+ publication [Jung et al. 2020] did not investigate reproducibility). While an investigation of the reasons for the high reproducibility and BFR independence of QSMnet+ was beyond the scope of our study, the responsible features of the algorithm may hold the key to lower reproducibility error and higher sensitivity of QSM. For example, the low reproducibility error of WM measurements may have positively affected referencing‐related variation.

### 5.5 Complex Interplay Between BFR and Dipole Inversion

The sensitivity (Figure 8) toward over‐time changes (Figure S11) demonstrated a more profound interplay between BFR and dipole inversion algorithms than the reproducibility error metric. While mechanistic investigations were beyond the scope of the study, changes in brain anatomy and overall susceptibility distribution from baseline to follow‐up are likely contributors to this interplay. While brain anatomy and susceptibility were identical between scans in the scan‐rescan experiments, they were naturally affected by a decade of aging in our longitudinal experiments. BFR artifacts that are in some way linked to brain anatomy, for example, through the BFR masks, through features of the BF itself, or through spatial‐frequency filtering (Schweser et al. 2017), can be expected to affect over‐time changes, whereas their effect on scan‐rescan experiments might be negligible. This aspect of QSM reproducibility has wide‐ranging implications for the use of QSM in clinical studies but is currently only poorly understood. The substantial variation in sensitivity between pipelines suggests that future QSM benchmarking initiatives should focus on full pipelines rather than single steps within the pipeline. We speculate that differences in dipole inversion algorithms' ability to handle BFR artifacts are likely related to the employed regularization approaches.

### 5.6 Effect of Referencing on Reproducibility Error

Our study confirmed the recent consensus statement (QSM Consensus Organization Committee et al. 2024) that larger reference regions, particularly WB referencing, yield more stable and reproducible susceptibility values than smaller regions. While CSF susceptibility is unaffected by most diseases, CSF‐referencing suffers from numerous challenges that were discussed previously (QSM Consensus Organization Committee et al. 2024), including a potential age dependency (Sun et al. 2024). In particular, CSF‐referencing resulted in the lowest reproducibility and sensitivity in the present study.

A recent study demonstrated that utilizing the global CSF regions, that is, bilateral ventricles and subarachnoid CSF, improved the reproducibility in a scan‐rescan setting compared to using only the bilateral ventricles (Dimov et al. 2022). Unfortunately, this approach could not be applied in the present study due to the inability to reliably segment the subarachnoid space in the absence of R_2_* maps.

Furthermore, while MEDI+0 aims to address certain limitations of CSF‐referencing by enforcing a homogeneity constraint for the CSF susceptibility, the CSF constraint showed limited efficacy in improving reproducibility compared to the same method without the CSF constraint or the conventional MEDI (Liu et al. 2012) method in our study. This observation mirrored the results presented in the original MEDI+0 publication (figure 5 in Liu et al. 2018).

Future research should explore the factors influencing CSF‐referencing performance and its potential variability across different cohorts and methodologies. For example, a recent study highlighted several advantages of using CSF as a reference region, including its higher reproducibility in QSM, which contrasts with our findings (Straub et al. 2017).

### 5.7 Limitations

#### 5.7.1 Premise of the Study Design

Our study relied on the premise that population‐average trajectories of *cross‐sectional* brain iron measurements can be used to estimate population‐average longitudinal changes in brain iron. While the premise appears plausible, it is not supported by strong evidence. In fact, the high inter‐subject variation in brain iron observed in cross‐sectional studies leaves the possibility that *longitudinal* changes estimated from few discrete time points over a relatively short time span differ substantially from population‐average *cross‐sectional* trajectories. To the best of our knowledge, no studies have compared cross‐sectional trajectories with longitudinal dynamics. However, these limitations are primarily relevant to our comparison of susceptibility values to H&S‐based predictions of iron concentrations and do not affect our conclusions about reproducibility and sensitivity.

However, sensitivity metrics may have been biased by the covariance of reconstruction artifacts and age. For example, pipelines that produce artifacts affected by the shape and volume of brain regions may have created systematic differences between baseline and follow‐up susceptibility maps that were linked to aging‐related alterations in brain morphology (Bashyam et al. 2020), an effect previously described in SWI phase imaging (Schweser, Dwyer et al. 2013). Such artifacts may have inflated the observed effect sizes for some algorithms and brain regions, or be responsible for the H&S‐inconsistent outcomes with some pipelines. Since our study design did not allow for investigating such effects, future studies should determine if QSM is prone to morphological changes.

#### 5.7.2 Impact of Algorithmic Parameters

Due to the extensive number of pipelines investigated, optimization of each algorithm's parameters was infeasible. Consequently, it is possible that pipeline performance can be improved in some cases using optimized parameters. Since it is well‐known that algorithmic parameter choices in the dipole inversion step substantially affect the appearance of computed susceptibility maps (Langkammer et al. 2018; QSM Challenge 2.0 Organization Committee et al. 2021), we performed a preliminary post hoc analysis of the effect of dipole inversion regularization parameters. We computed WB‐referenced reproducibility and sensitivity metrics for MEDI (Liu et al. 2012) with all BFR algorithms across an extreme range of regularization parameters (Figure S27). We chose MEDI because of the widespread use of the algorithm and its well‐known sensitivity to the susceptibility map appearance on the regularization parameter. As expected, the appearance of the computed susceptibility maps differed greatly depending on the parameter values (Figure S28). However, despite these visual differences, the reproducibility and sensitivity metrics were largely unaffected. This counterintuitive finding may be explained by the ROI‐based calculation of the sensitivity and reproducibility metrics. Most regularization strategies penalize high‐frequency image noise and streaking artifacts and prioritize edge delineation. These image features have a limited effect on the mean ROI values. This preliminary investigation supports the generalizability of the present study and suggests that the lack of optimization of regularization parameters did not substantially bias either the present study or other clinical QSM studies in the literature. Moreover, both the robustness of pipeline sensitivity with respect to regularization parameters and the substantial differences in the appearance of the algorithms in the 95th sensitivity percentile challenge the practical relevance of past benchmarking initiatives for clinical research (Langkammer et al. 2018; QSM Challenge 2.0 Organization Committee et al. 2021), which prioritized quantitative reconstruction accuracy (with respect to a gold standard). Our data suggest that quantitative accuracy and visual quality do not necessarily imply high sensitivity toward susceptibility differences, and vice versa.

#### 5.7.3 Multi‐channel Combination and Phase Unwrapping

In this study, we did not investigate the effect of multichannel phase image combination or unwrapping algorithms (Robinson et al. 2017) on the final solution. We used best‐path unwrapping due to its widespread use, absence of the structural modification of phase images (Robinson et al. 2017), and because it is an exact phase unwrapping method recommended by the 2023 QSM consensus (QSM Consensus Organization Committee et al. 2024). Including different unwrapping algorithms would have rendered the computational cost and complexity of the presentation of the results in this study intractable. For the same reason, we also did not investigate different brain mask creation algorithms. While our quality control did not reveal any issues within the resulting BFR maps, the mask creation may still have affected the BFR performance.

#### 5.7.4 Single‐Site Data

Another limitation of the study is the reliance on data acquired at a single site with a single pulse sequence. While several multicenter studies have shown good reproducibility of QSM across sites and acquisition pulse sequences (Deh et al. 2015; Hinoda et al. 2015; Lancione et al. 2022; Lin et al. 2015; Liu et al. 2024; Naji et al. 2022; Rua et al. 2020; Spincemaille et al. 2019; Spincemaille et al. 2020; Yao et al. 2023), our findings may not fully generalize to all scanner and pulse sequence configurations. Specifically, our study used a single‐echo sequence, and transceiver‐phase contamination may have affected our results, as discussed above. Our sequence also used anisotropic voxel size, which may have affected the algorithmic performance in different ways.

## 6 Conclusion

Our study highlighted the importance of considering the performance of the entire QSM pipeline rather than testing the performance of individual components in isolation. Most of the pipelines included in this study reliably detected DGM iron changes over time. However, sensitivity varied substantially across pipelines, with the BFR being a major contributor to variations in sensitivity.

## Author Contributions

**Fahad Salman:** conceptualization, data curation, formal analysis, investigation, methodology, project administration, software, validation, visualization, writing – original draft, writing – review and editing. **Abhisri Ramesh:** methodology, software. **Thomas Jochmann:** methodology, software, writing – review and editing. **Mirjam Prayer:** methodology. **Ademola Adegbemigun:** methodology. **Jack A. Reeves:** formal analysis, investigation, writing – review and editing. **Gregory E. Wilding:** methodology, supervision. **Junghun Cho:** formal analysis, investigation, writing – review and editing. **Dejan Jakimovski:** data curation. **Niels Bergsland:** conceptualization, data curation, resources, software, writing – review and editing. **Michael G. Dwyer:** conceptualization, data curation, resources, software. **Robert Zivadinov:** conceptualization, data curation, resources, writing – review and editing. **Ferdinand Schweser:** conceptualization, funding acquisition, investigation, methodology, project administration, resources, software, supervision, validation, writing – original draft, writing – review and editing.

## Ethics Statement

The local Ethical Standards Committee approved the human experiments, and a written informed consent form was obtained.

## Conflicts of Interest

R.Z. has received personal compensation from Bristol Myers Squibb, EMD Serono, Sanofi, Mapi Pharma, Sana Biotechnologies, and Filterlex for speaking and consultant fees. He received financial support for research activities from Bristol Myers Squibb, EMD Serono, Mapi Pharma, Protembis, and Filterlex. M.G.D. received personal compensation from Bristol Myers Squibb, Novartis, EMD Serono, and Keystone Heart, and financial support for research activities from Bristol Myers Squibb, Novartis, Mapi Pharma, Keystone Heart, Protembis, and V‐WAVE Medical. The remaining authors declare no conflicts of interest.

## Supporting information


**Data S1.** Supporting information.

## Data Availability

The data that support the findings of this study are available on request from the corresponding author. The data are not publicly available due to privacy or ethical restrictions.
