# Supplementary material for: Sensitivity of Quantitative Susceptibility Mapping for Clinical Research in Deep Gray Matter
Source: Hum Brain Mapp. 2025 Apr 22;46(6):e70187. doi: 10.1002/hbm.70187 (PMC12012649; doi:10.1002/hbm.70187)
Supplement: Supplementary file 1 — Data S1. Supporting information. [file HBM-46-e70187-s001.docx]

Supplementary Figures


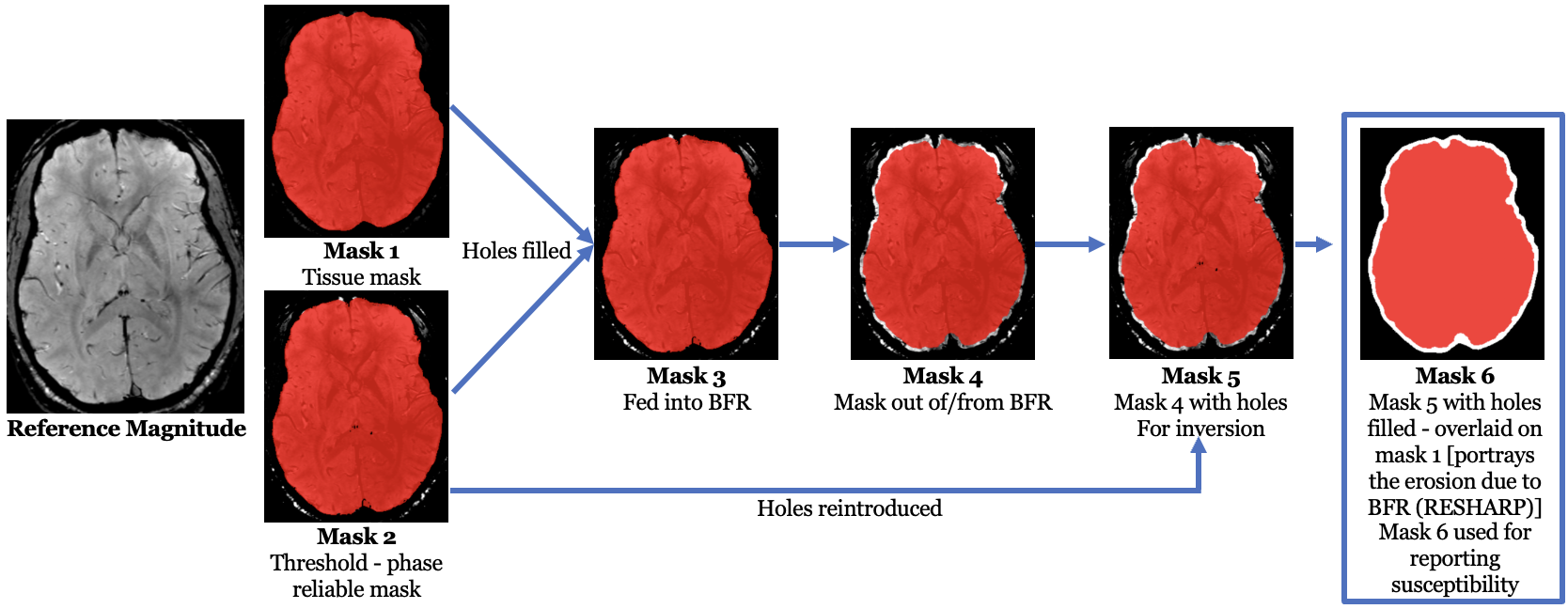


Supplementary Fig. 1. Schematic of the masking procedure utilized to generate susceptibility maps using RESHARP as an exemplary BFR. A representative subject’s (male, 25 years old) magnitude image in native space (far left) serves as a reference image, with all variations of masks overlaid on it used for QSM processing and analysis. Masks 4-6 are BFR specific due to their specific erosion.


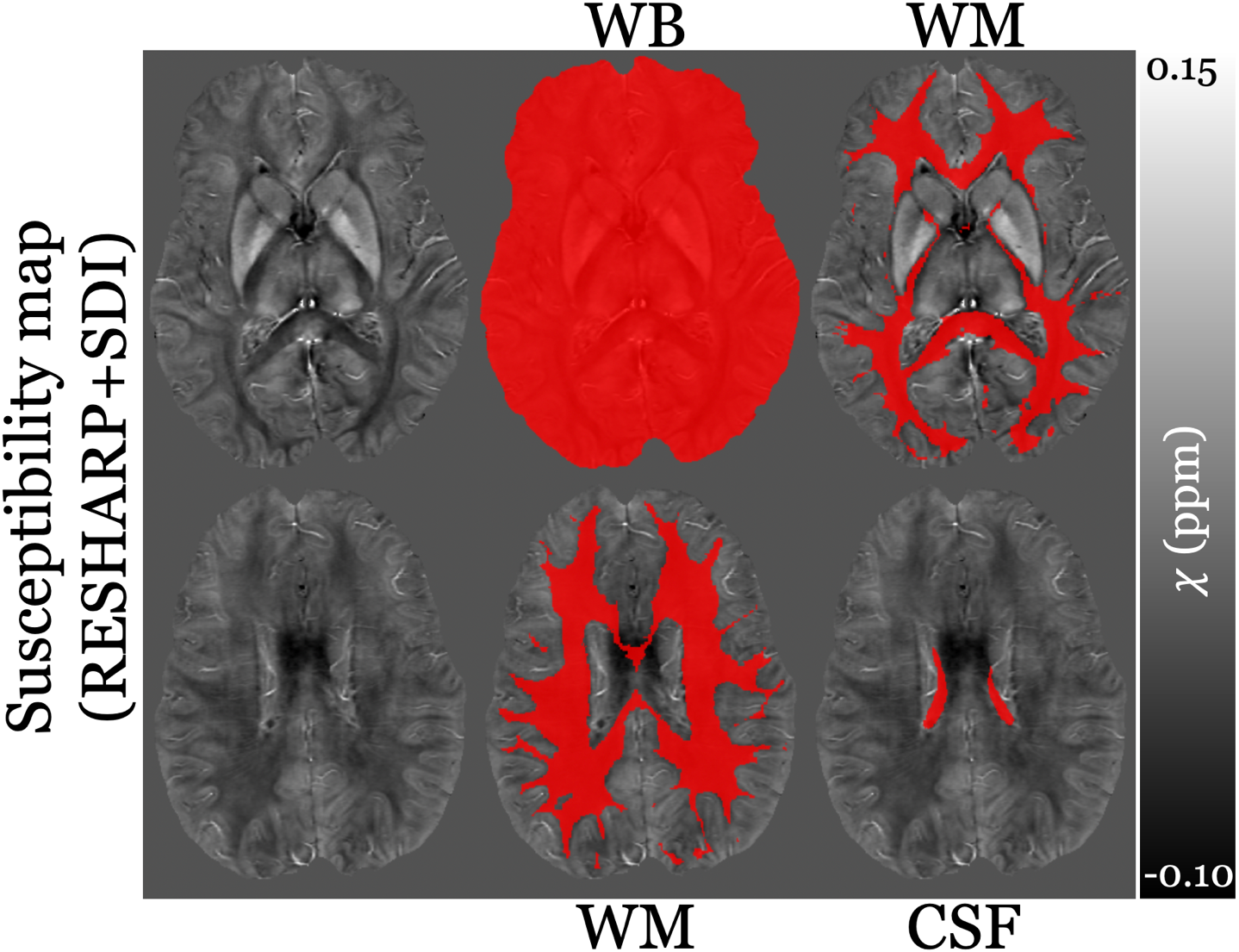


Supplementary Fig. 2. Reference region masks shown in native space of an exemplary subject’s (male, 25 years old) susceptibility map reconstructed using RESHARP as the BFR and SDI as the dipole inversion algorithm. Top row shows the slice that visualizes WB (middle) and WM (right) mask, while the bottom row visualizes slice with WM (middle) and CSF (right), respectively. Susceptibility map slices without reference masks can be viewed in the far left column.

**
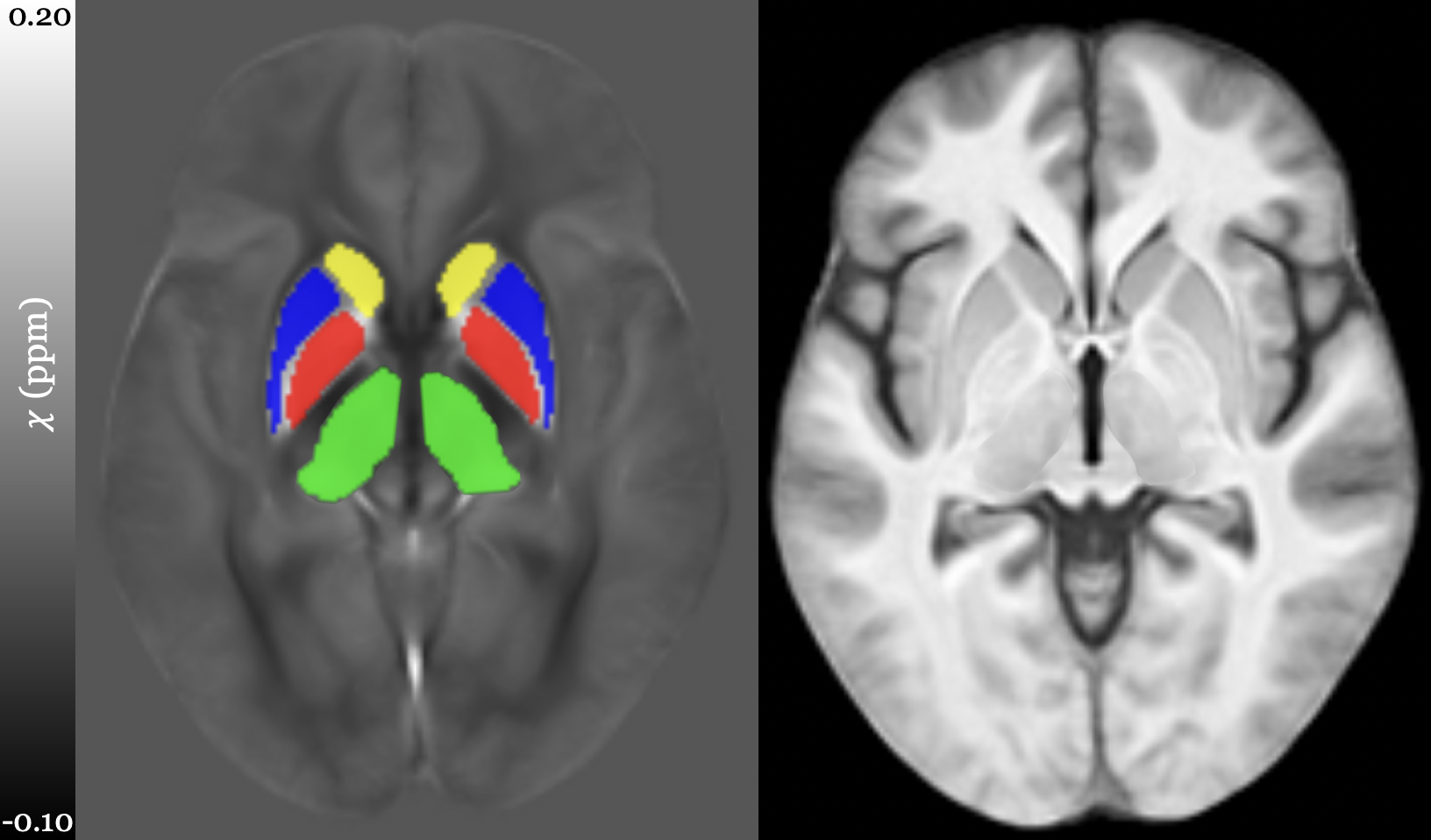
**

Supplementary Fig. 3. QSM and T1w contrast templates generated using the bi-parametric approach for this study, respectively. Bi-lateral ROIs shown are thalamus (green), GP (red), caudate (yellow), and putamen (blue).


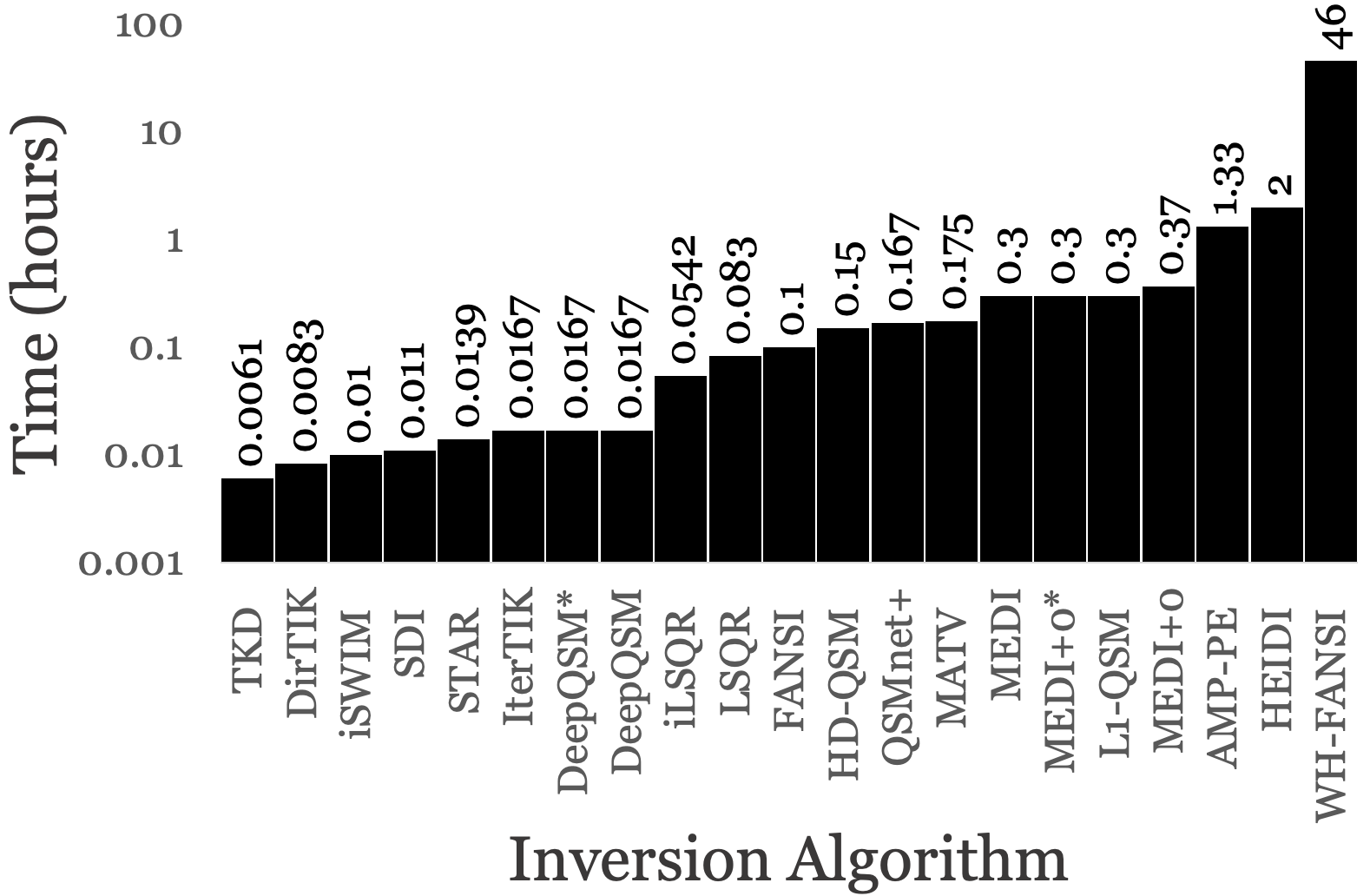
Supplementary Fig. 4. Log-scaled average dipole inversion reconstruction times in ascending order (4 CPUs used).


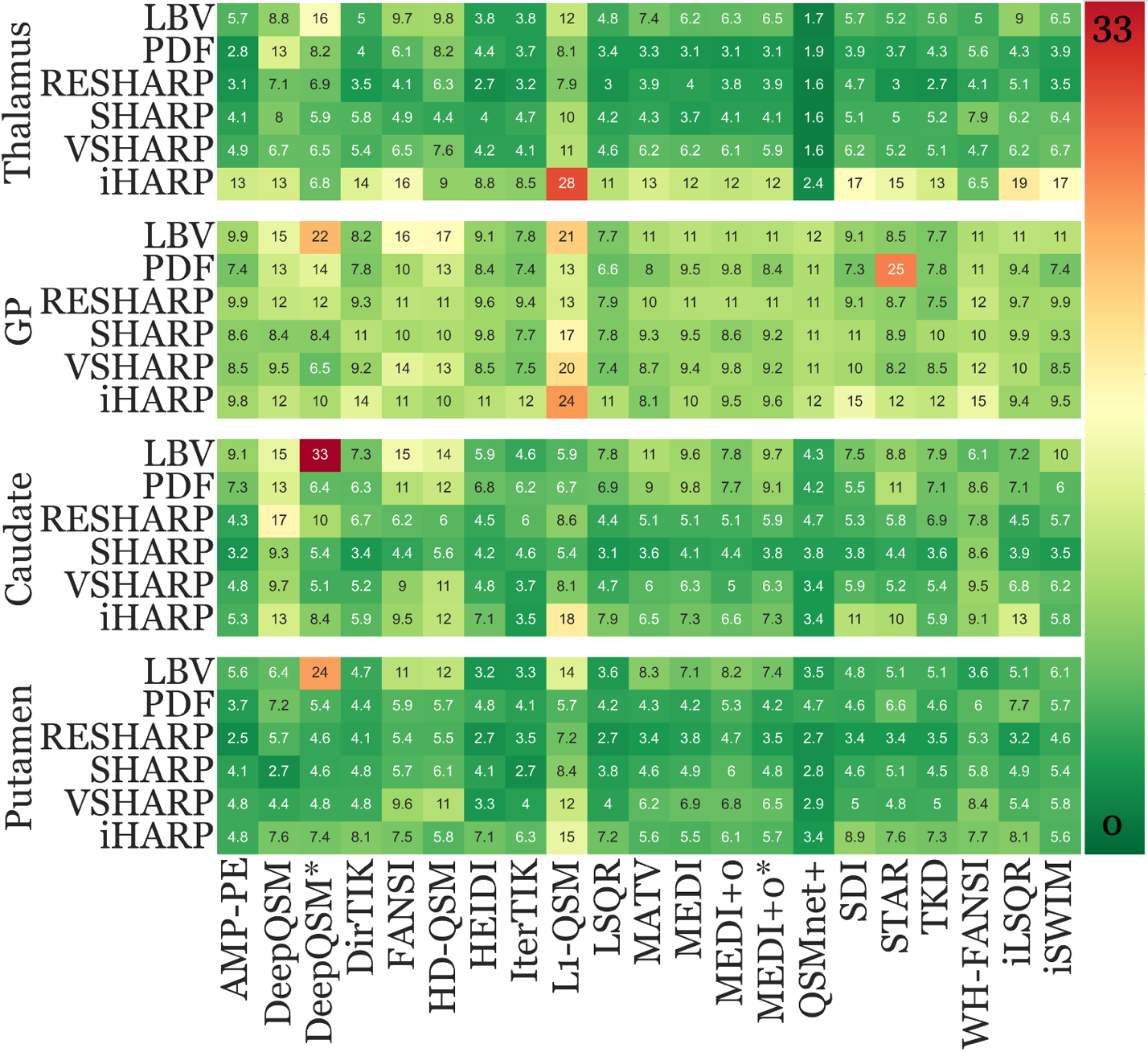
Supplementary Fig. 5. WB-referenced normalized reproducibility findings of each pipeline and region. Lower values (green) represent higher reproducibility, and vice versa for red. Each horizontal panel corresponds to one specific DGM region that is denoted at the left-hand side of the panel. Each row corresponds to a specific BFR algorithm (listed on the left-hand side) while each column represents an inversion algorithm (listed at the bottom).


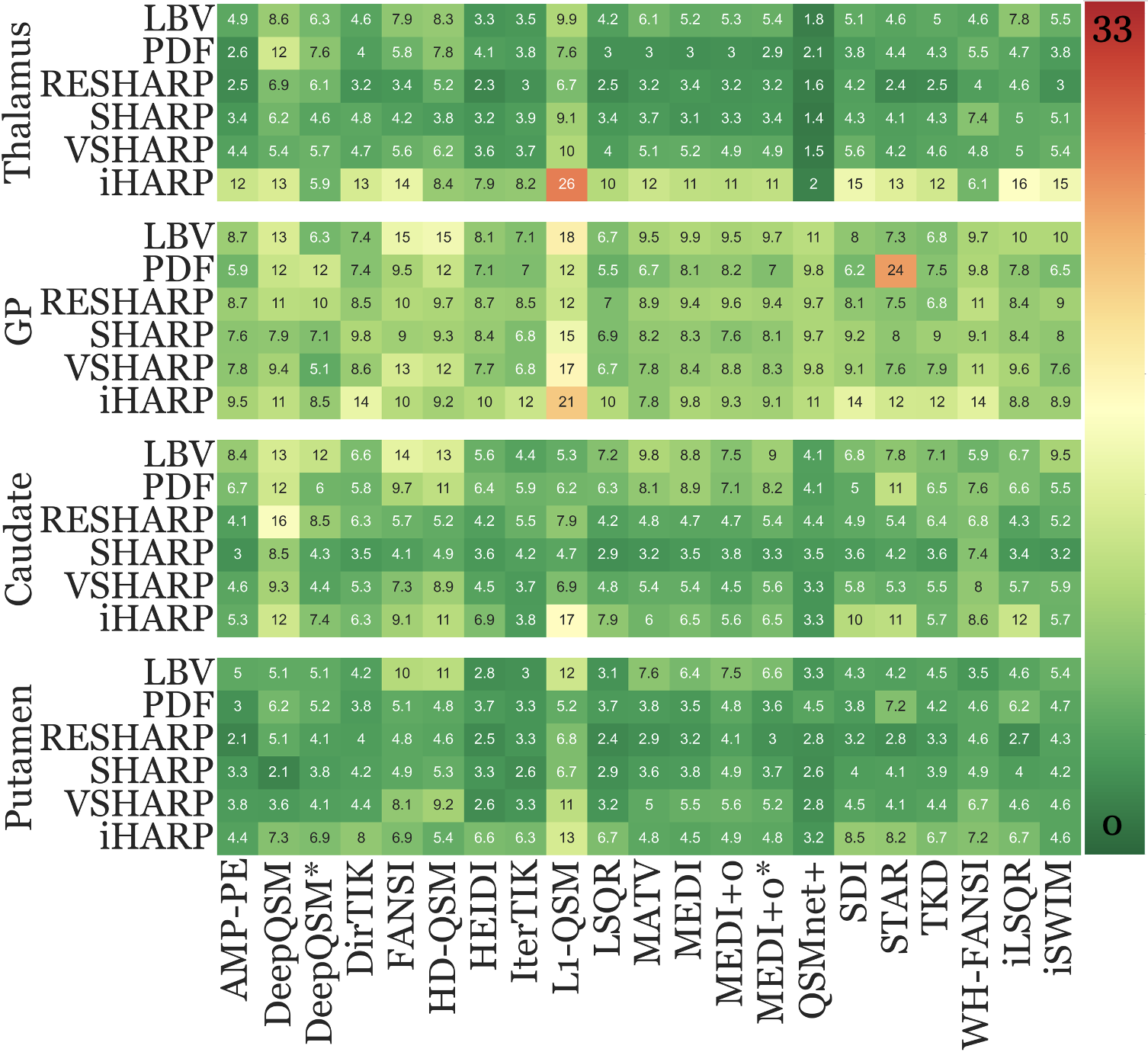
Supplementary Fig. 6. WM-referenced normalized reproducibility findings of each pipeline and region. Lower values (green) represent higher reproducibility, and vice versa for red. Each horizontal panel corresponds to one specific DGM region that is denoted at the left-hand side of the panel. Each row corresponds to a specific BFR algorithm (listed on the left-hand side) while each column represents an inversion algorithm (listed at the bottom).


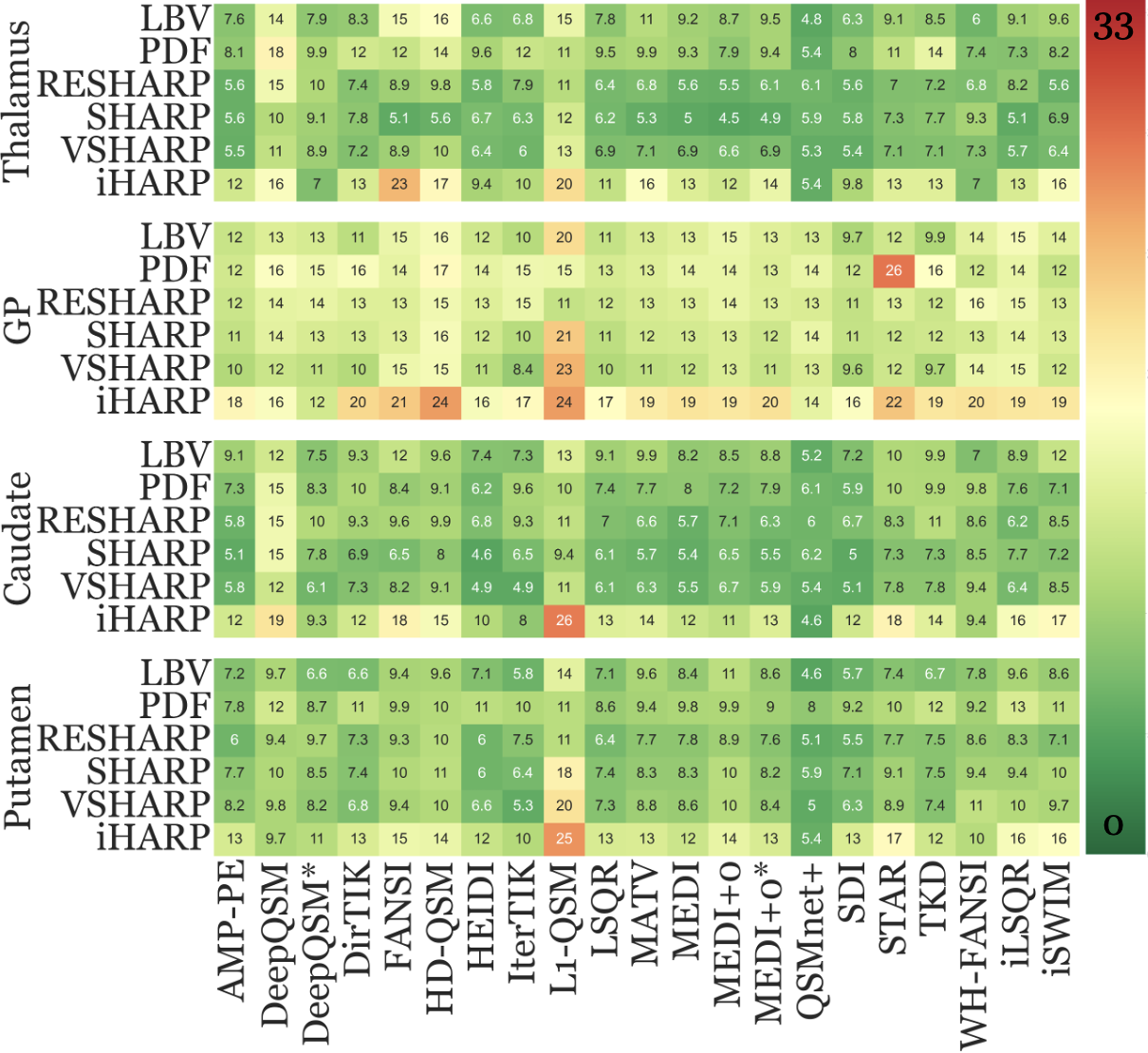
Supplementary Fig. 7. CSF-referenced normalized reproducibility findings of each pipeline and region. Lower values (green) represent higher reproducibility, and vice versa for red. Each horizontal panel corresponds to one specific DGM region that is denoted at the left-hand side of the panel. Each row corresponds to a specific BFR algorithm (listed on the left-hand side) while each column represents an inversion algorithm (listed at the bottom).


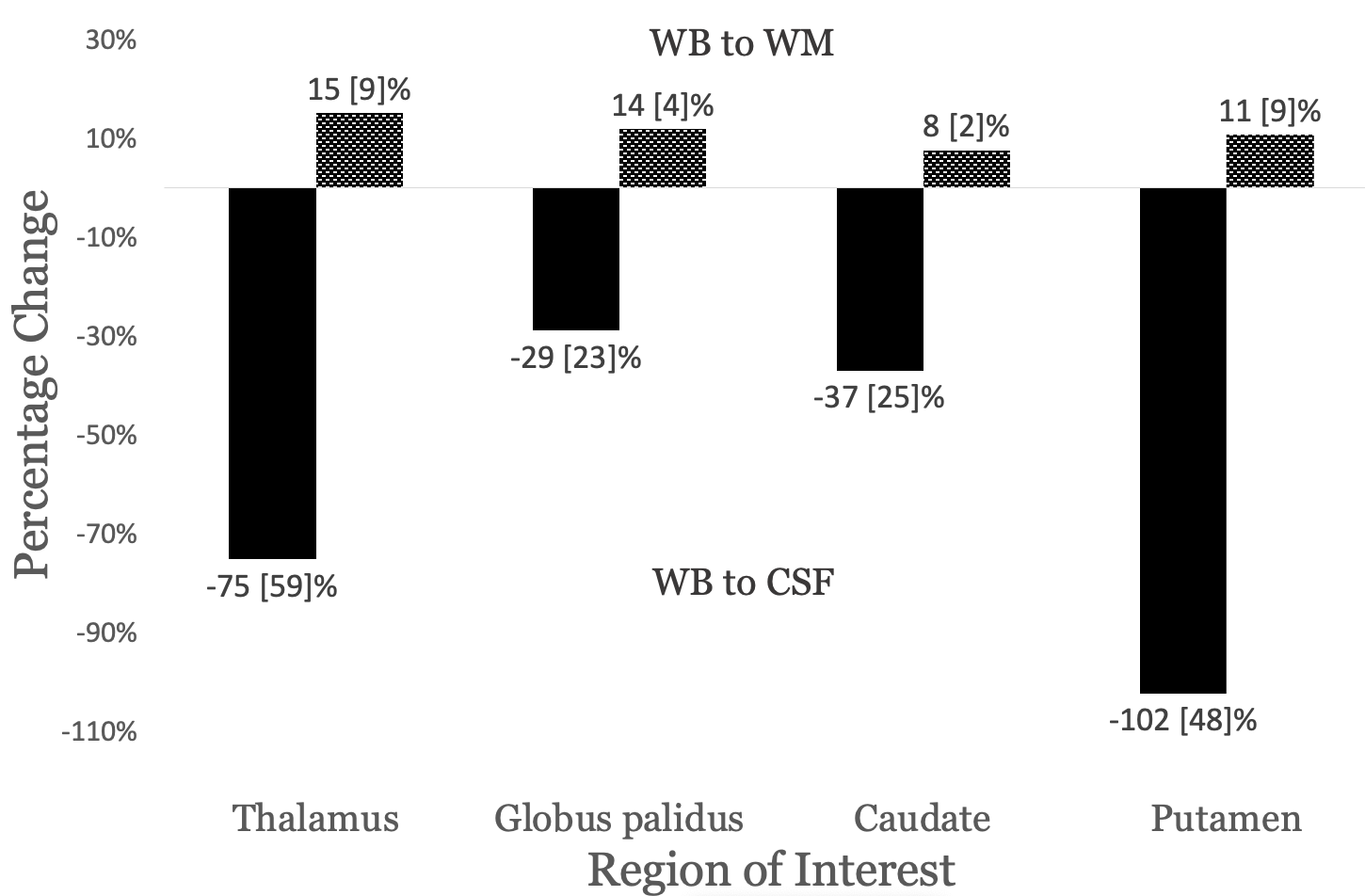
Supplementary Fig. 8. Median percentage increase/decrease (with [IQR]) in reproducibility across all inversion algorithms when the reference region was changed from WB to CSF (black) or WM (dotted black). CSF and WM-referenced normalized reproducibility findings were subtracted from brain-referenced findings to compute the percentage change in reproducibility due to the switch from the WB as the reference region. Y-axis shows the reproducibility change (in %) while x-axis shows the ROI. Top part of the plot shows the change in reproducibility when reference region was switched from WB to WM, while bottom shows from WB to CSF.


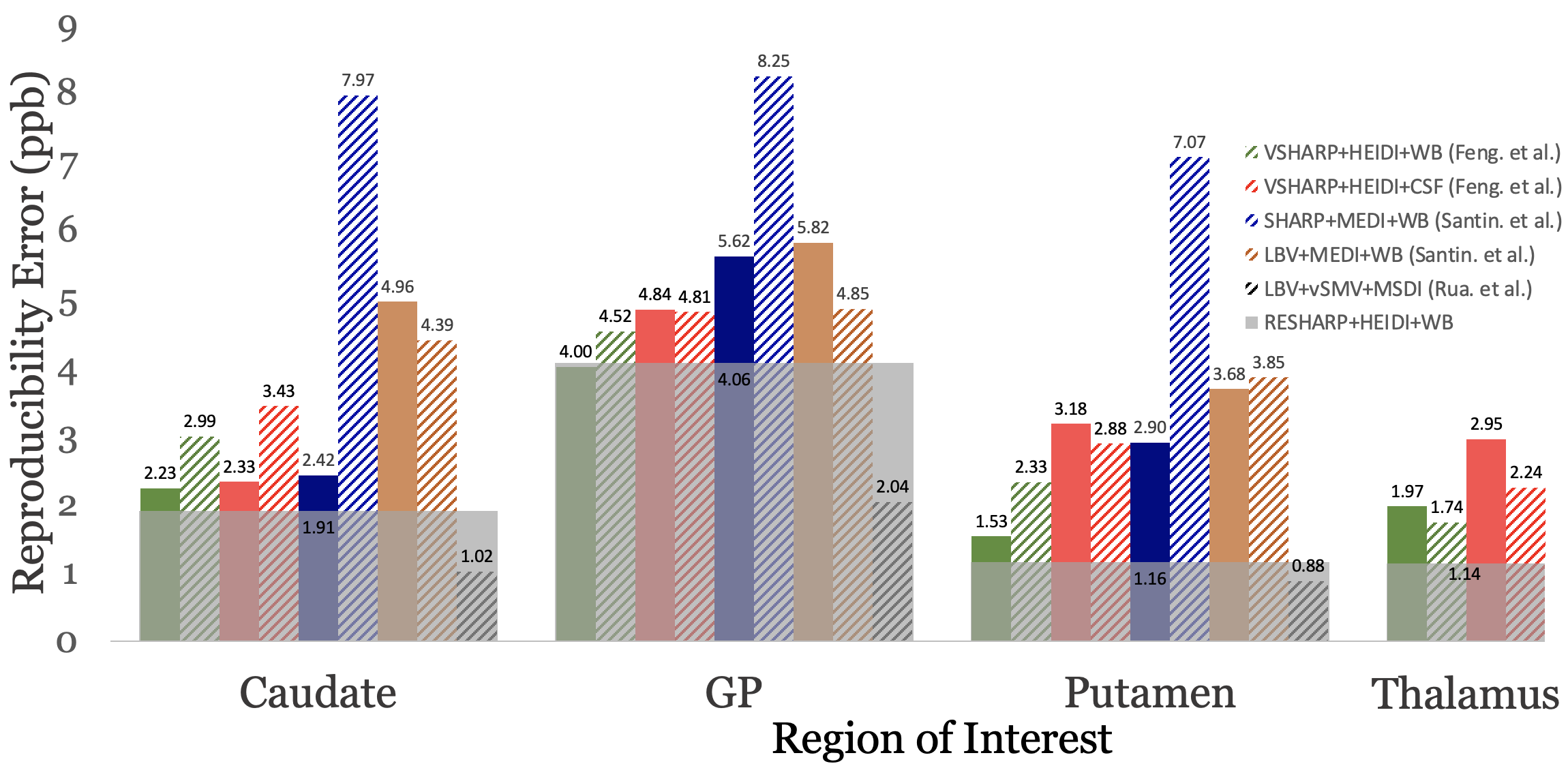
Supplementary Fig. 9. Comparison of raw scan-rescan reproducibility values (this study - solid bars) with previously reported (striped bar) pipeline reproducibility. X-axis displays the DGM region while y-axis displays the reproducibility in ppb. Reproducibility values from the present study were obtained with pipelines matched to previous studies, except for LBV+vSMV+MSDI, which was not investigated within our study. The transparent gray bar overlaid indicates one of the highest reproducibility findings across all pipelines investigated in the present study.


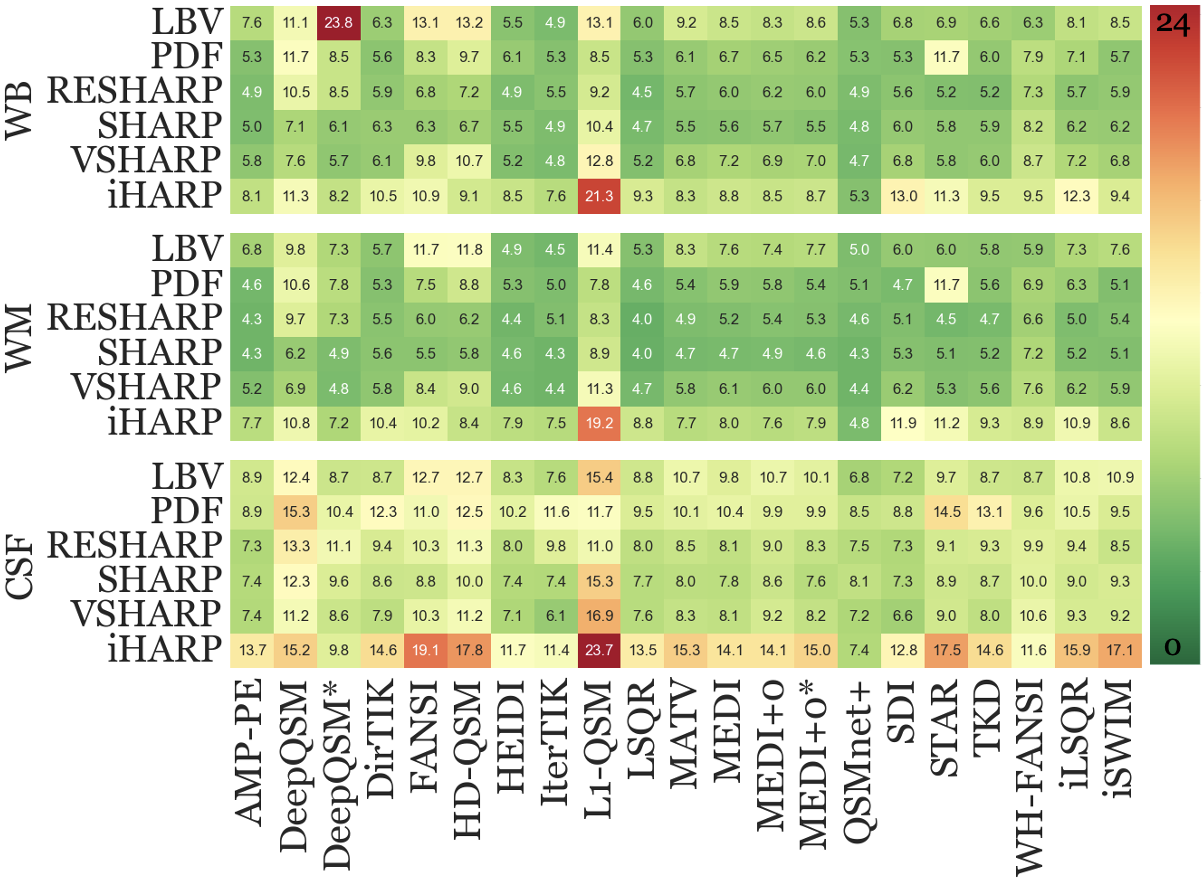
Supplementary Fig. 10. Global performance reproducibility heatmap according to Eq. 4. The color-coding and the arrangement of BFR and inversion algorithms mirrors that of supplementary Figs. 5-7. In this figure, each panel represents a specific reference region (listed on the left-hand side).


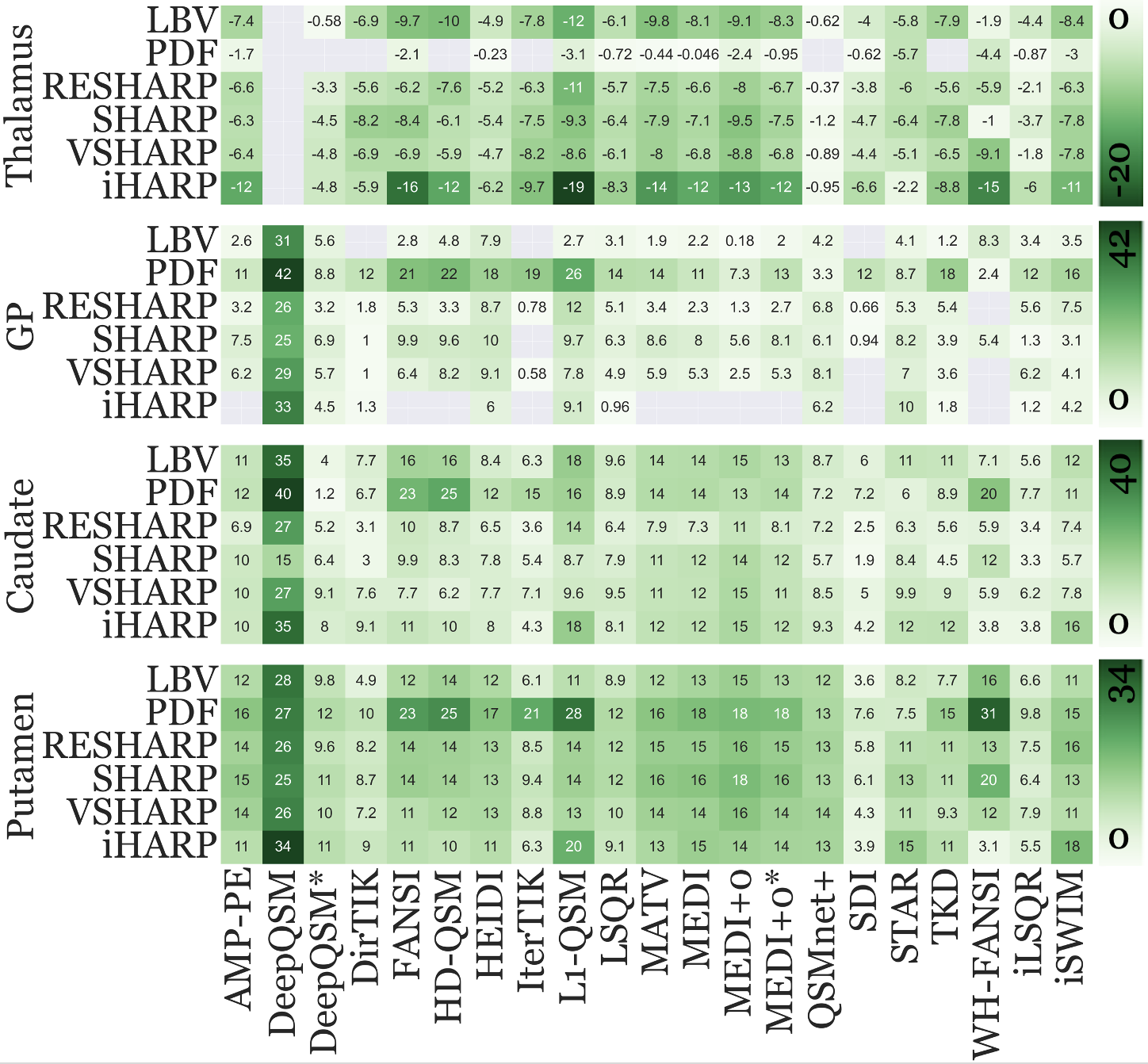
Supplementary Fig. 11. Raw WB-referenced temporal susceptibility (ppb) findings, respectively, of each pipeline. Each row corresponds to a BFR algorithm while each column represents an inversion algorithm. Each panel represents a DGM region with its title on the far left.


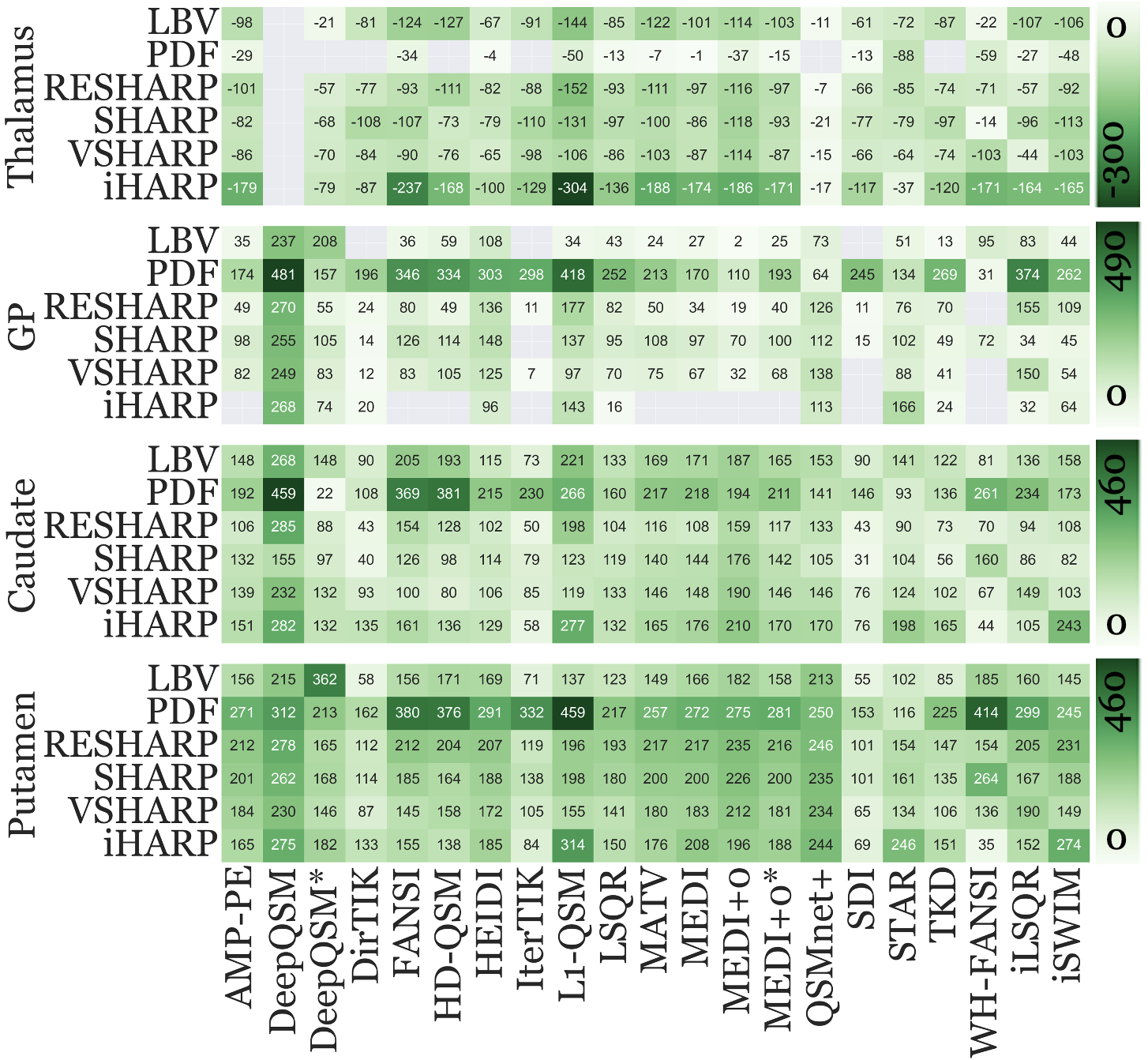
Supplementary Fig. 12. Normalized WB-referenced temporal susceptibility (ppb) findings, respectively, of each pipeline. Each row corresponds to a BFR algorithm while each column represents an inversion algorithm. Each panel represents a DGM region with its title on the far left.


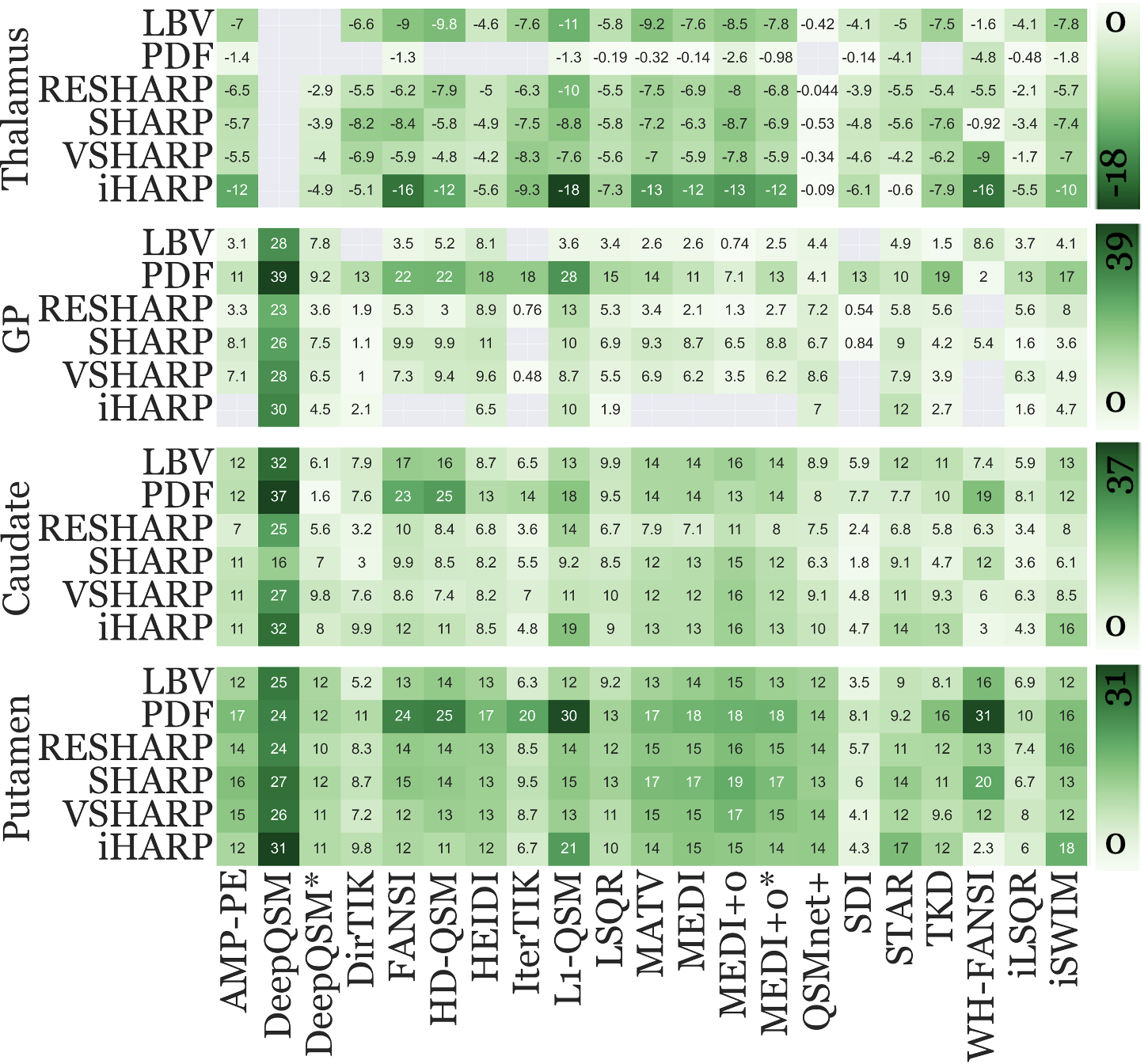
Supplementary Fig. 13. Raw WM-referenced temporal susceptibility (ppb) findings, respectively, of each pipeline. Each row corresponds to a BFR algorithm while each column represents an inversion algorithm. Each panel represents a DGM region with its title on the far left.


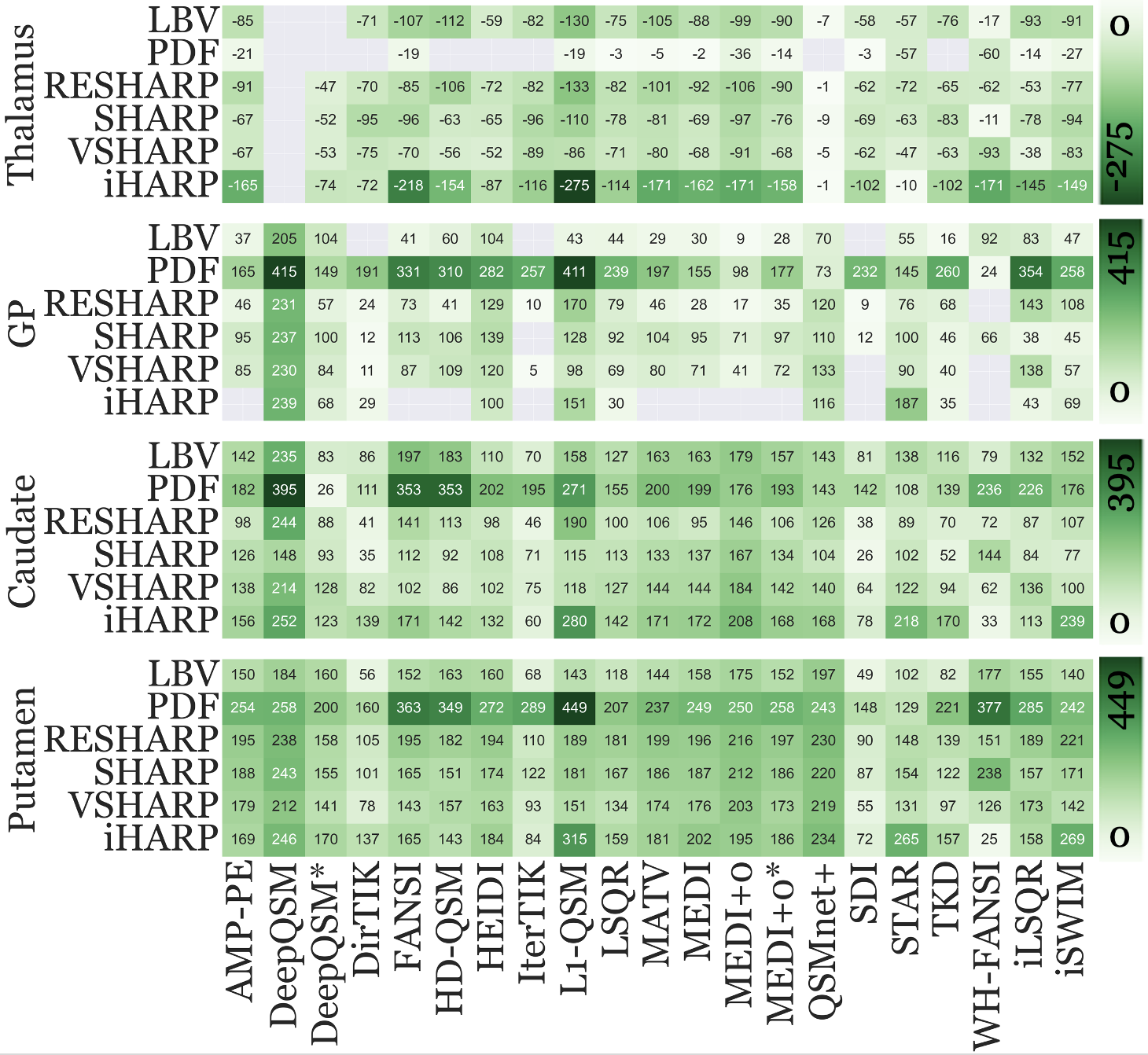
Supplementary Fig. 14. Normalized WM-referenced temporal susceptibility (ppb) findings, respectively, of each pipeline. Each row corresponds to a BFR algorithm while each column represents an inversion algorithm. Each panel represents a DGM region with its title on the far left.


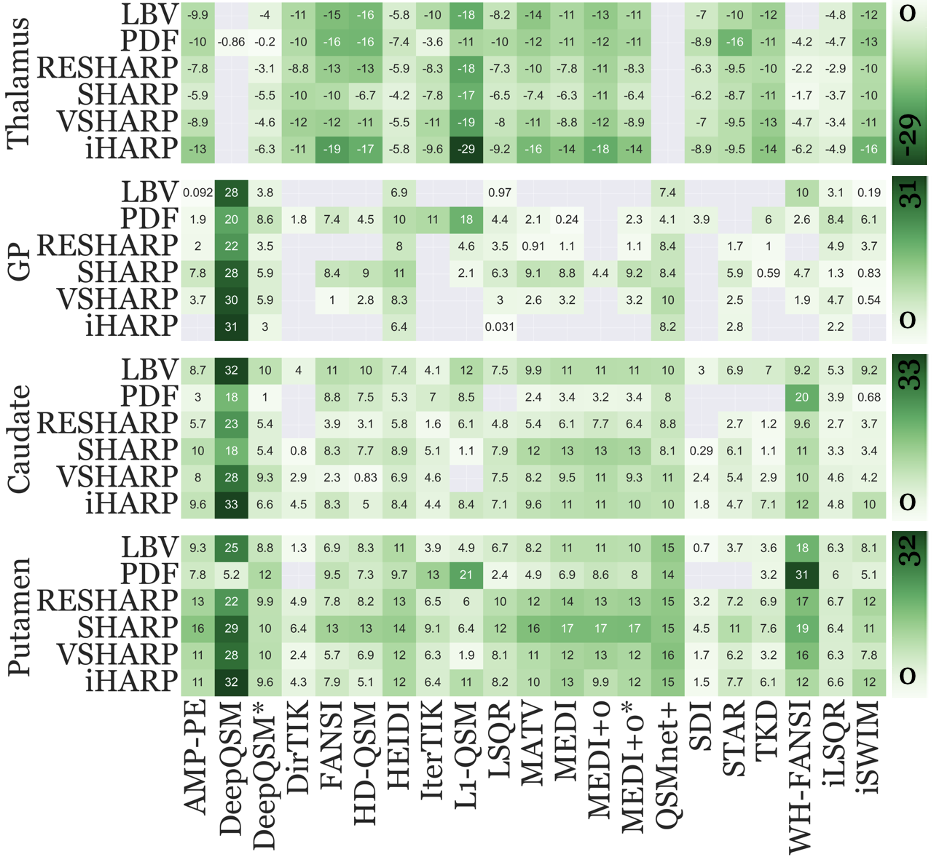
Supplementary Fig. 15. Raw CSF-referenced temporal susceptibility (ppb) findings, respectively, of each pipeline. Each row corresponds to a BFR algorithm while each column represents an inversion algorithm. Each panel represents a DGM region with its title on the far left.


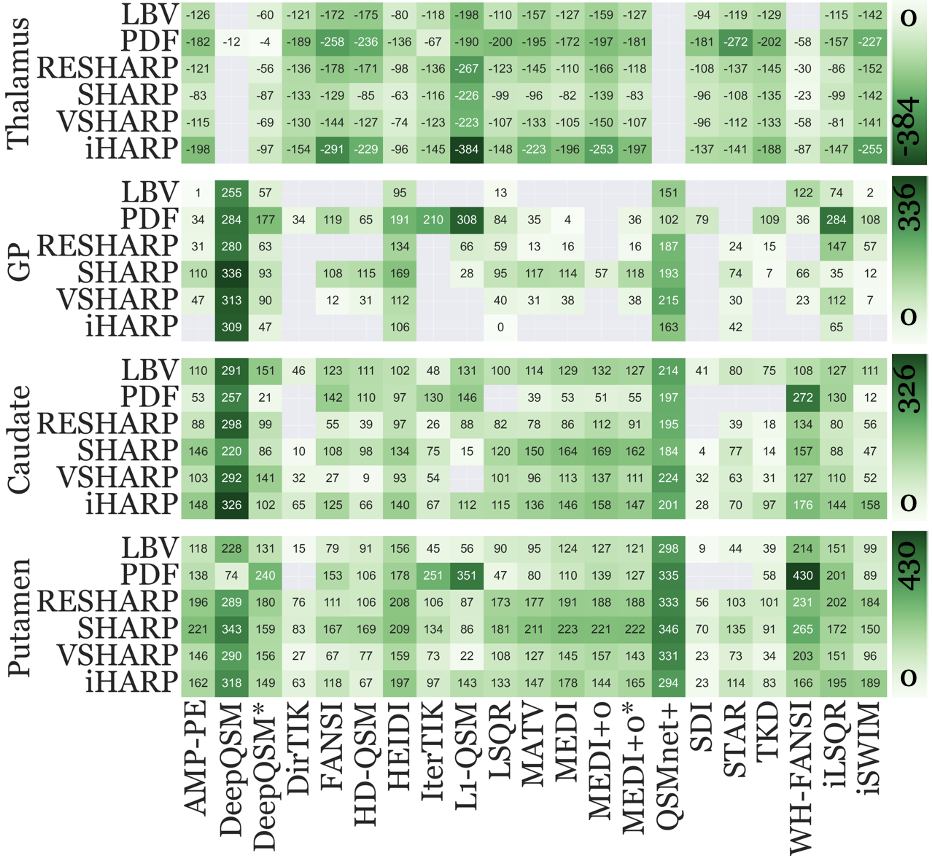
Supplementary Fig. 16. Normalized CSF-referenced temporal susceptibility (ppb) findings, respectively, of each pipeline. Each row corresponds to a BFR algorithm while each column represents an inversion algorithm. Each panel represents a DGM region with its title on the far left.


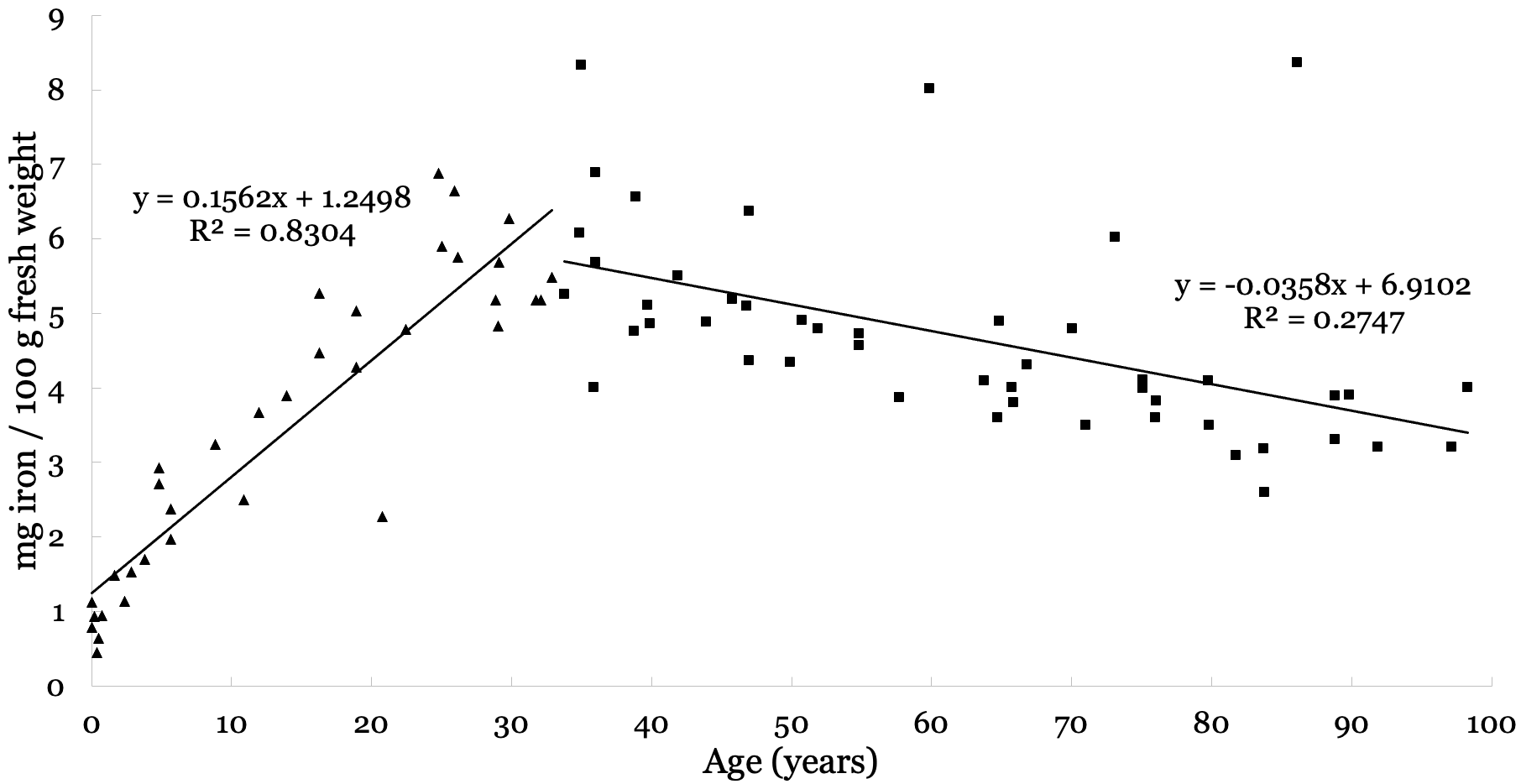


Supplementary Fig. 17. In-house obtained thalamus trajectory from H&S study (p. 47, Fig. 8 therein) using the WebPlotDigitizer tool. The age dependent equations and *R*^2^ correlations are displayed at the top of increasing and decreasing trendline, respectively.


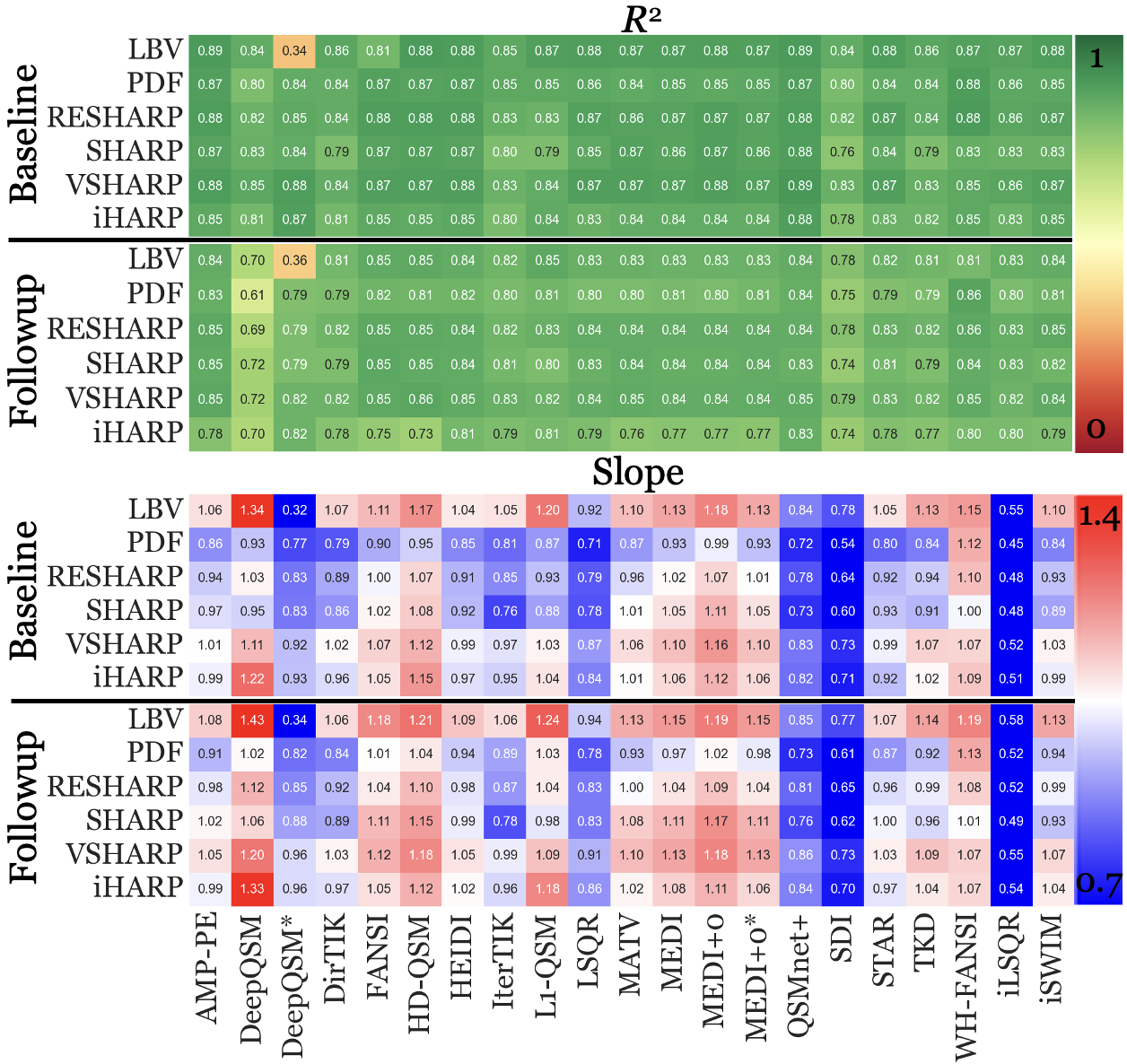
Supplementary Fig. 18. Correlation between observed DGM susceptibility and putative iron at baseline and follow-up timepoint, respectively. The *R*^2^ correlations are displayed at the top, while the slope is depicted at the bottom. Each row represents a BFR algorithms listed on the far left, while each row depicts an inversion algorithm listed at the bottom of the heatmap plot. Select pipeline plots can be viewed in Supplementary Fig. 20.


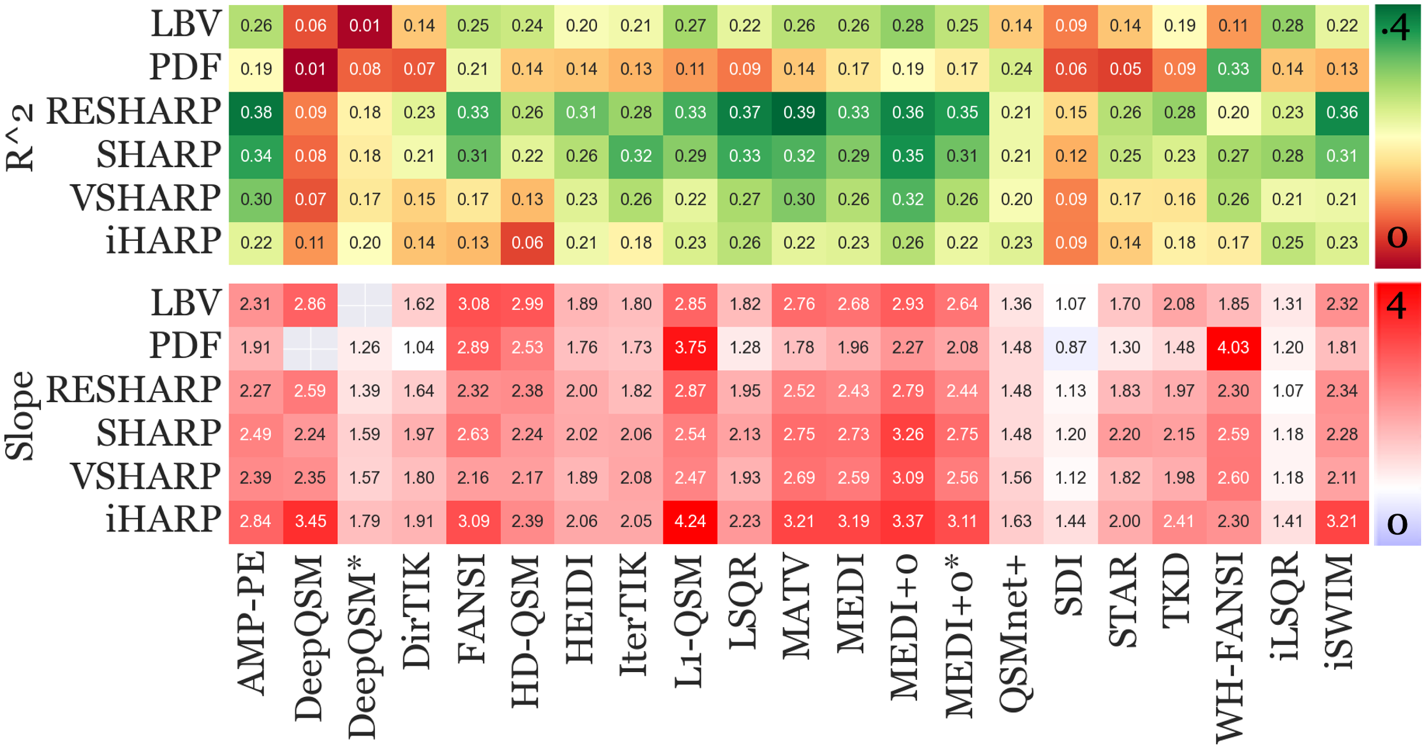
Supplementary Fig. 19 Correlation between observed DGM over-time susceptibility changes and putative iron changes. The R^2^ correlations are displayed at the top, while the slope is depicted at the bottom. Pipelines within the slope plot that exhibited non-significant correlation (p>0.05) were excluded (gray boxes). Select pipeline plots can be viewed in Supplementary Fig. 21.


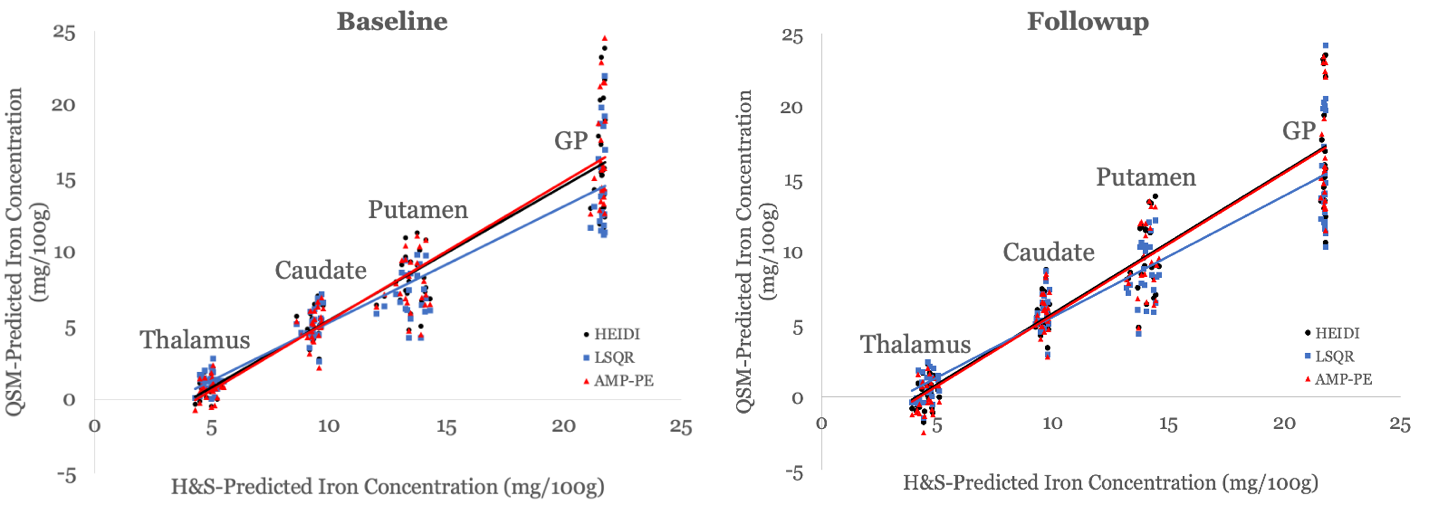
Supplementary Fig. 20. Correlation between observed DGM susceptibility and putative iron at baseline and follow-up timepoint, respectively, using pipelines in the 95^th^ percentile of the WB-referenced sensitivity metric (Supp. Fig. 25 – Top panel; RESHARP+AMP-PE, HEIDI, and LSQR). Their R^2^ correlations and slopes can be viewed in Supp. Fig 18. DGM regions are displayed on top of their data points.


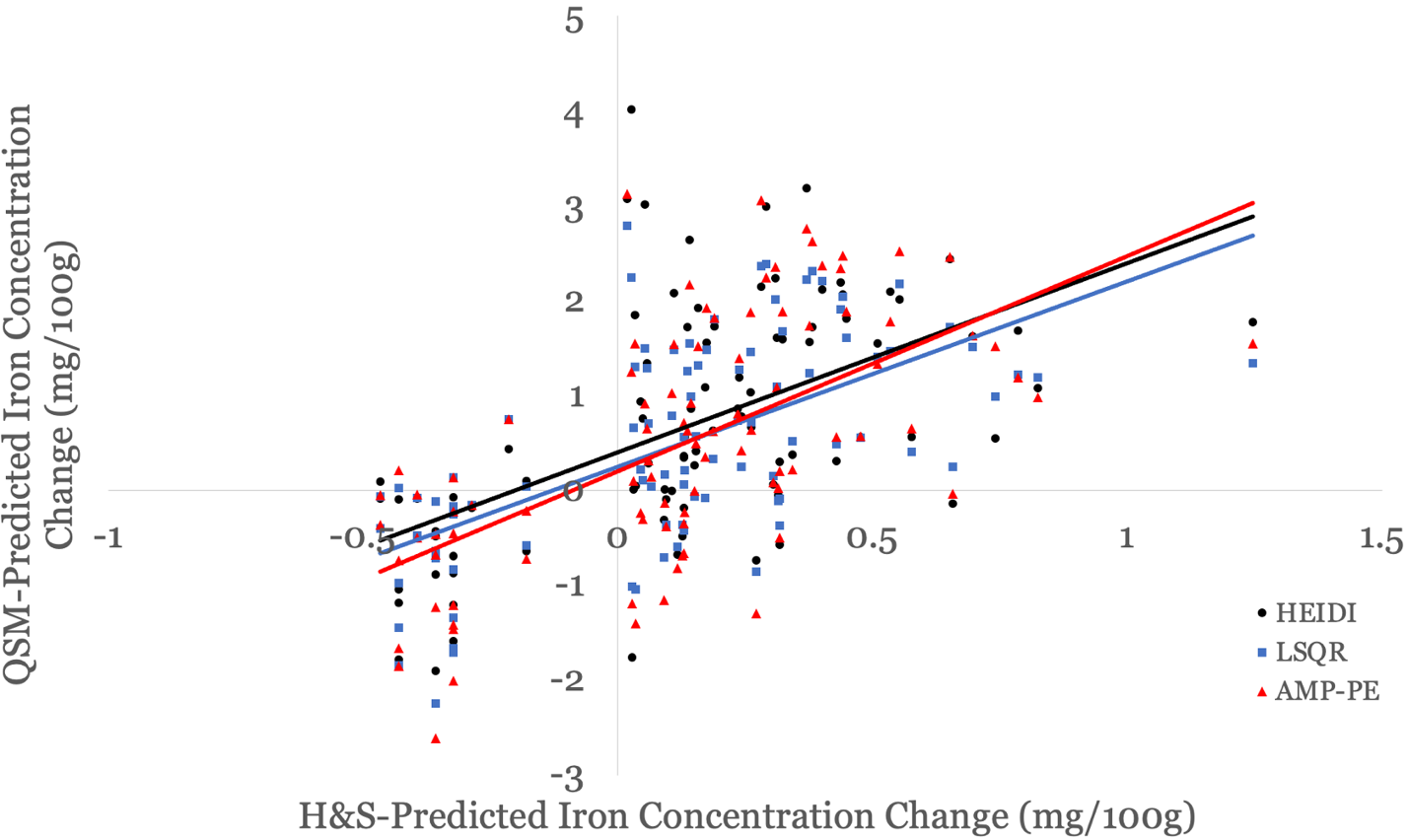
Supplementary Fig. 21. Correlation between observed DGM susceptibility and putative iron changes over-time using pipelines in the 95^th^ percentile of the WB-referenced sensitivity metric (Supp. Fig. 25 – Top panel; RESHARP+AMP-PE, HEIDI, and LSQR). Their R^2^ correlations and slopes can be viewed in Supp. Fig 19.


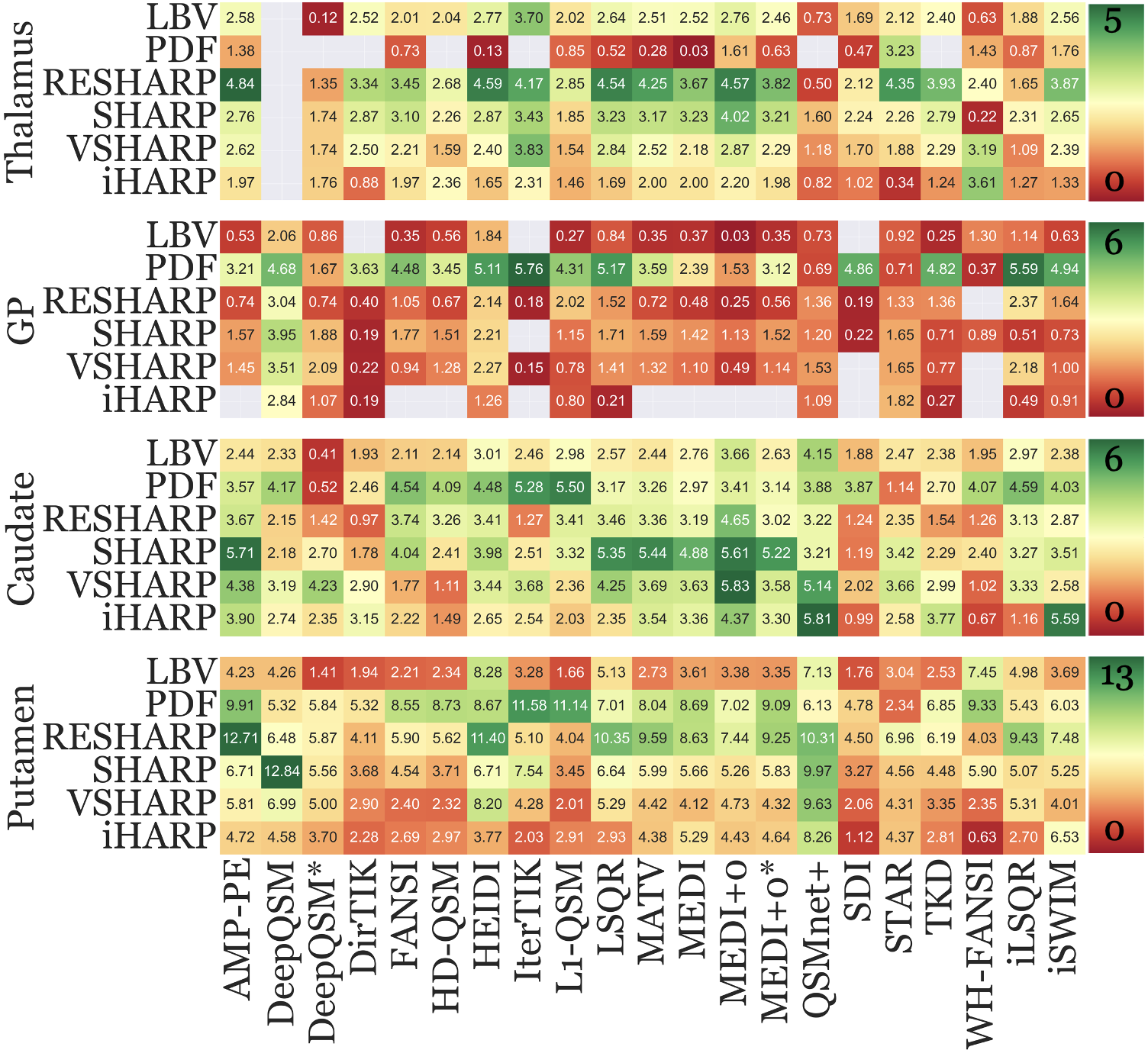
Supplementary Fig. 22. Pipeline sensitivity toward aging-related susceptibility changes using WB reference. Each row corresponds to a combination of a BFR algorithm and DGM region of interest (ROI). Each column represents an inversion algorithm. Susceptibility changes incompatible with H&S were excluded (gray box) to facilitate visualization. Each of the four regions (blocks of rows) has its own color bar on the right, with green indicating high sensitivity and red low.


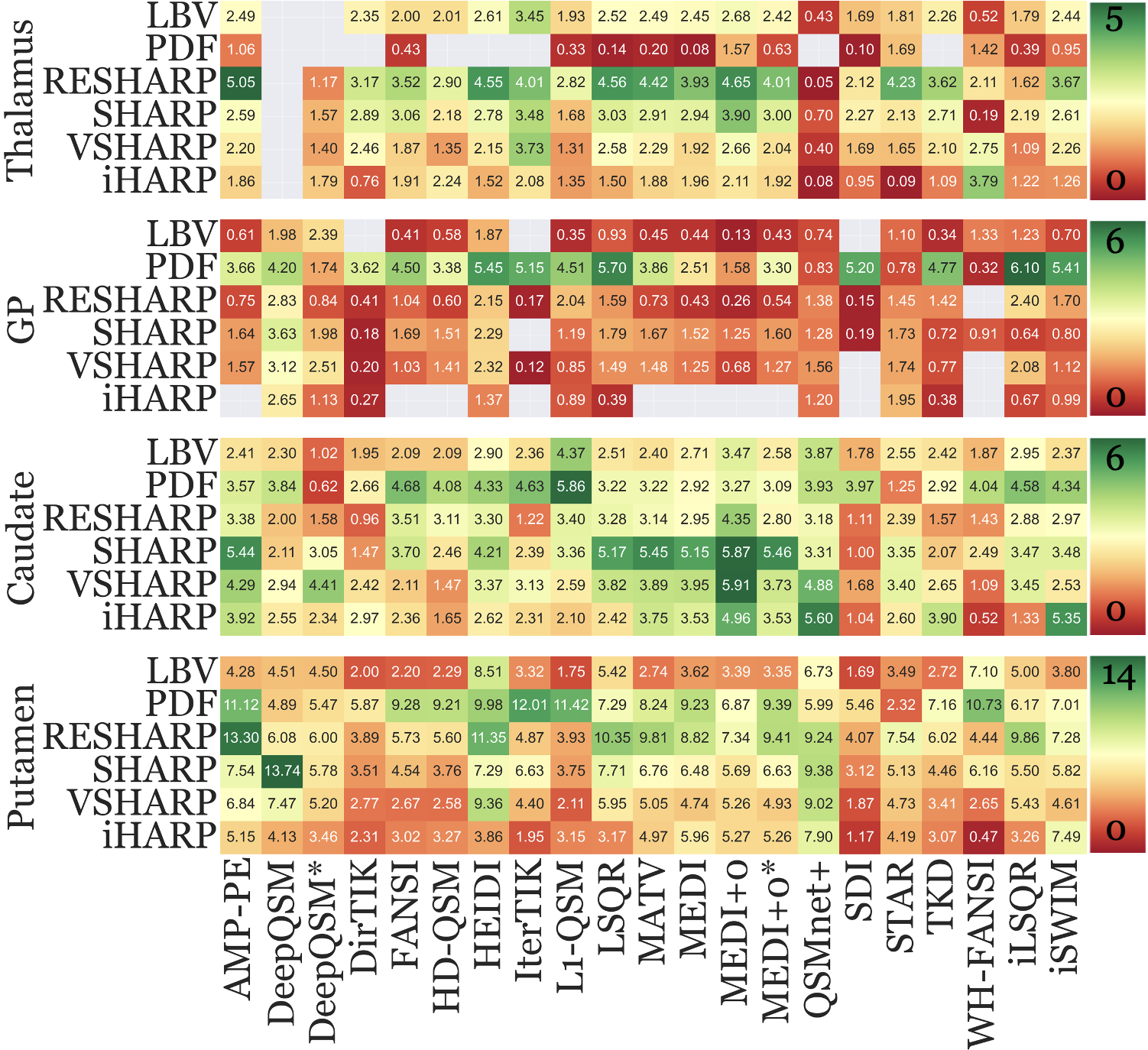


Supplementary Fig. 23. Pipeline sensitivity toward aging-related susceptibility changes using WM reference. Each row corresponds to a combination of a BFR algorithm and DGM region of interest (ROI). Each column represents an inversion algorithm. Susceptibility changes incompatible with H&S were excluded (gray box) to facilitate visualization. Each of the four regions (blocks of rows) has its own color bar on the right, with green indicating high sensitivity and red low.


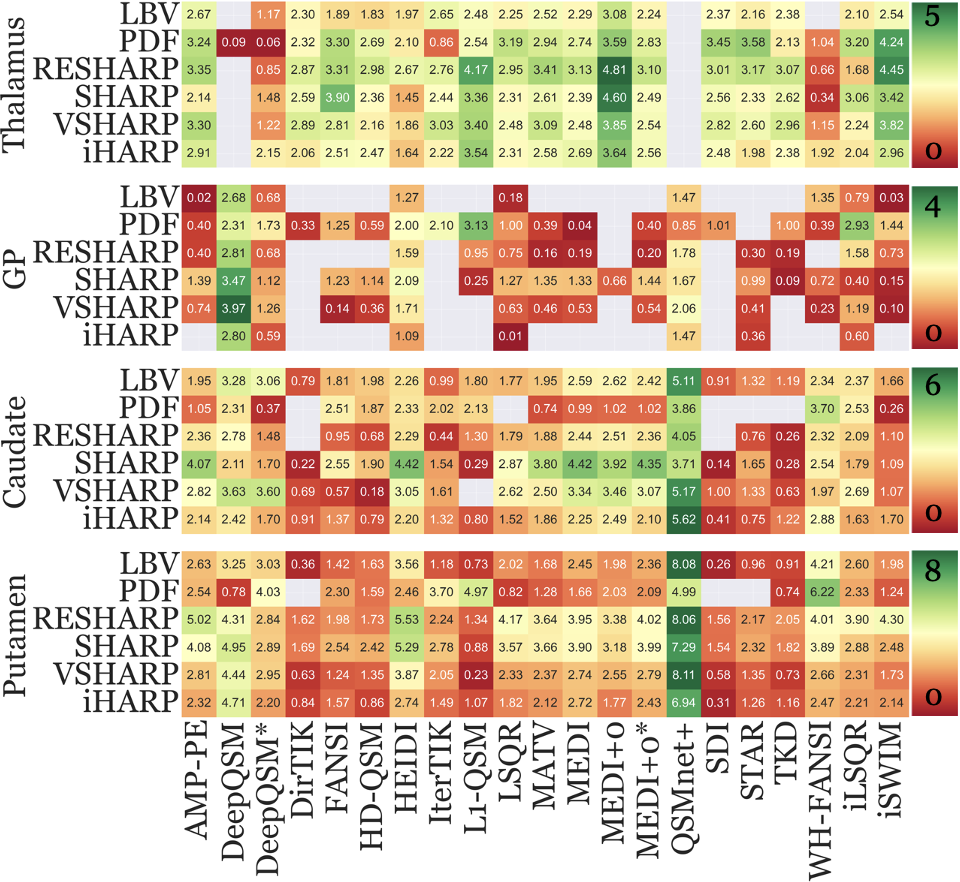
Supplementary Fig. 24. Pipeline sensitivity toward aging-related susceptibility changes using CSF reference. Each row corresponds to a combination of a BFR algorithm and DGM region of interest (ROI). Each column represents an inversion algorithm. Susceptibility changes incompatible with H&S were excluded (gray box) to facilitate visualization. Each of the four regions (blocks of rows) has its own color bar on the right, with green indicating high sensitivity and red low.


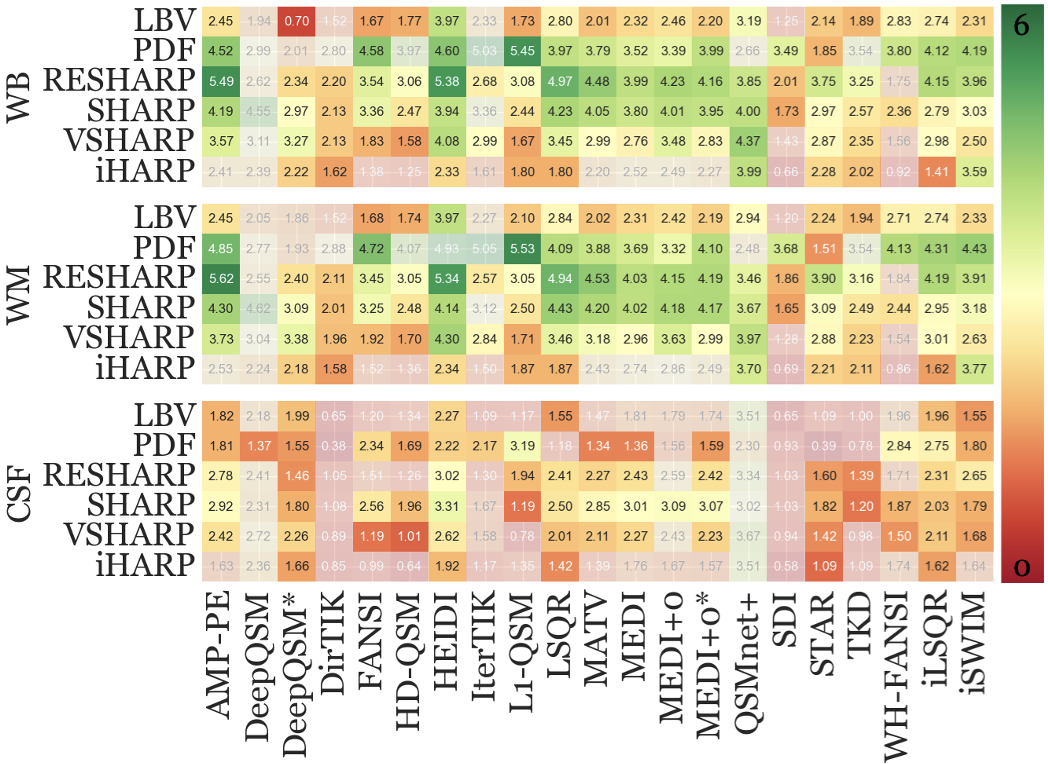


Supplementary Fig. 25. Global performance as defined in Eq. 7. Each row corresponds to a BFR algorithm, and each column represents an inversion algorithm. Pipelines that yielded regional changes incompatible with H&S in any of the regions are translucent instead of gray boxes (distinguishable by criss-cross within the boxes) in the other figures (due to numerous exclusions) to facilitate visualization. The color-coding and the arrangement of BFR and inversion algorithms mirrors that of supplementary Figs. 22-24.

**
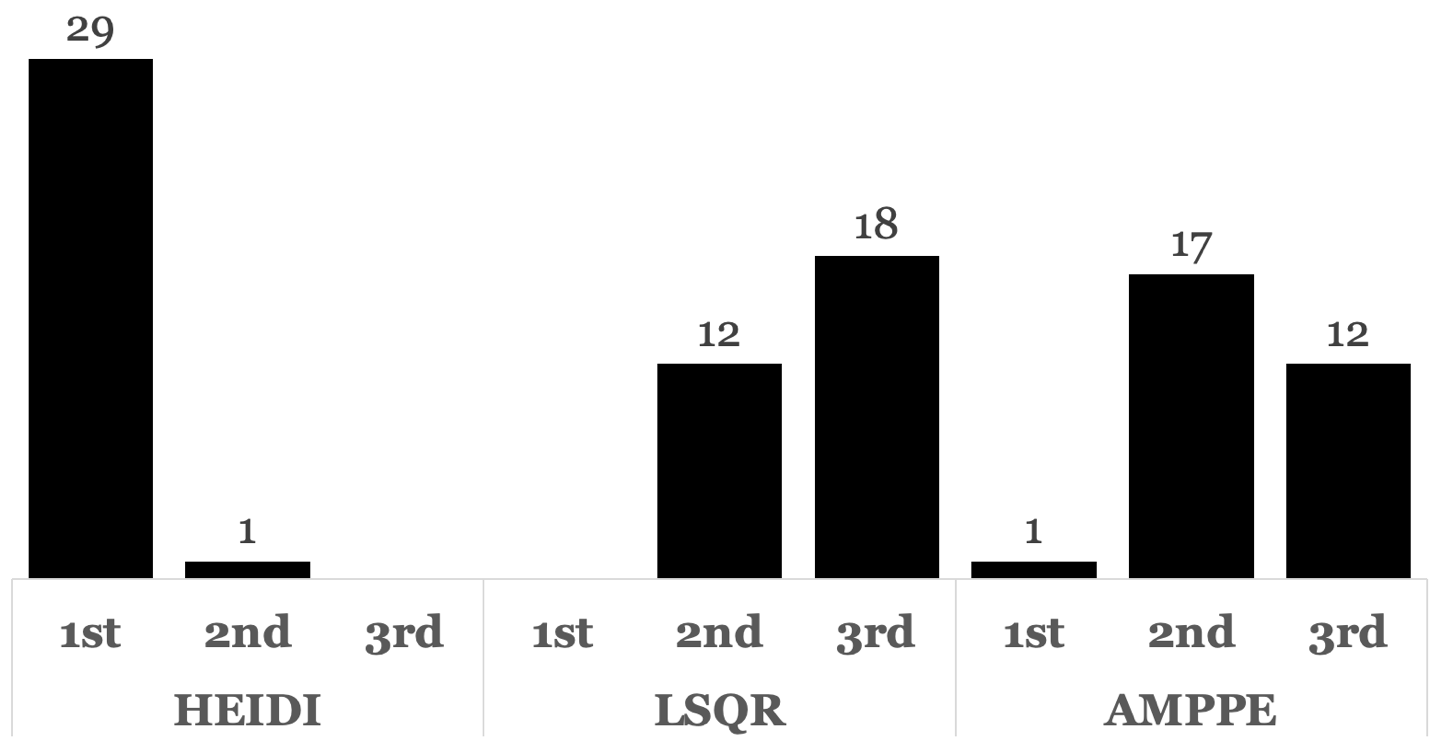
**

Supplementary Fig. 26. Ranking of 95^th^ sensitivity percentile pipelines over all subjects and raters. The maximum count was 30 (3 raters x 10 subjects).


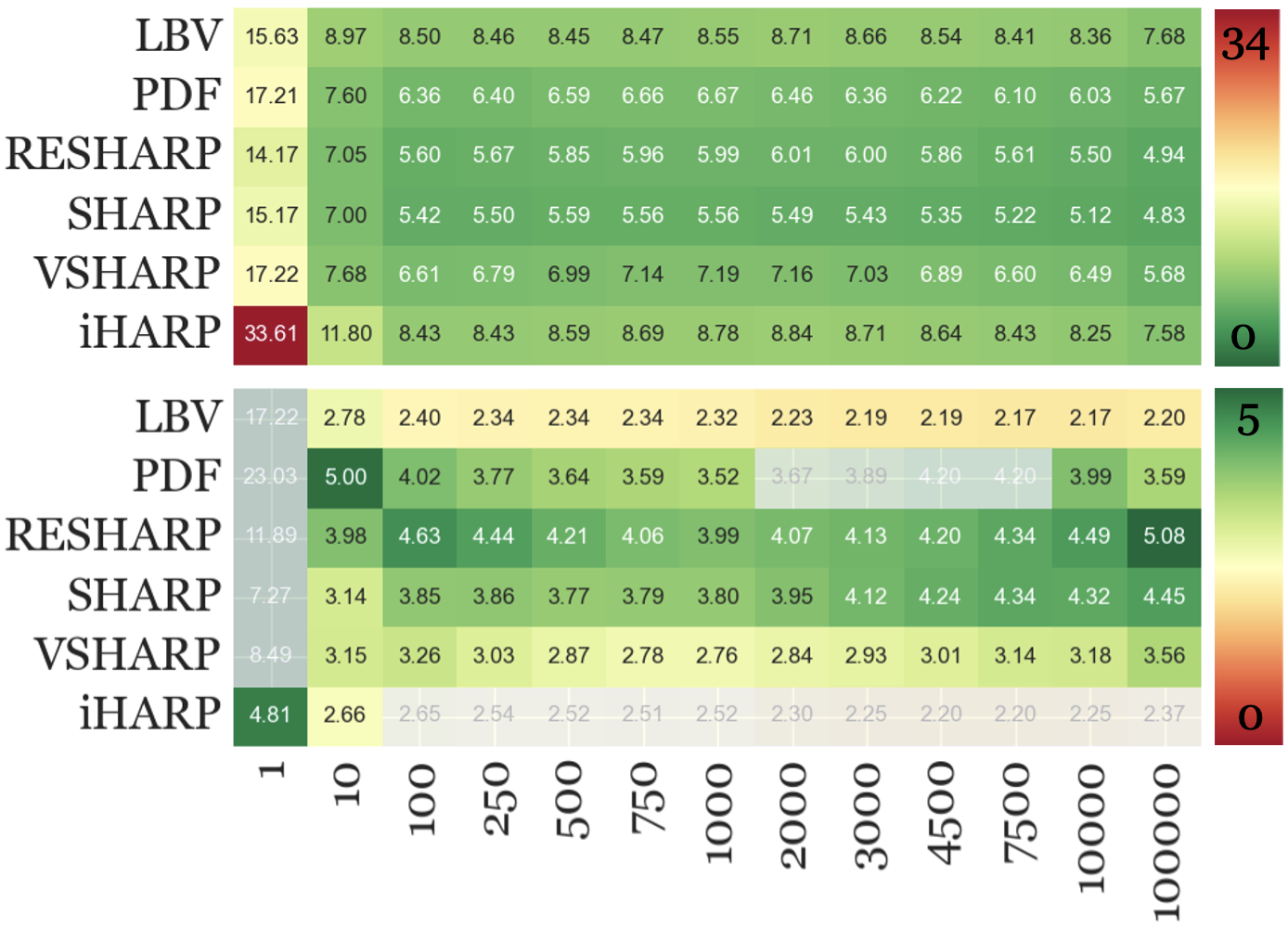


Supplementary Fig. 27. MEDI lambda parameter investigation on the reproducibility (top) and sensitivity (bottom) metric. Y-axis displays the choice of BFR while x-axis shows the lambda (regularization) parameter. Translucent-boxes stand for excluded pipelines due to inconsistent H&S-dependent overtime changes detected in one or more DGM regions.


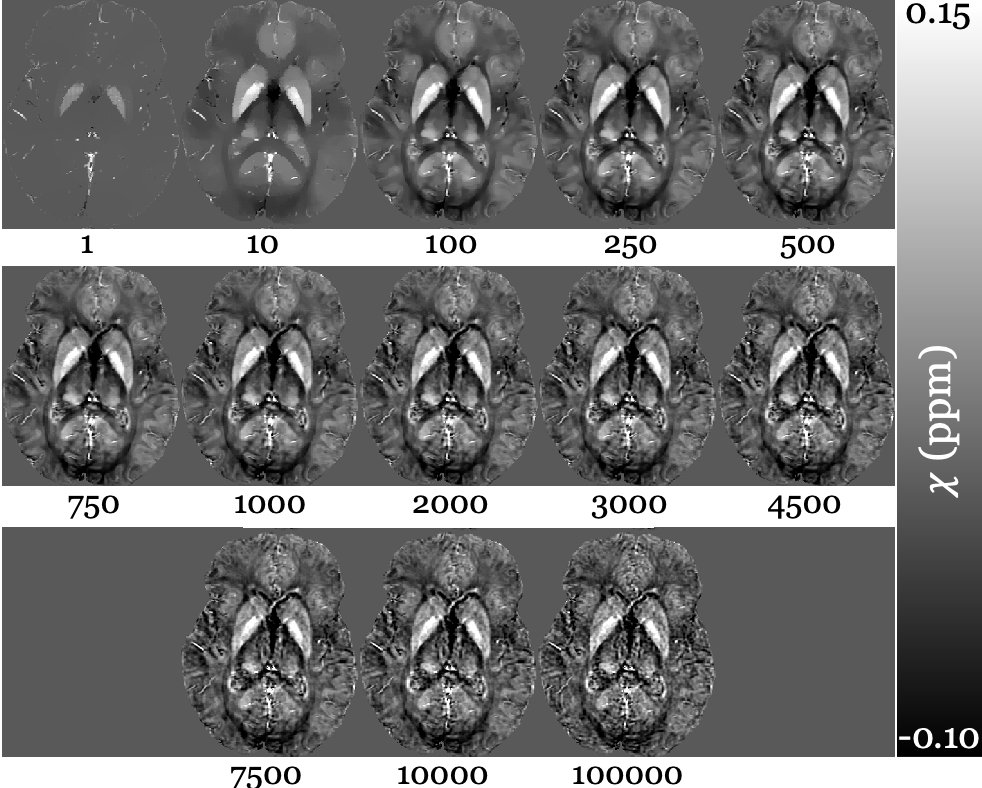
Supplementary Fig. 28. Susceptibility maps from a representative subject in native space using different MEDI lambda parameters with VSHARP as the BFR algorithm. The lambda (λ) parameters are displayed at the bottom of each susceptibility map.

Supplementary Tables

Supplementary Table 1. Algorithmic details in the following format: version number, year-date-month. NR=not reported.

| **BFR Algorithm** | |
| --- | --- |
| LBV | NR, 2013-06-24 |
| PDF | NR, 2013-07-24 |
| SHARP | v7.2q, 2014-09-09 |
| VSHARP | v7.2q, 2014-09-09 |
| iHARP | v3.0, 2017-05-NR |
| RESHARP | NR, 2017-06-14 |
| **Inversion Algorithm** | |
| HEIDI | v8.5, 2017-08-22 |
| FANSI | v2.0, 2021-10-15 |
| iSWIM | NR, 2014-01-04 |
| LSQR | v8.5, 2017-08-22 |
| MATV | v1.0, 2018-04-26 |
| MEDI | NR, 2014-12-15 |
| STAR | v3.0, 2017-05-NR |
| SDI | v3.2, 2021-08-17 |
| IterTIK | NR, 2021-07-12 |
| DirTIK | NR, 2021-07-12 |
| iLSQR | v3.0, 2017-05-NR |
| TKD | NR, 2021-07-12 |
| MEDI+0 | NR, 2017-11-06 |
| MEDI+0* | NR, 2017-11-06 |
| WH-FANSI | v2.0, 2021-10-15 |
| AMP-PE | NR, 2023-03-06 |
| QSMnet+ | NR, 2022-06-09 |
| DeepQSM* | NR, 2023-05-01 |
| L1-QSM | v2.0, 2021-10-15 |
| HD-QSM | NR, 2020-09-10 |
| DeepQSM | v1.0, 2020-03-16 |

Supplementary Table 2. Across-DGM median [with IQR] normalized reproducibility errors for reference regions and all algorithms (with each reference region).

|  | **Reference Region** | | |
| --- | --- | --- | --- |
|  | WB | WM | CSF |
|  | 6.65 [4.82] | 5.90 [4.25] | 9.73 [5.47] |
|  | **BFR Algorithm (Across Inversion Algorithms)** | | |
|  | WB | WM | CSF |
| LBV | 8.15 [2.91] | 7.26 [1.84] | 9.66 [2.09] |
| PDF | 6.20 [2.68] | 5.62 [2.40] | 10.36 [2.08] |
| SHARP | 5.85 [0.78] | 5.08 [0.91] | 8.64 [1.68] |
| VSHARP | 6.79 [1.48] | 5.86 [1.09] | 8.29 [1.66] |
| iHARP | 9.29 [2.47] | 8.58 [2.65] | 14.56 [3.04] |
| RESHARP | 5.90 [1.58] | 5.15 [1.29] | 9.10 [1.83] |
|  | **Inversion Algorithm (Across BFR Algorithms)** | | |
|  | WB | WM | CSF |
| HEIDI | 5.50 [0.70] | 4.79 [0.62] | 8.16 [2.15] |
| FANSI | 9.05 [3.50] | 7.99 [3.36] | 10.69 [1.98] |
| iSWIM | 6.48 [2.10] | 5.62 [1.99] | 9.40 [1.27] |
| LSQR | 5.23 [0.94] | 4.65 [0.97] | 8.41 [1.62] |
| MATV | 6.46 [2.13] | 5.60 [2.16] | 9.28 [2.20] |
| MEDI | 6.93 [2.05] | 5.98 [1.89] | 8.97 [2.13] |
| STAR | 6.38 [4.40] | 5.64 [4.77] | 9.38 [4.24] |
| SDI | 6.40 [1.06] | 5.64 [1.02] | 7.32 [1.14] |
| IterTIK | 5.12 [0.60] | 4.76 [0.66] | 8.71 [3.56] |
| DirTIK | 6.20 [0.33] | 5.64 [0.24] | 9.04 [2.93] |
| iLSQR | 7.17 [1.47] | 6.29 [1.57] | 9.96 [1.40] |
| TKD | 5.99 [0.55] | 5.61 [0.48] | 9.04 [3.48] |
| MEDI+0 | 6.70 [1.71] | 5.87 [1.56] | 9.51 [1.43] |
| MEDI+0* | 6.58 [2.14] | 5.71 [1.95] | 9.11 [1.81] |
| WH-FANSI | 8.04 [1.13] | 7.02 [0.81] | 9.94 [0.72] |
| AMP-PE | 5.54 [2.05] | 4.86 [1.95] | 8.15 [1.49] |
| QSMnet+ | 5.10 [0.49] | 4.71 [0.49] | 7.47 [0.73] |
| DeepQSM* | 8.34 [1.88] | 7.22 [1.80] | 9.70 [1.39] |
| L1-QSM | 11.61 [3.53] | 10.08 [2.91] | 15.37 [3.88] |
| HD-QSM | 9.40 [2.82] | 8.62 [2.21] | 11.89 [1.42] |
| DeepQSM | 10.80 [2.93] | 9.75 [2.82] | 12.86 [2.46] |

Supplementary Table 3. Comments from raters on the visual appearance of the susceptibility maps in the 95^th^ percentile (RESHARP+AMP-PE, HEIDI, and LSQR).

|  | AMP-PE | HEIDI | LSQR |
| --- | --- | --- | --- |
| Rater 1 | Streaking artifacts in the sagittal plane (not as many as LSQR), blurry, pixelated | Sharp, less blurry | Noisy, streaking artifacts in the sagittal plane, inhomogeneous, WM veins visible, not natural. |
| Rater 2 | Reconstruction artifacts | Homogeneous, no visible reconstruction artifacts | Inhomogeneous |
| Rater 3 | Pixelated, too blocky | Homogenous, the best | Streaking artifacts in the coronal plane, not physiological, inconsistent gray matter and WM contrast (inhomogeneous), cloudy, tissue boundaries not sharp |
